# Impaired mitophagy links mitochondrial disease to epithelial stress in methylmalonyl-CoA mutase deficiency

**DOI:** 10.1038/s41467-020-14729-8

**Published:** 2020-02-20

**Authors:** Alessandro Luciani, Anke Schumann, Marine Berquez, Zhiyong Chen, Daniela Nieri, Mario Failli, Huguette Debaix, Beatrice Paola Festa, Natsuko Tokonami, Andrea Raimondi, Alessio Cremonesi, Diego Carrella, Patrick Forny, Stefan Kölker, Francesca Diomedi Camassei, Francisca Diaz, Carlos T. Moraes, Diego Di Bernardo, Matthias R. Baumgartner, Olivier Devuyst

**Affiliations:** 1 Institute of Physiology and NCCR Kidney.CH, University of Zurich, 8057 Zurich, Switzerland; 2 Division of Metabolism and Children’s Research Center, University Children’s Hospital, 8032 Zurich, Switzerland; 3 Department of Biomedicine, University of Eastern Finland, 70211 Kuopio, Finland; 4 San Raffaele Scientific Institute, Experimental Imaging Center, 20132 Milan, Italy; 5 Division of Clinical Chemistry and Biochemistry, University Children’s Hospital Zurich, 8032 Zurich, Switzerland; 6 Telethon Institute of Genetics and Medicine, Pozzuoli, 80078 Naples, Italy; 7 Division of Inherited Metabolic Diseases, University Children’s Hospital Heidelberg, 69120 Heidelberg, Germany; 8 Department of Laboratories–Pathology Unit, Bambino Gesù Children’s Hospital, 00165 Rome, Italy; 9 Department of Neurology, University of Miami Miller School of Medicine, 33136 Miami, FL USA; 10 Division of Nephrology, Cliniques Universitaires Saint-Luc, 1040 Brussels, Belgium

**Keywords:** Mechanisms of disease, Autophagosomes, Mitochondria, Chronic kidney disease

## Abstract

Deregulation of mitochondrial network in terminally differentiated cells contributes to a broad spectrum of disorders. Methylmalonic acidemia (MMA) is one of the most common inherited metabolic disorders, due to deficiency of the mitochondrial methylmalonyl-coenzyme A mutase (MMUT). How *MMUT* deficiency triggers cell damage remains unknown, preventing the development of disease–modifying therapies. Here we combine genetic and pharmacological approaches to demonstrate that *MMUT* deficiency induces metabolic and mitochondrial alterations that are exacerbated by anomalies in PINK1/Parkin–mediated mitophagy, causing the accumulation of dysfunctional mitochondria that trigger epithelial stress and ultimately cell damage. Using drug–disease network perturbation modelling, we predict targetable pathways, whose modulation repairs mitochondrial dysfunctions in patient–derived cells and alleviate phenotype changes in *mmut*–deficient zebrafish. These results suggest a link between primary *MMUT* deficiency, diseased mitochondria, mitophagy dysfunction and epithelial stress, and provide potential therapeutic perspectives for MMA.

## Abstract (web summary)

Methylmalonic acidemia is an inherited metabolic disease caused by loss or mutation of the enzyme MMUT. Here the authors use cell and animal models to show that MMUT mutations lead to defective mitophagy and stress in kidney cells, contributing to the pathogenesis in methylmalonic acidemia patients.

## Introduction

Mitochondria—the intracellular powerhouse in which energy from nutrients is converted into ATP—are highly dynamic, double-membraned organelles that sustain cellular metabolism and physiology^1,2^. The maintenance of these dynamic and functionally pleiotropic organelles is particularly relevant in terminally differentiated cells that are highly dependent on aerobic metabolism^3^. Genetic dysfunctions of the mitochondrial network and homeostasis might therefore confer a potentially devastating vulnerability to many different cells contributing to a broad spectrum of diseases^4^.

Mitochondrial diseases are among the most common type of inherited metabolic disorders, which often manifest in early childhood and are associated with high morbidity and mortality^5,6^. Methylmalonic acidaemia (MMA; MIM #251000)—the most common form of organic aciduria^7,8^—is caused by recessive, inactivating mutations in the *MMUT* gene encoding the mitochondrial enzyme methylmalonyl-coenzyme A mutase (MMUT) that mediates the terminal step of branched-chain amino acid metabolism^9^. Complete (*mmut*^0^) and/or partial (*mmut*^–^) loss of MMUT function results in the accumulation of toxic metabolites (e.g. methylmalonic acid [MMA], propionic acid and 2-methylcitric acid) within mitochondrial matrix that trigger ultrastructural (e.g. presence of megamitochondria with abnormal cristae; ref. ^10^) and/or functional (e.g. abnormal mitochondrial energetic and redox profiling) mitochondrial alterations^10,11^, causing severe organ dysfunctions that primarily affect brain, liver and kidney^12,13^. The mechanisms linking *MMUT* deficiency to mitochondrial dysfunctions and cell toxicity remain largely unknown, restricting therapeutic avenues for this devastating disorder to supportive care^14^.

The epithelial cells that line kidney tubules are enriched in mitochondria, whose energy production maintains transport functions and overall kidney integrity^15^. Disruption of mitochondrial homeostasis in inherited mitochondrial cytopathies drives various degrees of epithelial (tubular) dysfunction and kidney disease^16^. For instance, a systematic study of 42 patients with mitochondrial disorders showed that 21 patients had kidney tubular dysfunction and 8 had renal failure, confirming the underestimated prevalence of kidney involvement in these disorders^17^. Conversely, modulating mitochondrial function might restore kidney function in mouse models of acute^18^ and chronic kidney disease^19^.

Cells possess quality control systems to maintain a requisite number of functional mitochondria to meet the energy demands^20^. These pathways concur to eliminate damaged mitochondrial proteins or dysfunctional parts of mitochondrial network by autophagy (aptly termed mitophagy; ref. ^21^). Biochemical and genetic evidences reveal that the PTEN-induced putative kinase1 (PINK1) and Parkin are the key drivers of mitophagy, driven by the loss of mitochondrial membrane potential^22^. This homoeostatic mitochondrial process is particularly active in kidney tubular cells^23^. Deletion of genes encoding mitophagy-promoting molecules damages tubular cells through defective mitochondrial clearance and increased reactive oxygen species (ROS)^24^. Abnormal mitochondria with disorganized cristae have been described in kidney cells^25^ and biopsies from MMA patients^10,26^, suggesting an involvement of mitochondrial quality control mechanisms in the disease.

In the present study, using MMA as a paradigm of complex mitochondrial dysfunction, we decipher a pathway that links loss-of-function of a mitochondrial enzyme, mitochondrial abnormalities, defective PINK1/Parkin-mediated quality control and mitochondria-derived stress in kidney tubular cells. These insights offer promising therapeutic avenues for modulating mitochondrial function and epithelial cell damage in MMA.

## Results

### *MMUT* deficiency impairs mitochondria in kidney tubular cells

**Fig. 1:**
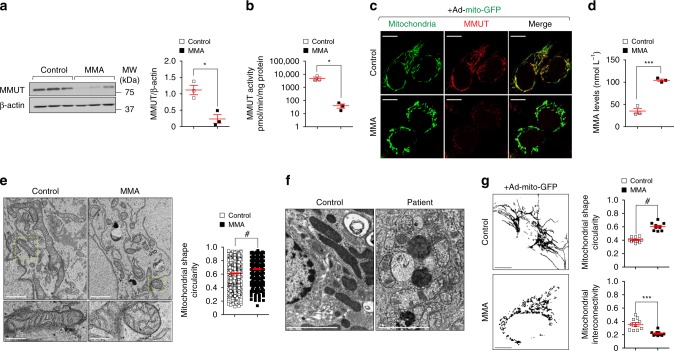
Abnormal mitochondrial network in MMA kidney cells. **a** Immunoblotting and quantification of MMUT and **b** its enzyme activity in control and MMA kidney cells, *n* = 3 biologically independent experiments. β-actin was used as a loading control. **c**, **g** Cells were transduced with adenoviral particles carrying the mitochondrially targeted green fluorescent protein (Ad-mito-GFP, green). **c** After 24 h post-transduction, the cells were immunostained for MMUT (red) and imaged by confocal microscopy. Yellow indicates the colocalization. **d** Quantification of methylmalonic acid (MMA) levels by liquid chromatography tandem mass spectrometry (LC-MS/MS, *n* = 3 biologically independent samples). **e** Representative electron micrographs and quantification of the shape (expressed as circularity) of the mitochondrial network in control and MMA cells; *n* *=* 527 mitochondria pooled from 24 control cells and *n* = 310 mitochondria pooled from 20 MMA cells. Values are pooled from two biologically independent experiments. Dotted yellow squares represent regions of the respective panels magnified. **f** Representative electron micrographs showing the mitochondrial network in kidney biopsies from an individual healthy control subject and a patient with MMA. **g** Representative inverted images (left) and quantification of shape (expressed as circularity, top right) or interconnectivity (bottom right) of the mitochondrial network in control and MMA cells; each point representing the average values for circularity or interconnectivity in a cell, *n* = 10 cells per each condition pooled from two biologically independent experiments. Plots represent mean ± SEM. Two-tailed Student’s *t*-test, *P < 0.05, ***P < 0.001 and ^#^*P* < 0.0001 relative to control cells. Scale bars are 10 μm in **c** and **g**, and 1 μm (top) and 250 nm (bottom) in **e** and 2 μm in **f**. Unprocessed scans of original blots are shown in Supplementary Fig. 13. Source data are provided as a Source Data file.

As MMUT is robustly expressed within the mitochondria of kidney tubular cells (Supplementary Fig. 1a‒e), we first investigated the consequences of *MMUT* deficiency on mitochondrial function and homeostasis in these cells. To this aim, we analysed the properties of mitochondrial network in kidney tubular cells derived from the urine of either healthy controls or *mut*^*0*^ MMA patients harbouring inactivating mutations in *MMUT* (Supplementary Table 1; ref. ^25^). Compared to their control cells, the MMA patient-derived kidney tubular cells (hereafter referred to as MMA cells) exhibited a marked decrease in MMUT protein (Fig. 1a) and in its mitochondrial enzymatic activity (Fig. 1b, c), reflected by the accumulation of methylmalonic acid (MMA; Fig. 1d). Transmission electron microscopy (TEM) analyses revealed that mitochondria, which appear as an interconnected meshwork of elongated or curvilinear organelles in control cells, were fragmented or characterized by a prominent rod-like shape with perturbed cristae organization in MMA cells (Fig. 1e) and in the kidneys of a patient with MMA (Fig. 1f), in line with recent studies showing an abnormal mitochondrial ultrastructure in both kidney and explanted livers of patients with MMA^26^. Confocal microscopy of the mitochondrially targeted green fluorescent protein (mito-GFP) and semi-automated image analyses confirmed in MMA cells the presence of mitochondria which appear circular and robustly fragmented when compared to control cells (Fig. 1g).

**Fig. 2:**
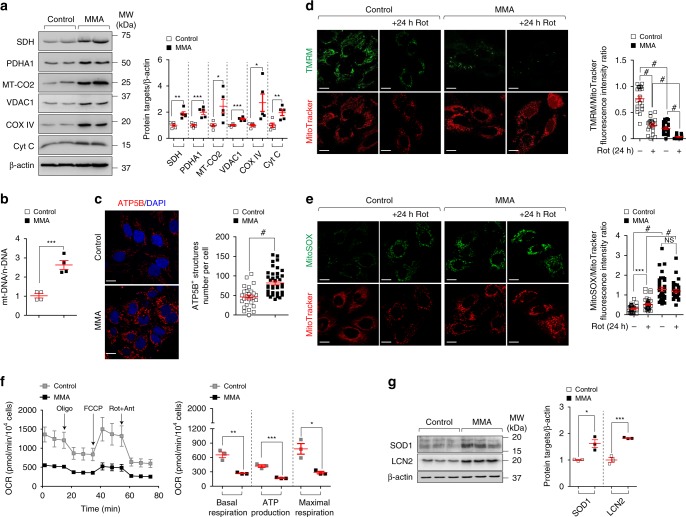
Mitochondrial dysfunctions in MMA kidney cells. **a** Representative immunoblotting and quantification of the indicated mitochondrial proteins; *n* = 5 biologically independent experiments. **b** The ratio between mitochondrial DNA (*ND1*) and nuclear DNA (*ACTB*) was determined by quantitative PCR; *n* = 4 biologically independent experiments. **c** Cells were immunostained for ATP5B (red) and imaged by confocal microscopy. Representative images and quantification of numbers of ATP5B^+^ structures per cell (*n* = 27 control cells and *n* = 40 MMA cells pooled from three biologically independent experiments). Nuclei counterstained with DAPI (blue). **d**, **e** Cells were exposed to mitochondrial complex I inhibitor Rotenone (Rot, 5 μM). After 24 h of treatment, the cells were loaded (**d**) with tetramethylrhodamine methyl ester (TMRM; green; mitochondrial membrane potential fluorescent probe, 50 nM for 30 min at 37 °C) and MitoTracker (red; fluorescent probe that localizes to mitochondria; 1 μM for 30 min at 37 °C) or (**e**) with MitoSOX (green; mitochondrial ROS indicator, 2.5 μM for 25 min at 37 °C) and MitoTracker (red), and analysed by confocal microscopy. Representative images and quantification of **d** membrane potential and **e** mitochondrial ROS (both calculated as ratio between TMRM and MitoTracker or MitoSOX and MitoTracker fluorescence intensities, with each point representing the average fluorescence intensity ratio in a cell). TMRM/MitoTracker: *n* = 21 untreated and Rot-treated control cells, *n* = 22 untreated MMA cells and *n* = 18 Rot-treated MMA cells. MitoSOX/MitoTracker: *n* = 31 untreated control cells and *n* = 40 Rot-treated control cells *n* = 36 untreated MMA cells and *n* = 31 Rot-treated MMA cells. Values are pooled from three biologically independent experiments. **f** Oxygen consumption rate (OCR) and individual parameters for basal respiration, ATP production and maximal respiration. OCRs were measured at baseline and after the sequential addition of Oligomycin (Oligo, 1 μM), FCCP (0.5 μM) and Rotenone (Rot; 1 μM) + Antimycin A (Ant; 1 μM), *n* = 3 biologically independent experiments. **g** Immunoblotting and quantification of the indicated proteins; *n* = 3 biologically independent experiments. Plots represent mean ± SEM. Two-tailed Student’s *t*-test, *P < 0.05, **P < 0.01, ***P < 0.001 and ^#^*P* < 0.0001 relative to untreated or to Rot-treated control cells or to untreated MMA cells. β-actin was used as a loading control in **a** and **b**. Scale bars, 10 μM. NS non-significant. Source data are provided as a Source Data file.

The morphological abnormalities prompted us to examine whether *MMUT* deficiency alters the homeostasis of the mitochondrial network. Using immunoblotting analyses for mitochondrial proteins that label outer (e.g. VDAC1) and inner membrane (e.g. SDH, MT-CO2 and COX IV), intermembrane space (e.g. Cyt C) and matrix (e.g. PDHA1), we noted an increased abundance of overall mitochondrial proteins in MMA compared to control cells (Fig. 2a). These changes were confirmed by measuring the ratio between mitochondrial (mt-DNA) and nuclear (n-DNA) through quantitative PCR analyses (Fig. 2b) and by quantifying the ATP5B-flagged mitochondrial structures using confocal microscopy (Fig. 2c). These mitochondrial alterations did not result from an effect of *MMUT* deficiency on cell viability (Supplementary Fig. 1f) and proliferation (Supplementary Fig. 1g), nor transcriptional changes in mitochondrial genes (*MFN1/2, DRP1, OPA1*) regulating fusion/fission process (Supplementary Fig. 1h), which were similar between MMA and control cells. Taken together, these data indicate that the deficiency of *MMUT* alters the homeostasis of the mitochondrial network, hence increasing the content of mitochondria organelles.

### Mitochondrial dysfunction drives stress in MMA cells

As *MMUT* deficiency alters mitochondrial homeostasis, we next assessed potential consequences on mitochondrial function. Consistent with increased numbers of morphologically aberrant mitochondria, the mitochondrial membrane potential (Δψ_m_) was drastically reduced in MMA cells (Fig. 2d), as evidenced by live cell imaging analyses of the mitochondrial network with cell-permeant, fluorescent dye tetramethylrhodamine methyl ester (TMRM, which readily accumulates within functional mitochondria) and MitoTracker (a fluorescent probe that localizes to mitochondria). These changes were paralleled by a major mitochondrial oxidative stress (Fig. 2e), as testified by the elevated production of mitochondria (mt)-derived ROS (MitoSOX, a live-cell-permeant indicator of mitochondrial ROS) and augmented antioxidant response (SOD1; Fig. 2g). Treatment with the mitochondrial complex I inhibitor Rotenone (5 μM for 24 h), which did not alter the cell viability, exacerbated mitochondrial alterations (e.g. decreased mitochondrial membrane potential and increased mt-ROS levels) to a greater extent in MMA compared to control cells (Fig. 2d, e). Seahorse metabolic flux analyses measuring oxygen consumption rate (OCR) confirmed impaired mitochondrial bioenergetics in *MMUT*-deficient cells, as evidenced by a significant reduction in the baseline respiration, ATP turnover and total respiratory capacity (Fig. 2f), suggesting that *MMUT* deficiency impinges on the function and homeostasis of mitochondrial network in both normal and stress-evoked conditions.

Previous studies in transgenic *Mmut*-deficient mice and in a large cohort of patients with MMA^10,26^ showed that mitochondrial dysfunction and oxidative stress are linked to increased production of lipocalin-2 (LCN2, also known as NGAL), a small iron-transporting protein largely produced by kidney tubular cells following cellular damage^27^. In line with these observations, we noticed an increase in *LCN2* mRNA and protein expression in MMA compared to control cells (Fig. 2g; Supplementary Fig. 1i). The link between compromised mitochondria, oxidative stress and LCN2 overproduction was substantiated by loss-of-function interventions in primary proximal tubule (PT) cells derived from wild-type mouse kidneys^28^. Short-hairpin (sh) RNA-induced knockdown of *Atg7* encoding an essential protein necessary for autophagy causes mitochondrial dysfunction and mitochondrial oxidative stress with increased production of Lcn2 in these cells (Supplementary Fig. 2a–e). These data indicate a functional link between mitochondrial dysfunction, oxidative stress and LCN2 overproduction, hence the existence of mitochondria-derived epithelial stress in kidney tubular cells.

### *MMUT*-deficient phenotypes in model organisms

To explore the consequences of the *MMUT* deficiency in vivo, we investigated a *Mmut* ^KO/KI^ mouse model carrying a mutant *Mmut* allele (p.Met698Lys, corresponding to the patient mutation p.Met700Lys) and a knockout *Mmut* allele^29^. The loss of Mmut was reflected by the accumulation of MMA (Supplementary Fig. 3a) and distorted kidney mitochondria which appear rod-like shape with impaired cristae organization (Supplementary Fig. 3b). Consistent with morphologically aberrant mitochondria, the membrane potential (Δψ_m_) was drastically reduced in MMA-accumulating kidney PT cells derived from *Mmut*^KO/KI^ mice (TMRM staining; Supplementary Fig. 3c). These changes were paralleled by a major mitochondrial oxidative stress, as testified by elevated mt-ROS levels (MitoSOX staining; Supplementary Fig. 3d). Despite the metabolic and/or mitochondrial alterations (Supplementary Fig. 3a, d), *Mmut*^KO/KI^ mice displayed mildly increased levels of urea (Supplementary Fig. 3e); no significant changes in kidney function (plasma levels and clearance of creatinine; Supplementary Fig. 3f, g); no changes in Lcn2 levels in kidneys as well as in plasma and/or urine—even in aged mutant mice (Supplementary Fig. 3h–j); and no structural damage (Supplementary Fig. 3k) nor interstitial inflammation (Supplementary Fig. 3l, m) in the kidneys compared to control littermates.

**Fig. 3:**
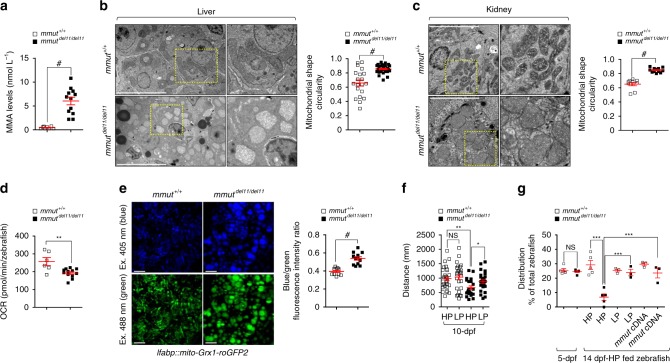
Mitochondrial abnormalities and phenotypic changes in *mmut-*deficient zebrafish. **a** Quantification of MMA levels by LC-MS/MS; *n* = 8 *mmut*^+/+^ and *n* = 12 *mmut*^del11/del11^ zebrafish larvae. **b**, **c** Representative images and quantification of the mitochondrial shape (expressed as circularity) in (**b**) livers and in (**c**) kidneys of 10-dpf *mmut* zebrafish (*n* ≥ 9 and *n* ≥ 10 randomly selected and non-overlapping fields of views for zebrafish livers and kidneys, respectively). The whole-field images are pooled from three distinct zebrafish kidneys and livers, respectively. Dotted yellow squares contain images at higher magnification. **d** Oxygen consumption rate (OCR) and individual parameters for basal respiration in 10-dpf-*mmut* zebrafish, *n* = 6 *mmut*^+/+^ and 12 *mmut*^del11/del11^ zebrafish larvae. **e** Zebrafish expressing mito-Grx1-roGFP2 in the liver were outcrossed with *mmut*^+/del11^ zebrafish. Representative images and quantification of the ratio between 405 (blue) and 488 (green) fluorescence intensities, with each point representing the average blue/green fluorescence intensity ratio in a zebrafish liver; *n* = 11 *mmut*^+/+^ and *n* = 12 *mmut*^del11/del11^ zebrafish larvae. **f** Tracking analyses of motor behaviour in 10-dpf*-mmut* zebrafish fed with a high- or low-protein diet (HP or LP, respectively). Quantification of the distance, with each point representing the average distance covered by an individual zebrafish; *n* = 28 HP-fed *mmut*^+/+^, *n* = 26 LP-fed *mmut*^+/+^, *n* = 23 HP-fed *mmut*^del11/del11^ and *n* = 27 LP-fed *mmut*^del11/del11^ zebrafish larvae. **g** Distribution of *mmut* zebrafish (expressed as the percentage of the total zebrafish larvae) in 5-dpf or in 14-dpf zebrafish fed with either HP or LP diet  or in *mmut* zebrafish stably expressing *mmut* in the liver; *n* ≥ 3 biologically independent experiments, with each containing ~100 *mmut* zebrafish larvae. Plots represent mean ± SEM. Two-tailed Student’s *t*-test, *P < 0.05, **P < 0.01 and ^#^*P* < 0.0001 relative to *mmut*^+/+^ or HP-*mmut*^del11/del11^ in **a**–**f**. One-way ANOVA followed by Bonferroni’s post hoc test, ***P < 0.001 relative to *mmut*^+/+^ or 14-dpf-HP-fed *mmut*^+/+^ or to 14-dpf-HP-fed *mmut*^del11/del11^ zebrafish larvae in **g**. Scale bars are 5 μm in **b** and **c**, and 100 μm in **e**. NS non-significant. Source data are provided as a Source Data file.

As the *Mmut*^KO/KI^ mouse model does not show key features of kidney disease in MMA, we established the first *mmut*-knockout zebrafish model using CRISPR/Cas9 genome editing. We obtained a zebrafish mutant line carrying an 11-bp-CRISPR/Cas9-induced deletion (*mmut*^del11/de111^), generating a premature stop codon within exon 3, resulting in a truncated protein deprived of its catalytic activity (Supplementary Fig. 4a‒c). Homozygous *mmut*^del11/del11^ zebrafish larvae, which appear morphologically normal and display no obvious development defects (Supplementary Fig. 4d), exhibit accumulation of MMA (Fig. 3a), which was abolished by re-expressing wild-type *mmut* cDNA in the liver (Supplementary Fig. 4e), validating the specificity of the deletion model. When compared to control littermates, both the kidney and the liver of *mmut-*deficient zebrafish exhibited altered mitochondrial morphology characterized by increased mitochondrial circularity (Fig. 3b, c) with perturbed cristae organization. Seahorse metabolic flux analyses revealed impaired mitochondrial bioenergetics in *mmut*-deficient zebrafish when compared to control larvae (Fig. 3d). These changes were paralleled by a major mitochondrial oxidative stress, as testified by in vivo imaging and ratiometric light-sheet microscopy-based analyses of glutathione redox fluorescent signals in liver Grx1-roGFP2-labelled mitochondria (Fig. 3e), demonstrating the evolutionary conservation of this connection. Furthermore, *mmut*-deficient zebrafish larvae markedly swim over shorter distances (Fig. 3f; Supplementary Fig. 3f) and show an excessive mortality (Fig. 3g) compared to control larvae. Both traits were rescued by feeding *mmut*-deficient zebrafish larvae (Fig. 3f, g) with low-protein diet—a strategy used in the MMA management care^12^. Re-expressing wild-type *mmut* cDNA in the liver of *mmut*-deficient zebrafish larvae, which normalized the levels of MMA metabolite and blunted the excessive mortality (Fig. 3g; Supplementary Fig. 4e), did not rescue the abnormal swimming phenotype (Supplementary Fig. 4g). Collectively, these results demonstrate that *MMUT* deficiency compromises the function and the homeostasis of mitochondrial network, both in vitro and in vivo.

### *MMUT* deficiency induces autophagy

**Fig. 4:**
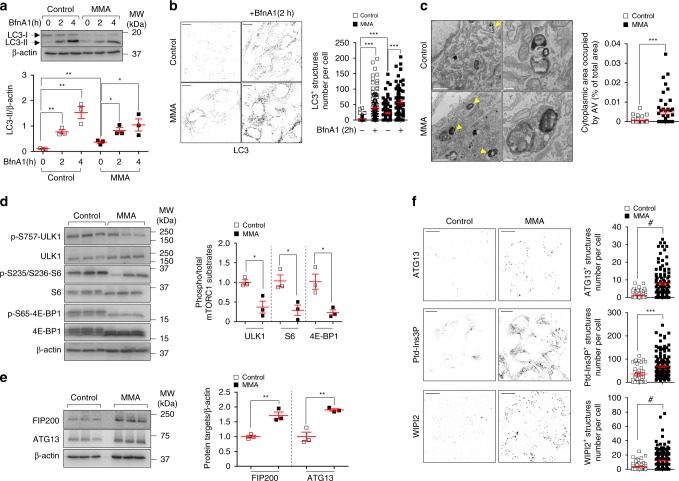
*MMUT* deficiency stimulates autophagy in MMA kidney cells. **a**, **b** Cultured cells were exposed to lysosome-based proteolysis inhibitor Bafilomycin A1 (BfnA1, 250 nM for the indicated times). Representative immunoblotting and quantification of LC3-II; *n* = 3 biologically independent experiments. Two-tailed Student’s *t*-test, *P < 0.05 and **P < 0.01 relative to untreated control or MMA cells. **b** Representative inverted images and quantification of numbers of punctate LC3^+^ structures per cell; *n* = 122 untreated control cells, *n* = 127 BfnA1-treated control cells, *n* = 127 untreated MMA cells and *n* = 77 BfnA1-treated MMA cells. Values are pooled from three biologically independent experiments. One-way ANOVA followed by Bonferroni’s post hoc test, ***P < 0.001 relative to untreated control or MMA cells. **c** Representative electron micrographs and quantification of cytoplasm area occupied by autophagy vacuoles (AV; expressed as the percentage of the total area); *n* = 43 control cells and *n* = 45 MMA cells pooled from two biologically independent experiments. Arrowheads indicate EM-compatible AV. **d**, **e** Immunoblotting and quantification of (**d**) phosphorylated and total forms of mTORC1 substrates and of (**e**) FIP200 and ATG13, *n* = 3 biologically independent experiments. **f** Representative inverted images and quantification of numbers of ATG13^+^ (top) or Ptd-Ins3P^+^ (middle) or WIPI2^+^ (bottom) structures per cell, respectively. Number of ATG13^+^ structures: *n* = 86 control cells and *n* = 118 MMA cells. Number of Ptd-Ins3P^+ ^structures: *n* = 41 control cells and *n* = 141 MMA cells. Number of WIPI2^+^ structures: *n* = 71 control cells and *n* = 253 MMA cells. Values are pooled from three biologically independent experiments. Plots represent mean ± SEM. Two-tailed Student’s *t*-test, *P < 0.05, **P < 0.01, ***P < 0.001 and ^#^*P* < 0.0001 relative to control cells in **c**–**f**. β-actin was used as a loading control in **a**, **d** and **e**. Scale bars are 10 μm in **b** and **f**, and 1 μm and 250 nm in **c** (left and right panels, respectively). NS non-significant. Source data are provided as a Source Data file.

As damaged mitochondria are normally removed by autophagy–lysosome pathways^30^, we hypothesized that mitochondrial abnormalities in MMA cells might reflect changes in autophagy–lysosome degradation systems. We measured autophagy by detecting the conversion of the non-lipidated form of LC3-I to the lipidated, autophagosome-associated form LC3-II through immunoblotting and/or by quantifying the numbers of punctate LC3^+^ vesicles through confocal microscopy and/or the abundance of electron microscopy (EM) structures compatible with autophagic vacuoles (AVs)^31^. Compared to control cells, we detected in MMA cells an elevated conversion of LC3-I to LC3-II (Fig. 4a) and higher numbers of punctate LC3-positive structures (Fig. 4b), and more EM structures compatible with AVs (Fig. 4c), whose nature was confirmed by correlated light electron microscopy (CLEM; Supplementary Fig. 5a).

An increased number of AVs might arise from the stimulation of autophagosome biogenesis or from alteration of their degradation by lysosomes. To distinguish between these two possibilities, we treated at two different time points (2h and 4 h) control and MMA cells with Bafilomycin A1 (BfnA1), a lysosome–proteolysis inhibitor that blocks the cellular degradation of autophagosomes that subsequently accumulate. Treatment of MMA cells with BfnA1 further increased the already elevated steady levels of LC3-II and the numbers of punctate LC3^+^ structures at two different time points where any changes would reflect altered autophagosome biogenesis (Fig. 4a, b; ref. ^31^). These cellular alterations were not associated with changes in autophagosome trafficking (as measured by LC3/LAMP1-positive structures in cells in response to short incubations with non-saturating concentration of BfnA1; Supplementary Fig. 5b); nor in autophagosome–lysosome fusion, as testified by augmented protein levels of Rab7—a small GTPase protein that regulates autophagosome–lysosome fusion^31^ (Supplementary Fig. 5c); nor in lysosome dynamics, as scored by the abundance of lysosome-associated protein LAMP1 and cathepsin-D (Supplementary Fig. 5c); nor in lysosome-based degradative capacity, as monitored by Bodipy-FL-Pepstatin A, a fluorescence-tagged probe that binds to the active site of cathepsin-D in acidic lysosomes (Supplementary Fig. 5d), implying that the *MMUT* deficiency stimulates autophagosome biogenesis rather than slowing down their degradation.

The connection between *MMUT* deficiency and induction of autophagy was substantiated by the increased formation of SQSTM1/p62^+^ aggregates containing polyubiquitinated proteins in MMA cells, despite unchanged levels of *SQSTM1* and unmodified activity of the proteasome (Supplementary Fig. 5e, f). These changes were rescued by treating the MMA cells with the mitochondrially targeted ROS scavenger mito-TEMPO (MT; 10 μM for 24 h; Supplementary Fig. 5f), in line with recent observations that increased levels of SQSTM1/p62 might enable a more efficient autophagy to maintain cellular homeostasis during oxidative stress^32,33^. Similar findings were observed in human kidney: LC3-marked autophagy vesicles and SQSTM1^+^ aggregates remarkably accumulated in kidney tubules from a patient with MMA (Supplementary Fig. 5g).

Furthermore, the upstream signalling cascade regulating autophagy, such as mTORC1 complex, was markedly reduced in MMA compared to control cells (Fig. 4d). This was paralleled by activation of the ULK1 complex—the most upstream autophagy machinery controlling autophagosome biogenesis, as indicated by increased protein levels of ULK1 complex subunits FIP200 and Atg13 (Fig. 4e) and numbers of initiation foci containing ULK1 complex subunit Atg13 (Fig. 4f, top panel). In turn, these changes were reflected by the heightened production of the autophagy-relevant pool of Ptd-Ins3P (Fig. 4f, middle panel), triggering the recruitment of downstream autophagy effector WIPI2 that stimulates the biogenesis of autophagosomes (Fig. 4f, bottom panel). Conversely, exposing MMA cells to the class III phosphoinositide 3-kinase (PI3K) vacuolar protein sorting 34 (Vps34) kinase inhibitor SAR405, which blocks the production of Ptd-Ins3P^34^, prevented the LC3-I-to-LC3-II conversion, hence the formation of autophagosomes induced by the *MMUT* deficiency (Supplementary Fig. 6a, b). Collectively, these data suggest that *MMUT* deficiency stimulates autophagy by regulating, at least in part, upstream signalling cascades that regulate autophagosome biogenesis.

### *MMUT* deficiency impairs degradation of damaged mitochondria

**Fig. 5:**
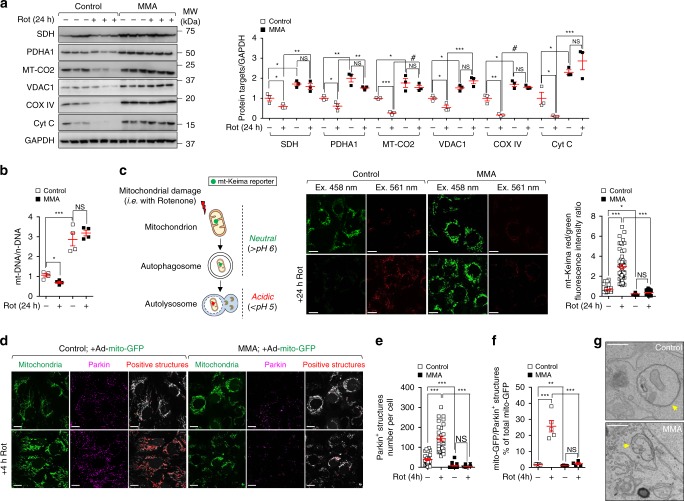
Impaired mitophagy-mediated degradation of MMA diseased mitochondria. **a**–**f** Cells were exposed to Rotenone (5 μM) for the indicated time. **a** Immunoblotting and quantification of indicated mitochondrial proteins; *n* = 3 biologically independent experiments. GAPDH was used as a loading control. **b** The ratio between mitochondrial (*ND1*) and nuclear DNA (*ACTB*) was determined by quantitative PCR; *n* = 4 biologically independent experiments. **c** Workflow of the strategy used to monitor the cellular delivery of damaged mitochondria to lysosomal compartments. Cells were transduced with adenoviral particles carrying the mitochondrially targeted form of Keima (mt-Keima) for 24 h. Representative images and quantification of ratio between red and green fluorescence intensities, with each point representing the average red/green fluorescence intensity ratio in a cell; *n* = 46 untreated control cells and *n* = 52 Rotenone-treated control cells, and *n* = 51 untreated MMA cells and *n* = 53 Rotenone-treated MMA cells. **d**‒**f** Cells were transduced with adenoviral particles bearing the mitochondrially targeted green fluorescent protein (Ad-mito-GFP). After 24 h post-transduction, the cells were exposed to Rotenone for 4 h, immunostained for Parkin (magenta) and analysed by confocal microscopy. **d** Representative images and quantification of number of (**e**) Parkin^+^ and (**f**) GFP/Parkin^+^ structures. Number of Parkin^+^ structures per cell: *n* = 30 untreated and Rotenone-treated control cells, *n* = 39 untreated MMA cells and *n* = 52 Rotenone-treated MMA cells. Number of GFP/Parkin^+^ structures (expressed as the percentage of total mitochondria): *n* = 5 randomly selected and non-overlapping fields of views per each condition. Each whole-field image contains at least 10 cells. The whole-field images are pooled from three biologically independent experiments. **g** Representative electron micrographs (EM) showing the engulfment of mitochondria within EM-compatible, double membraned-autophagic vacuoles in control but not in MMA cells. Values in **c** and **e** are pooled from three biologically independent experiment. Plots represent mean ± SEM. Two-tailed Student’s *t-*test, *P < 0.05, **P < 0.01, ***P < 0.001 and ^#^*P* < 0.0001 relative to untreated control or to Rotenone-treated control cells in **a**, **b** and **f**. One-way ANOVA followed by Bonferroni’s post hoc test, *P < 0.05 and ***P < 0.001 relative to untreated control or to Rotenone-treated control cells in **c** and **e**. Scale bars are 10 μm in **c** and **d**, and 250 nm in **g**. NS non-significant. Source data are provided as a Source Data file.

Considering the persistence in MMA cells of dysfunctional (ROS overproducing) mitochondria and high numbers of autophagic vesicles/autophagosomes, we reasoned that *MMUT* deficiency might sabotage the mitophagy-mediated demolition of MMA-damaged mitochondria. To verify our hypothesis, we treated both control and MMA cells with Rotenone to damage mitochondria and selectively activate their mitophagy-mediated degradation. After 24 h treatment with Rotenone, control cells showed a marked decrease in overall mitochondrial proteins (Fig. 5a) and in the ratio between mt-DNA and n-DNA (Fig. 5b), whereas both parameters were conversely retained in MMA cells. These changes were verified by measuring the overall mitochondrial proteins in cells cultured with other mitochondria-damaging compounds such as the electron transport chain inhibitors Oligomycin and Antimycin A (4 and 0.8 μM, respectively; Supplementary Fig. 7a). The depletion of MMUT did not alter the content of other cellular organelles producing ROS, such as peroxisomes, both under normal and autophagy/mitophagy-evoked conditions (Supplementary Fig. 7b), nor the dynamics and homeostasis of endolysosome system (Supplementary Fig. 5c, d).

As the loss of MMUT function results in a block of basal and stress-induced mitophagy, we hypothesized that *MMUT* deficiency might paralyse the delivery of MMA-damaged mitochondria to autophagy–lysosome degradation systems. To tackle this hypothesis, we utilized the ratiometric pH-sensitive imaging-based method^22,35^ to measure the delivery of dysfunctional mitochondria (which were labelled by the mitochondrially targeted form of a fluorescent Keima protein, mt-Keima) to lysosomes. When damaged mitochondria are delivered and engulfed within autolysosomes (e.g. mito-autolysosome), a spectral shift of mt-Keima occurs owing to the low pH (Fig. 5c). We validated mt-Keima as a bona fide reporter of mito-autolysosome formation in autophagy-deficient PT cells derived from the kidneys of *Atg7*^fl/fl^ mice (adenovirus-mediated, Cre-induced deletion of floxed *Atg7* alleles; Supplementary Fig. 7c; ref. ^36^), or from kidneys of *Pink1* or *Prkn2* (encoding Parkin) knockout (KO) mice (Supplementary Fig. 7e, f), and treated with Rotenone. Compared to controls cells, the deletion of *Atg7*, *Pink1*, or *Prkn2* was reflected by a loss of the expected shift of mt-Keima from a green mitochondrial to a red punctate appearance (Supplementary Fig. 7d, g) induced by Rotenone. Of note, basal mitophagy was reduced in autophagy (*Atg7*)-deficient PT cells (Supplementary Fig. 7d) whereas it was comparable in wild type and *Pink1* or *Prkn2* KO cells (Supplementary Fig. 7g), in line with recent reports suggesting that basal mitophagy might occur independently of Pink1 in mouse tissues of high metabolic demand including the kidneys^37^.

Next, we similarly expressed mt-Keima in both control and MMA cells (Supplementary Fig. 7h) and exposed the cells to Rotenone to follow the delivery of damaged mitochondria to lysosomes. After 24 h Rotenone treatment, control cells showed a substantial green-to-red fluorescent shift—indicative of delivery of damaged mitochondria to lysosomes, whereas this shift was abolished in MMA cells (Fig. 5c). Notably, under control conditions, MMA cells displayed lower steady-state mt-Keima red/green ratio values than controls, suggesting that *MMUT* deficiency compromises the delivery of damaged mitochondria to autolysosomes in both normal and stress-induced conditions (Fig. 5c).

### Mitophagy-mediated quality control in *COX10* deficiency

In order to test whether the altered mitophagy associated with the functional loss of MMUT is present in other mitochondrial diseases, we investigated the contribution of mitophagy-mediated removal and quality control systems in a model of impaired cytochrome oxidase assembly (*COX10* deficiency)—taken as a paradigm of primary mitochondrial disease^38^. We transduced PT cells derived from the kidneys of floxed *Cox10*^fl/fl^ mice with Cre-recombinase bearing adenoviral particles to conditionally inactivate the floxed *Cox10* alleles in vitro (Supplementary Fig. 8a, b). The deletion of *Cox10* was verified by reverse transcription-quantitative PCR (Supplementary Fig. 8c) and indirectly by immunoblotting for mitochondrial COX IV (Supplementary Fig. 8d; ref. ^39^). The *Cox10*-deleted cells showed significant mitochondrial alterations (e.g. decreased membrane mitochondrial potential and increased generation of mitochondrial ROS; Supplementary Fig. 8e, f). These alterations were not associated with major changes in mitochondrial content as indicated by comparable ratios of mt-DNA/n-DNA (Supplementary Fig. 8g) and by similar levels of mitochondrial proteins (Supplementary Fig. 8h) between control and *Cox10*-deleted PT cells. Notably, under normal conditions *Cox10*-deleted PT cells displayed higher steady-state mt-Keima red/green ratio values than control cells (Supplementary Fig. 8i), which were reflected by elevated transcript levels of mitophagy regulating gene *Pink1* (Supplementary Fig. 8j). Treatment with Rotenone induced in both control and *Cox10*-depleted PT cells the green-to-red fluorescent shift—indicative of delivery and engulfment of damaged mitochondria within autolysosomes (Supplementary Fig. 8i). Collectively, these data suggest that mitochondrial alterations encountered in *Cox10*-deficient kidney cells are not linked to anomalies in mitophagy-mediated degradation, in contrast to the defective mitochondrial homeostasis and mitophagy-mediated quality control caused by *MMUT* deficiency in patient-derived kidney tubular cells.

### *MMUT* deficiency skews PINK1/Parkin-mediated mitophagy

The PINK1/Parkin-induced mitophagy maintains the quality of the mitochondrial network by priming dysfunctional mitochondria for autophagy‒lysosome degradation pathways^40^. Therefore, we hypothesized that *MMUT* deficiency might compromise the PINK1/Parkin-mediated priming of MMA stressed mitochondria to autophagic–lysosomal degradation. Due to the lack of commercially available antibodies able to detect endogenous PINK1, we resorted to the translocation of Parkin to damaged mitochondria—a key downstream step following the activation of PINK1—as a bona fide reporter to assess the PINK1/Parkin-priming mechanisms^22,41,42^. We labelled the mitochondrial network by transducing both control and MMA cells with an adenovirus that expresses the mitochondrially targeted green fluorescent protein (Ad-mito-GFP). Twenty-four hours post-transduction, we exposed the mt-GFP-expressing cells to Rotenone (5 μM for 4 h) and scored the translocation of Parkin to mito-GFP-flagged mitochondria by confocal microscopy^42^. Using a validated α-Parkin antibody (see Supplementary Fig. 10e), we observed that Rotenone treatment expectedly heightened the numbers of Parkin^+^ clusters and the translocation of Parkin to mito-GFP^+^-damaged mitochondria in control cells (Fig. 5d‒f), indicating a proper PINK1/Parkin-mediated quality control. Conversely, MMA cells displayed a decrease in numbers of Parkin^+^ clusters and translocation of Parkin to damaged mitochondria at baseline and in Rotenone-evoked stress conditions (Fig. 5d‒f). These changes were complemented by the lack of engulfment of damaged mitochondria within EM-compatible autophagy vacuoles in MMA cells (Fig. 5g), supporting the concept of defective marking of diseased mitochondria for autophagy‒lysosome-based degradation.

**Fig. 6:**
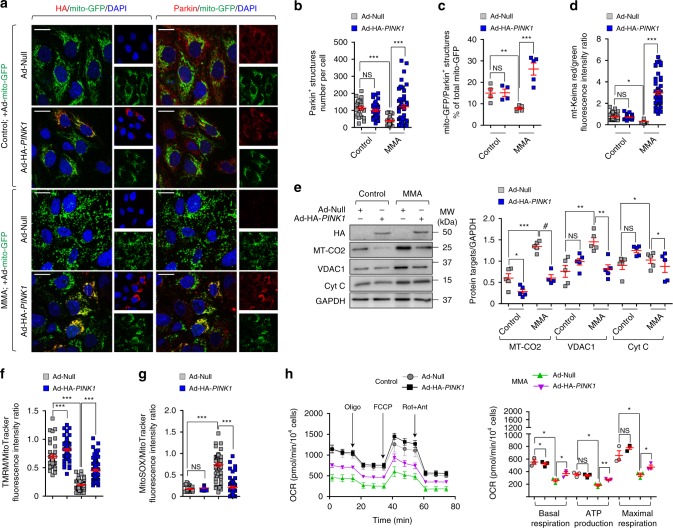
Rescue of mitochondrial function and homeostasis by re-expressing PINK1 in MMA kidney cells. **a**–**c** Cells were transduced with adenovirus particles expressing mitochondrially targeted GFP (Ad-mito-GFP, green) and with adenovirus particles bearing either Null or HA-*PINK1*. Cells were immunostained for HA (red) and Parkin (red). **a** Representative images and **b** quantification of numbers of Parkin^+^ structures in a cell. Number of control cells transduced with Null (*n* = 25) or HA-*PINK1* (*n* = 39) and MMA cells transduced with Null (*n* = 57) or HA*-PINK1* (*n* = 55). **c** Quantification of mito-GFP/Parkin^+^ structures (expressed as the percentage of total mitochondria); *n* *≥* 4 randomly selected and non-overlapping fields of views per each condition. Nuclei counterstained with DAPI (blue). **d**, **e** Null and HA-*PINK1*-expressing cells were transduced with adenovirus particles bearing mt-Keima. **d** Confocal microscopy-based quantification of the red/green fluorescence intensity ratio in a cell. Number of control cells transduced with Null (*n* = 46) or HA-*PINK1* (*n* = 38) and MMA cells transduced with Null (*n* = 51) or HA−*PINK1* (*n* = 54). **e** Representative immunoblotting and quantification of the indicated mitochondrial proteins; *n* = 5 biologically independent experiments. **f**, **g** Cells were loaded with (**f**) TMRM (green) or (**g**) MitoSOX (green) and MitoTracker (red), and analysed by confocal microscopy. Quantification of TMRM/MitoTracker or MitoSOX/MitoTracker fluorescence intensity ratio in a cell. Number of control cells transduced with Null (*n* = 43) or HA-*PINK1* (*n* = 62) and MMA cells transduced with Null (*n* = 83) or HA-*PINK1* (*n* = 84) for TMRM/MitoTracker. Number of control cells transduced with Null (*n* = 38) or HA-*PINK1* (*n* = 56) and MMA cells transduced with Null (*n* = 59) or HA-*PINK1* (*n* = 64) for MitoSOX/MitoTracker. **h** Oxygen consumption rate (OCR) and individual parameters for basal respiration, ATP production and maximal respiration. OCRs were measured at baseline and after the sequential addition of Oligomycin (Oligo, 1 μM), FCCP (0.5 μM) and Rotenone (Rot; 1 μM) + Antimycin A (Ant; 1 μM). Values in **a**–**h** are pooled from three biologically independent experiments. Plots represent mean ± SEM. One-way ANOVA followed by Bonferroni’s post hoc test, *P < 0.05 and ***P < 0.001 relative to control or MMA cells transduced with Null in **b**, **d**, **f** and **g**. Two-tailed Student’s *t*-test, *P < 0.05, **P < 0.01, ***P < 0.001 and ^#^*P* < 0.0001 relative to control and MMA cells transduced with Null in **c**, **e** and **h**. Scale bars,10 μm. NS non-significant. Source data are provided as a Source Data file.

The role of defective PINK1-mediated quality control and surveillance systems was further assessed by transducing MMA cells with an adenovirus that expresses human hemagglutinin (HA)-tagged PINK1 (Ad-HA-*PINK1*). The functional re-expression of PINK1 at mt-GFP-flagged mitochondria in MMA cells markedly increased the numbers of Parkin^+^ clusters and rescued the translocation of Parkin to MMA-damaged mitochondria (Fig. 6a‒c), inducing their delivery and degradation by autophagy‒lysosome systems, as indicated by mt-Keima reporter (Fig. 6d; Supplementary Fig. 9a) and analyses of mitochondrial proteins (Fig. 6e). In parallel, the functional  re-expression of PINK1 improved mitochondrial functions in MMA cells (Fig. 6f–h; Supplementary Fig. 9b, c), compared to cells transduced with empty vector.

### *Mmut* deletion damages mitochondria causing stress

**Fig. 7:**
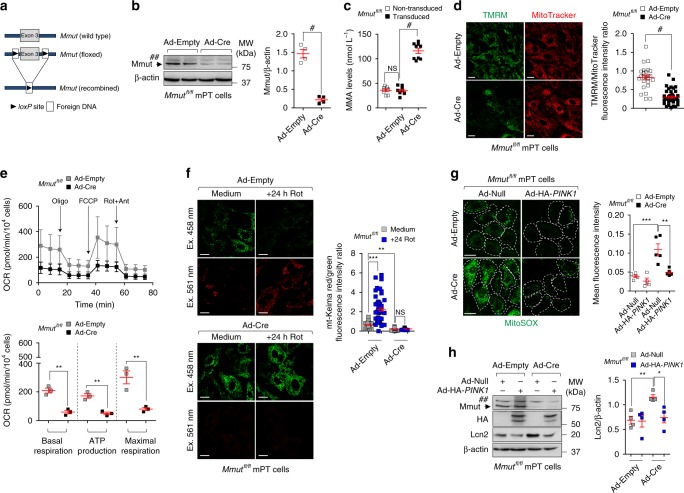
*MMUT* deletion damages mitochondria and blunts PINK1-directed mitophagy triggering epithelial stress in kidney cells. **a**–**i** Mouse proximal tubule (mPT) cells from floxed *Mmut* kidneys were transduced with adenovirus bearing Empty or Cre-recombinase for 5 days. **a** Workflow of strategy used to generate the floxed *Mmut* alleles. **b**, **c** Validation of *Mmut* deletion by **b** immunoblotting (*n* = 4 biologically independent experiments) and **c** by LC-MS/MS analysis of MMA levels (*n* = 9 replicates pooled from three biologically independent experiments). **d** Cells were loaded with TMRM (green) and MitoTracker (red). Confocal microscopy-based quantification of fluorescence intensity ratio in a cell. Number of cells transduced with Ad-Empty (*n* = 25) or Ad-Cre (*n* = 45). **e** Oxygen consumption rate (OCR) and individual parameters for basal respiration, ATP production and maximal respiration. OCRs were measured at baseline and after the sequential addition of Oligomycin (Oligo, 1 μM), FCCP (0.5 μM) and Rotenone (Rot, 1 μM) + Antimycin A (Ant, 1 μM). **f** *Mmut* cells were transduced with adenovirus expressing mitochondrially targeted form of Keima (mt-Keima) for 24 h and exposed to Rotenone (Rot, 5 μM for 24 h). Representative images and quantification of red/green fluorescence intensity ratio in a cell. Number of untreated (*n* = 68) and Rot-treated (*n* = 57) control cells, and number of untreated (*n* = 51) and Rot-treated (*n* = 58) *Mmut*-deleted cells. **g**, **h** Control and *Mmut*-deleted cells were transduced with adenovirus expressing Null or HA-*PINK1* for 24 h. **g** Cells were loaded with MitoSOX (green) and analysed by confocal microscopy. Representative images and quantification of MitoSOX fluorescence intensity, *n* ≥ 4 randomly selected and non-overlapping fields of views per condition, with each containing ~10 cells. **h** Representative immunoblotting and quantification of Lcn2, *n* = 4 biologically independent experiments. β-actin was used as a loading control. Values in **d**–**g** are pooled from three biologically independent experiments. Plots represent mean ± SEM. Asterisks denote non-specific bands in **b** and **h**. Two-tailed Student’s *t*-test, *P < 0.05, **P < 0.01, ***P < 0.01 and ^#^*P* < 0.0001 relative to control cells in **b**–**e** or relative to control or *Mmut*-deleted cells transduced with Ad-Null in **h**. One-way ANOVA followed by Bonferroni’s post hoc test, **P < 0.01 and ***P < 0.001 relative to untreated control cells or relative to control or *Mmut*-deleted cells transduced with Ad-Null in **f** and **g**. Scale bars,10 μm. NS non-significant. Source data are provided as a Source Data file.

To causally demonstrate the link between the loss of the enzyme MMUT, PINK1-directed mitophagy, mitochondrial dysfunctions and epithelial stress, we used gain and loss-of-function interventions in kidney cells carrying floxed *Mmut* alleles (*Mmut*^fl/fl^). The *Mmut*^fl/fl^ mice do not show any clinical phenotype, they have normal growth and display normal metabolite levels^43^. Primary PT cells derived from *Mmut*^fl/fl^ mouse kidneys were transduced with adenovirus particles bearing Cre-recombinase to conditionally delete *Mmut* in vitro (Fig. 7a, b).

The deletion of *Mmut* was reflected by augmented levels of MMA (Fig. 7c), and by reduced engulfment of damaged and/or dysfunctional mitochondria (e.g. decreased membrane potential and altered bioenergetics profiling; Fig. 7d, e) within autolysosomes (as scored by reduced mt-Keima red/green fluorescence ratio; Fig. 7f). Under these conditions, *Mmut*-deleted cells showed no significant increase in overall mitochondrial proteins compared to control cells (Supplementary Fig. 9d). In line with defective mitochondrial disposal, *Mmut*-deleted PT cells exhibited elevated mitochondrial oxidative stress (Fig. 7g) and cell damage (Lcn2 overproduction; Fig. 7h), which were abolished by restoring PINK1-directed mitophagy with an adenovirus expressing (HA)-tagged *PINK1* (Fig. 7g, h; Supplementary Fig. 9e).

Conversely, CRISPR/Cas9-mediated KO of *PINK1* or *PRKN2* (encoding Parkin) in human HAP-1 cells (Supplementary Fig. 10) led to mitochondrial alterations, i.e. decreased membrane potential (Supplementary Fig. 11a), reduced bioenergetics profiling and elevated mitochondrial ROS production (Supplementary Fig. 11b, c), which were similar to, albeit milder than, those encountered in patient-and-in *Mmut*-deleted cells. Notably, we did not detect any differences in mitochondrial morphology between *PINK1* and *PRKN2 *wild type and KO cells (Supplementary Fig. 11d). Taken together, these data suggest that the deficiency of *Mmut* impedes the PINK1-induced translocation of Parkin to MMA-damaged mitochondria, halting their delivery and subsequent degradation by autophagy‒lysosome systems. In turn, these cellular defects promote the accumulation of dysfunctional (ROS overproducing) mitochondria that ultimately trigger epithelial stress and kideny damage.

### Drug–disease network perturbations in MMA cells

**Fig. 8:**
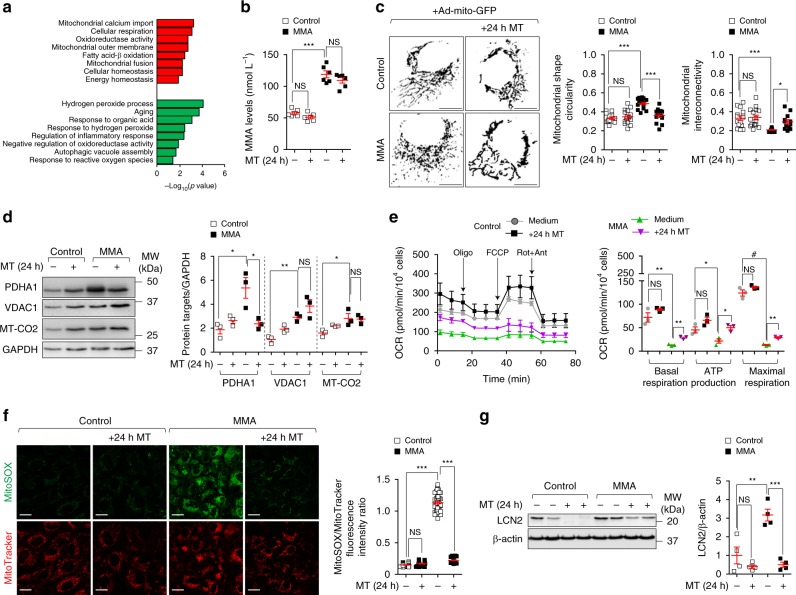
Mitochondria-targeted ROS scavenger mito-TEMPO repairs mitochondrial functions in MMA kidney cells. **a** Gene ontology (GO) annotations significantly upregulated (red) and downregulated (green) by the in silico-prioritized hits according to Drug Set Enrichment Analysis (DSEA). **b**–**g** Cells were treated in the presence or in the absence of mito-TEMPO (MT; 10 μM for 24 h). **b** Quantification of MMA levels by LC-MS/MS; *n* = 6 replicates. One-way ANOVA followed by Bonferroni’s post hoc test, ***P < 0.001 relative to untreated control or MMA cells. **c** Cells were transduced with adenovirus particles bearing the mitochondrially targeted GFP (Ad-mito-GFP, green). After 24 h post-transduction, the cells were treated with MT and analysed by confocal microscopy. Representative inverted images and quantification of mitochondrial circularity or interconnectivity. Circularity: *n* = 15 cells per each condition. Interconnectivity: *n* = 15 untreated and MT-treated control cells, *n* = 15 and *n* = 13 untreated and MT-treated MMA cells, respectively. One-way ANOVA followed by Bonferroni’s post hoc test, *P < 0.05 and ***P < 0.001 relative to untreated control or MMA cells. **d** Representative immunoblotting and quantification of the indicated mitochondrial proteins. GAPDH was used as a loading control. **e** Oxygen consumption rate (OCR) and individual parameters for basal respiration, ATP production and maximal respiration. OCRs were measured at baseline and after the sequential addition of Oligomycin (Oligo, 1 μM), FCCP (0.5 μM) and Rotenone (Rot, 1 μM) + Antimycin A (Ant, 1 μM). **f** Cells were loaded with MitoSOX (green, 2.5 μM for 30 min at 37 °C) and MitoTracker (red; 1 μM for 30 min at 37 °C), and analysed by confocal microscopy. Representative images and quantification of mitochondrial ROS (calculated as the ratio between MitoSOX and MitoTracker fluorescence intensities; each point representing the average fluorescence intensity ratio in a cell). Number of untreated (*n* = 36) or MT-treated (*n* = 28) control cells and number of untreated (*n* = 51) or MT-treated (*n* = 28) MMA cells. One-way ANOVA followed by Bonferroni’s post hoc test, ***P < 0.001 relative to untreated control or MMA cells. **g** Representative immunoblotting and quantification of LCN2, *n* = 4 biologically independent experiments. β-actin was used as a loading control. Plots represent mean ± SEM. Values in **b**–**f** are pooled from three biologically independent experiments. Two-tailed Student’s *t-*test, *P < 0.05, **P < 0.01, ***P < 0.001 and ^#^*P* < 0.0001 relative to untreated control or MMA cells in **d**, **e** and **g**. Scale bars^,^ 10 μm. NS non-significant. Source data are provided as a Source Data file.

We used a drug–disease network-based computational modelling approach (Mantra 2.0; Mode of Action by Network Analysis; http://mantra.tigem.it; ref. ^44^) to identify potential druggable pathways to overcome cellular dysfunctions associated with MMA. Mantra elucidates genome-wide targetable candidates by systematically matching disease gene signature (Supplementary Fig. 12a), here derived from the comparison of expression profiles between MMA and their related control cells (Supplementary Fig. 12b, c; Supplementary Data 1), against a library of 1309 small bioactive drug compounds. Predictions for drug–disease pairs were based on the hypothesis that if a drug reverses the disease gene signature, that drug might potentially target disease-relevant biological pathways^44^. Based on this assumption, the top-35 drug compounds transcriptionally closer to the inverse disease gene profile were identified. To determine the targeted-biological pathways in MMA, we transcriptionally phenotyped the top-35 drug compounds by performing Drug Set Enrichment Analysis (DSEA^45^)—a tool identifying, from transcriptional responses, the molecular pathways that are significantly modulated by drug compounds in a set. We run DSEA using as pathway databases Gene Ontology terms: biological process, molecular function and cellular component. Interestingly, DSEA revealed that the top-scored drug compounds modulate redox homeostasis-related pathways (Fig. 8a), in line with the mitochondrial oxidative stress encountered in MMA cells and with recent mouse transcriptomic profiling denoting a chronic activation of stress-related pathways in a transgenic mouse model expressing *Mmut* in the muscle^26^. It also yielded candidates affecting pathways that regulate mitochondrial homeostasis and functioning such as calcium import, fatty acid β-oxidation and cellular respiration; or pathways that control mitochondrial dynamics such as fusion; or pathways that modulate cellular responses to mitochondrial stress (Fig. 8a). These in silico analyses strongly support that mitochondria-targeting strategies might potentially reverse disease phenotype in MMA cells.

### Mitochondrial targeting in MMA cells and animal models

Inspired by the biological evidence and the MANTRA analysis, we tested whether mitochondria-targeted interventions might correct phenotypes in MMA. Control and MMA cells were cultured in the presence and in the absence of a mitochondria-targeted antioxidant mito-TEMPO (MT, 10 μM for 24 h), which rescues mitochondrial-based cell dysfunctions associated with the lysosome storage disease cystinosis^46^. Despite unchanged levels of MMA metabolite (Fig. 8b), treatment with MT effectively recovered mitochondrial morphology (as scored by the presence of curvilinear or elongated mitochondria; Fig. 8c) and partially the homeostasis of mitochondrial network (as testified by immunoblotting analyses for mitochondrial proteins; Fig. 8d), improved mitochondrial functionality and bioenergetics (Fig. 8e), lowered the formation of SQSTM1^+^ aggregates containing polyubiquitinated proteins (Supplementary Fig. 5f), and neutralized mitochondrial oxidative stress (Fig. 8f) and LCN2 overproduction (Fig. 8g) in MMA cells.

**Fig. 9:**
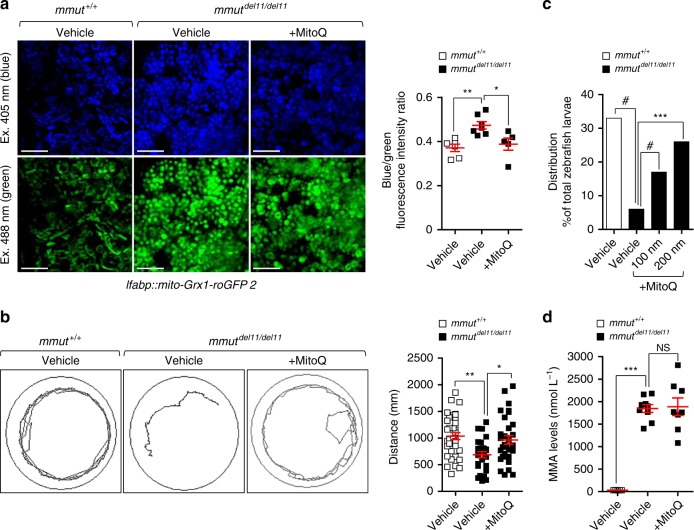
Mitochondria-targeted ROS scavenger MitoQ rescues the MMA–associated phenotypes in *mmut*–deficient zebrafish. **a**–**d** Zebrafish larvae were treated with vehicle or with the mitochondria-targeted ROS scavenger MitoQ (100 nM or 200 nM, respectively, for 24 h). **a** Representative images and quantification of ratio between 405 (blue) and 488 (green) fluorescence intensities in *mmut* zebrafish expressing mito-Grx1-roGFP2 in the liver. Each point represents the average fluorescence intensity ratio in an individual zebrafish liver; *n* = 6 vehicle-treated *mmut*^+/+^ zebrafish and *n* = 7 vehicle-treated *mmut*^del11/del11^ zebrafish, and *n* = 5 MitoQ-treated *mmut*^del11/del11^ zebrafish larvae. Kruskal‒Wallis followed by Dunn’s multiple comparison test, *P < 0.05 and **P < 0.01 relative to vehicle-treated *mmut*^+/+^ or vehicle-treated *mmut*^del11/del11^ zebrafish larvae. **b** Tracking analyses of motor behaviour in 10-dpf-*mmut* zebrafish larvae. Quantification of distance, with each point representing the average distance covered by an individual zebrafish; *n* = 28 vehicle-treated *mmut*^+/+^, *n* = 27 vehicle-treated *mmut*^del11/del11^ and *n* = 31 MitoQ-treated *mmut*^del11/del11^ zebrafish larvae. One-way ANOVA followed by Bonferroni’s post hoc test, *P < 0.05 and **P < 0.01 relative to vehicle-treated *mmut*^+/+^ or to vehicle-treated *mmut*^del11/del11^ zebrafish larvae. **c** Distribution of *mmut* zebrafish larvae (expressed as the percentage of total larvae) after treatment with either vehicle or MitoQ. Values are from one biological repeat, with *n* ≥ 77*mmut* zebrafish larvae per each group/condition. Chi-square goodness of fit test, ***P < 0.001 and ^#^*P* < 0.0001 relative to vehicle-treated *mmut*^*+/+*^ or *mmut*^del11/del11^ zebrafish larvae. **d** Quantification of MMA levels by LC-MS/MS; *n* = 5 vehicle-treated *mmut*^+/+^, *n* = 9 vehicle-treated *mmut*^del11/del11^ and *n* = 8 MitoQ-treated *mmut*^del11/del11^ zebrafish larvae. Plots represent mean ± SEM. Kruskal–Wallis followed by Dunn’s multiple comparison test, **P < 0.01 relative to vehicle-treated *mmut*^+/+^ or vehicle-treated *mmut*^del11/del11^ zebrafish larvae. Scale bars, 100 μm. NS non-significant. Source data are provided as a Source Data file.

To explore the translational potential of these findings, we treated *mmut*-deficient zebrafish larvae with low, non-toxic doses of the mitochondrially targeted antioxidant MitoQ (200 nM for 24 h). Treatment with MitoQ effectively reduced mitochondrial oxidative stress (Fig. 9a), improved the behavioural phenotype (Fig. 9b) and reduced the excessive mortality observed in *mmut*–deficient zebrafish larvae (Fig. 9c), in the absence of any significant changes in the MMA levels (Fig. 9d). Taken together, these findings suggest that repairing mitochondrial function might serve as an attractive therapeutic strategy for treating MMA.

## Discussion

The proper functioning of mitochondria is crucial for the homeostasis of specialized cell types, for instance those requiring high ATP levels for reabsorptive/transport activities^23,47–49^. Inherited defects in mitochondrial-localized proteins and/or enzymes, as exemplified by MMA, drive the accumulation of potentially toxic metabolites within mitochondrial matrix, promoting ultrastructural and/or functional alterations that ultimately cause life-threatening metabolic complications and organ dysfunction^10–13^. Here, combining gain- and-loss-of function interventions, both in vitro and in vivo, we decipher the link between mitochondrial abnormalities induced by *MMUT* deficiency and anomalies in PINK1/Parkin-mediated quality control and surveillance systems, triggering a level of mitochondrial dysfunction that drives epithelial stress and kidney damage in MMA.

**Fig. 10:**
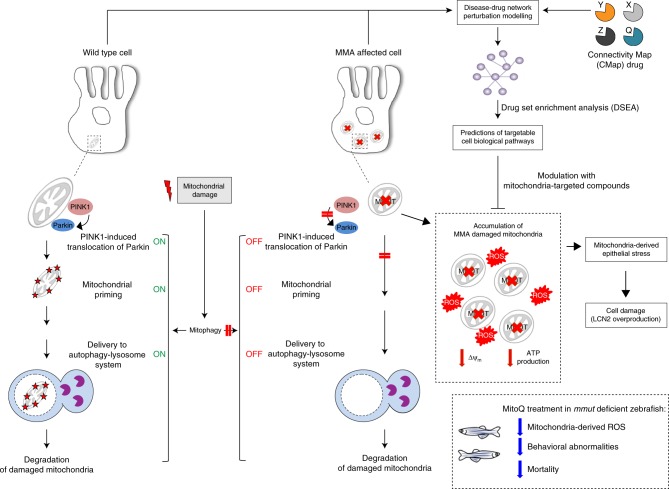
Proposed model depicting the link between mitochondrial dysfunctions and epithelial stress in MMA. In wild-type kidney cells (left), mitochondrial stress (e.g. treatment with Rotenone) stimulates PINK1-induced translocation of Parkin to damaged mitochondria. This triggers mitophagy and the subsequent disposal of dysfunctional mitochondria through autophagy–lysosome degradation systems, thereby safeguarding the homeostasis and function of the mitochondrial network. By contrast, in MMA-affected kidney cells (right), the impaired PINK/Parkin-mediated mitophagy impedes the delivery of damaged mitochondria and their degradation by autophagy‒lysosome pathways. This leads to accumulation of MMA-diseased mitochondria and exacerbates the mitochondrial alterations induced by *MMUT* deficiency, including accumulation of toxic metabolites, collapsed mitochondrial membrane potential (Δψ_m_), and abnormal energetic profiling and increased mitochondrial ROS. These mitochondrial alterations generate epithelial stress, causing ultimately cell damage (e.g. LCN2 overproduction). Drug–disease network perturbation modelling, based on transcriptome-wide profiles from MMA patient-derived kidney cells against a large compendium of gene signatures derived from 1309 small bioactive drug compounds, identifies targetable disease-relevant cell biological pathways. The modulation of the identified targets (e.g. treatment with mitochondria-targeted ROS scavengers MT or MitoQ) repairs mitochondrial dysfunctions, neutralizes epithelial stress and cell damage in MMA cells, and improves disease-relevant phenotypes in *mmut*–deficient zebrafish.

Kidney tubular cells derived from MMA patients and zebrafish lacking the enzyme MMUT exhibit mitochondrial fragmentation and lower membrane potential, impaired respiration and ATP production, and heightened mitochondrial ROS, generating epithelial stress and cell damage (Fig. 10). In particular, *MMUT* deficiency disables the PINK/Parkin-mediated mitophagy, leading to the accumulation of MMA-damaged and/or dysfunctional mitochondria that trigger cellular stress. Unbiased drug–disease network perturbation modelling predicted targetable biological processes including redox homeostasis whose modulation repairs mitochondria in patient-derived cells and alleviates disease-relevant phenotypes in a zebrafish model of MMA. These findings reveal the importance of mitophagy-mediated organelle quality control systems in safeguarding the functioning and homeostasis of the mitochondrial network, and offer potential therapeutic strategies for repairing mitochondrial dysfunctions in MMA and in other mitochondrial-related human diseases.

The epithelial cells that form the kidney tubules are enriched in mitochondria to sustain their specialized transport functions and integrity^23^. Defects that impair mitochondrial protein homeostasis or assembly might drastically lead to kidney tubule dysfunction in various types of diseases^50^. MMA patient-derived kidney cells show fragmented mitochondria and a marked increase in the abundance of mitochondrial DNA and resident mitochondrial proteins, suggesting that *MMUT* deficiency alters the homeostasis of the mitochondrial network. Furthermore, MMA cells display decreased mitochondrial membrane potential and reduced mitochondrial bioenergetics. These dysfunctions result in the generation of a large amount of oxygen radicals and cellular stress, which are observed in other mitochondrial diseases^51^. A similar accumulation of morphologically aberrant and dysfunctional mitochondria is observed in the kidney tubule cells of *Mmut*^KO/KI^ mice, demonstrating the key role of MMUT function for the mitochondrial network homeostasis and function. However, despite the metabolic and mitochondrial abnormalities, the *Mmut*^KO/KI^ mice develop no structural changes nor significant kidney failure, thus failing to recapitulate the kidney disease associated with MMA.

To overcome this difficulty, we established the first *mmut*-knockout zebrafish model using CRISPR/Cas9 genome editing. The patterning of the kidney tubule is remarkably conserved in the zebrafish pronephros versus mammalian kidney, including junctional complexes, endolysosomal apparatus and receptors and transporters^52^. Deficiencies in genes encoding receptors, enzymes and transporter that cause tubular dysfunction in humans have been shown to trigger similar pathological changes in zebrafish^46,53–55^. Further characteristics including high fecundity, unrivalled optical transparency and the possibility of housing in multi-well plates offer unique opportunities to perform (high-throughput) phenotypic screens in an in vivo context^56^.

Analogous to the metabolic and mitochondrial alterations encountered in *Mmut*^KO/KI^ and patient-derived kidney cells, the *mmut*–deficient zebrafish exhibit altered mitochondrial morphology and excessive mitochondrial damage, with an exaggerated mitochondrial oxidative stress and markedly decreased mitochondrial bioenergetics flux rates when compared to their control littermates. The demonstration of this conserved connection will require a more comprehensive elucidation of similarities and differences between zebrafish and human mitochondrial biology. The *mmut*-deficient zebrafish showed an MMA disease-relevant phenotype, including liver/kidney mitochondriopathy, impairment of the behavioural phenotypes and an excess of mortality. Of note, these two features are rescued by feeding mutant zebrafish with a low-protein diet—a strategy used in MMA patients as it prevents the accumulation of methylmalonic acid^14^. Intriguingly, our findings revealed that restoring *mmut* activity in the liver, which normalizes the levels of methylmalonic acid metabolite and blunts the excessive mortality, does not protect the *mmut*-deficient zebrafish from the abnormal swimming phenotype. The latter observation suggests that *mmut*-induced mitotoxicity in other cell types and organs (e.g. central nervous system, optic nerve and/or muscle) might govern the phenotypes encountered in *mmut*-deficient zebrafish.

The concept that mitochondrial dysfunctions and uncontrolled cellular stress might contribute to the MMA disease is in line with the observed correlation between mitochondrial dysfunction, oxidative stress and circulating LCN2 in a cohort of patients with MMA^10,26^. LCN2 is a secreted iron-transporting protein produced by kidney tubules following cellular damage; it is associated with kidney disease progression^27^ and metabolic disease^57^. Our studies performed on aged *Mmut*^KO/KI^ mice did not show any significant increase in the levels of Lcn2, in plasma, urine and kidney. The difference could reflect specific compensatory mechanisms^10,26^, or a specific time-course of induction in *Mmut*^KO/KI^ mice^29^.

A central question is how the loss of the enzyme MMUT disrupts mitochondrial network homeostasis and its function. The first line of defense to cope with mitochondrial damage is represented by the cellular quality control system^58^. The latter involves the degradation of dysfunctional mitochondria through an evolutionary conserved, catabolic self-eating process called macroautophagy (hereafter autophagy; ref. ^58^) and the renewal of components through biogenesis^20^. Normally, these homoeostatic processes suffice to clean the cells of damaged organelles.

We first noted that *MMUT* deficiency promotes autophagy. Indeed, *MMUT*-deficient cells show a heightened conversion of LC3-I to LC3-II, with increased autophagosome-associated LC3^+^ puncta as well as CLEM and/or EM structures compatible with AVs. *MMUT* deficiency may affect autophagy either by stimulating autophagosome biogenesis or by inhibiting the fusion between autophagosomes and lysosomes or by controlling the degradative function of lysosomes. These last hypotheses do not sound plausible in view of the similar coalescence of LAMP1-positive and LC3-positive vesicles and the unchanged lysosomal dynamics and lysosomal-based cellular degradation under *MMUT*-depleting conditions. Also, treatment with BfnA1 further increased the already elevated steady levels of LC3-II and the numbers of punctate LC3^+^ structures at two different time points where any changes would reflect altered autophagy biogenesis. Furthermore, downstream events regulating the degradation of autophagosomes remained unchanged. Several mechanisms may account for the stimulation of autophagy in *MMUT*-deficient cells: (1) reduced mTORC1 signalling, whose activity downregulates autophagy; (2) increased levels of ULK1 complex subunits FIP200 and Atg13 and numbers of initiation foci containing ULK1 complex subunit Atg13, whose activation regulates autophagy machinery involved in autophagosome formation; (3) augmented abundance of autophagy-relevant pool of Ptd-Ins3P, which recruits downstream autophagy effector WIPI2, hence stimulating autophagosome biogenesis; and (4) redox-dependent formation of intracellular SQSTM1/p62^+^ aggregates containing polyubiquitinated proteins, whose accumulation might activate autophagy^32^. Thus, deficiency of *MMUT* may stimulates autophagy by regulating upstream signalling cascades that regulate autophagosome biogenesis, in line with recent studies stating elevated autophagy markers in mitochondria-related diseases^59^ and dysregulation of autophagy–lysosome degradation pathways in tissue samples from patients with MMA^26^.

A growing body of evidence suggests that exhausted mitochondria are selectively targeted for autophagy by the PINK1/Parkin-dependent pathways^22^. PINK1 and Parkin also regulate mitochondrial quality control through other pathways including fusion/fission and biogenesis^60^. Mitochondrial damage activates the mitochondria-associated kinase PINK1, which recruits and activates Parkin’s E3 ubiquitin ligase activity, forming the basis of multiple signalling events that culminate in the engulfment of damaged mitochondria within lysosomes^40,41^. Considering the accumulation of MMA damaged and dysfunctional mitochondria and the stimulation of autophagy, we hypothesized that *MMUT* deficiency might compromise the PINK1/Parkin-mediated priming of MMA stressed mitochondria to autophagy–lysosome degradation systems. Indeed, our studies show that MMA cells (1) fail to clear dysfunctional mitochondria in both normal and mitophagy (e.g. treatment with Rotenone) induced conditions, as reflected by analyses of mitochondrial proteins and mitochondrial DNA; (2) fail to deliver damaged mitochondria to autophagy–lysosome degradation systems, as scored by the sensitive mt-Keima imaging-based assay; and (3) show decreased recruitment of Parkin to MMA mitochondria—a key downstream step following the activation of PINK1^22,40,41^. The dysfunction of the PINK1/Parkin-priming system impacts on the delivery and elimination of ROS-producing mitochondria through autophagy–lysosome degradation pathways, triggering epithelial stress in MMA. Accordingly, restoring mitophagy-mediated degradation through gain-of-function approaches targeting PINK1-mediated priming was sufficient to rescue the mitochondrial network, preserving homeostasis in MMA patient-derived kidney cells. Conversely, depleting *MMUT* in PT cells reduces the PINK1-mediated delivery and engulfment of mt-Keima tagged mitochondria within autophagy–lysosome degradation compartments, promoting mitochondrial stress and cell damage. Of note, when compared to control cells, *Mmut*-deleted PT cells showed no increase in mitochondrial proteins, contrasting with the situation observed in MMA patient-derived kidney cells. The difference could reflect effects of chronic mitotoxicity on mitochondrial clearance and quality control systems.

The genetic deletion of *PRKN2* (encoding Parkin) or *PINK1* in HAP-1 cells leads to mitochondrial alterations similar to, albeit milder than, those encountered in MMA patient derived- and in *Mmut*-deleted kidney cells. Thus, anomalies in PINK1/Parkin-mediated quality control might intersect the mitochondrial alterations induced by *MMUT* deficiency and contribute to the pathogenesis of MMA. The latter hypothesis is substantiated by our comparative studies of *Cox10* deletion—a model that recapitulates a primary mitochondrial respiratory chain disease^38^. The deletion of *Cox10* in PT cells triggered mitochondrial alterations that are not linked to anomalies in mitophagy-mediated clearance and quality control systems, in contrast with the dysregulation induced by *Mmut* deletion. The possibility of a cumulative effect of the metabolic and/or mitochondrial perturbations resulting from the *MMUT* deficiency and the loss of PINK1/Parkin-mediated quality control systems is also supported by the observation that patients harbouring loss-of-function mutations of *PINK1* causing Parkinson’s disease survive in the absence of functional PINK1-dependent mitophagy pathway^3^. In these patients, locomotor symptoms do not usually manifest until the second and third decades of life, when a sufficient level of mitochondrial dysfunction is coupled to the loss of PINK1 to have a detrimental effect on neuron integrity^61^.

The mechanisms by which *MMUT* deficiency suppresses PINK1 signalling and mitophagy remain elusive. We speculate that *MMUT* deficiency might alter the stability of PINK1 by disabling the interaction with yet-to-be defined factors that protect PINK1 from processing and degradation^62^. Alternatively, *MMUT* deficiency might trigger stress-related post-translational modification such as *S*-nitrosylation that inhibits PINK1 kinase activity, hence mitophagy^63^. Regardless of the mechanism(s) involved, these findings support a role of the enzyme MMUT—beyond its function in metabolism—in maintaining the mitochondrial quality control system, hence epithelial integrity and homeostasis.

There is an urgent need to identify targetable interventions in the early course of MMA. Previous studies showed that kidney dysfunction and levels of circulating Lcn2 could be abrogated in a transgenic mouse model of MMA by administrating ubiquinone, a bioavailable form of CoQ10 that acts on mitochondria, and Vitamin E^10^. Using an in silico approach based on matching gene expression profile from patient-derived kidney cells against a large compendium of small bioactive drug compounds, we inferred that targeting mitochondrial oxidative stress might reverse disease phenotypes associated with *MMUT* deficiency. In particular, we explored the potential benefit of mitochondria-targeted antioxidants, which are clinically tested in a variety of diseases^64^. Treatment of MMA kidney cells with mito-TEMPO restored partially mitochondrial homeostasis, improved mitochondrial function, normalized mitochondrial ROS and autophagy markers, and prevented the overproduction of LCN2. Furthermore, treatment with low, non-toxic doses of MitoQ alleviated the mitochondrial oxidative stress and ameliorated behavioural phenotypes and blunted the excessive mortality in the *mmut*-deficient zebrafish model of MMA. Importantly, both pharmacological interventions did not modify the levels of MMA metabolite in either MMA cells or *mmut*-deficient zebrafish, supporting the concept that mitochondrial targeting acts independently of the elevation of toxic MMA metabolites.

In conclusion, we identify a pathway that links a genetic deficiency of a mitochondrial enzyme with mitophagy dysfunction and accumulation of damaged mitochondria that generate epithelial stress and tissue damage. These findings substantiate the role of PINK1/Parkin-directed mitophagy in safeguarding mitochondrial network homeostasis, which is crucial for the properly functioning of specialized epithelial cells. Antioxidant compounds specifically targeting mitochondria offer a promising therapeutic strategy for repairing mitochondria in MMA and other mitochondrial disorders.

## Methods

### Antibodies, reagents and cell lines

Anti-MMUT (Abcam, ab67869, 1:200), anti-SDH (Abcam, ab14715, 1:500), anti-PDHA1 (Abcam, ab110334, 1:500), anti-MT-CO2 (Abcam, ab110258, 1:500), anti-VDAC1 (Cell Signaling Technology, 4866, 1:500), anti-COX IV (Abcam, ab14744, 1:500), anti-cytochrome *C* (Abcam, ab110325, 1:500), anti-SOD1 (Santa Cruz Biotechnology, SC-11407, 1:500), anti-LCN2 (Abcam, ab63929, 1:500), anti-ATP5B (Abcam, ab14730, 1:500), anti-LC3 (MBL, PM036, 1:200), anti-phospho ULK1 (Ser757; Cell Signaling Technology, 6888, 1:200), anti-phospho-S6 Ribosomal Protein (Ser235/236) (Cell Signaling Technology, 4858, 1:500), anti-S6 Ribosomal Protein (Cell Signaling Technology, 2217, 1:500), anti-phospho-4E-BP1 (Ser65; Cell Signaling Technology, 9451, 1:500), anti-4E-BP1 (Cell Signaling Technology, 9644, 1:500), anti-ULK1 (Cell Signaling Technology, 8054, 1:200), anti-FIP200 (Cell Signaling Technology, 12436, 1:500), anti-ATG13 (Cell Signaling Technology, 13468, 1:200), anti-WIPI2 (Abcam, ab105459, 1:200), mCherry-2XFYVE Ptdins-3P-binding domain (1:100) were kindly provided by Dr. J. Gallop (University of Cambridge), anti-Parkin (Abcam, ab77924, 1:500 for IF and immunoblotting analyses), anti-Parkin (Santa Cruz Biotechnology, SC-32282, 1:500; for immunoblotting analyses), anti-HA (Roche, 11867423001, 1:500), anti-α-tubulin (Sigma Aldrich, T5168, 1:10,000), anti-RFP (600-401-379, ROCKLAND), anti-UMOD (Meridian, K90071C, 1:500), anti-AQP2 (Santa Cruz Biotechnology, sc-9882, 1:500), anti-CD3 (Abcam, ab16669, 1:200), anti-Ly6G (Biosciences, 551459, 1:100), anti-LAMP1 (Santa Cruz Biotechnology, sc-19992, 1/1000), anti-PMP70 (Sigma, SAB4200181, 1:500), anti-ubiquitin (Santa Cruz Biotechnology, sc-8017, 1:1000), anti-SQSTM1/p62 (MBL, PM045, 1:200), anti-cathepsin-D (Santa Cruz Biotechnology, sc-6486, 1:500), anti-Rab7 (Abcam, 126712, 1/400), anti-β-actin (Sigma, A5441, 1/10,000), anti-ATG7 (Sigma, A2856,1/500), anti-GAPDH (Cell Signaling Technology, 2118, 1:1000), Picro Sirius Red staining kit (Abcam, ab150681) were used. Compounds included BfnA1 (Enzo Life Sciences, ALX-380-030, 250 μM), Rotenone (Sigma, R8875, 5 μM), mito-TEMPO (Enzo Life Sciences, ALX-430-150-M005, 10  μM), MG132 (Abcam, ab141003, 50 μM), MitoQ (Focus Biomolecules, 10-1363, 200 nM), SAR405 (APExBIO, A8883; 5 μM), Oligomycin A (Sigma Aldrich, 495455, 4 μM), and Antimycin A (Sigma Aldrich, A8674, 0.8 μM). The HeLa cell line was kindly provided by Dr. L. Borsig (University of Zurich) and the Hap-1 cell line was purchased from Horizon Discovery (www.horizondiscovery.com). The cells lines used here do not appear in the database of commonly misidentified cell lines (International Cell Line Authentication Committee), except the HeLa cell line that was analysed for the detection of Parkin expression in Supplementary Fig. 7f. All of the cells used in this study were negatively tested for mycoplasma contamination using MycoAlert™ Mycoplasma Detection Kit (LT07-118, Lonza, Switzerland).

### Human kidney biopsies

Human kidney biopsies were obtained from an individual patient with clinical diagnosis of MMA and from a healthy control (non-transplanted, normal human kidney). Informed consent was obtained, and the use of the human biopsy samples was in accordance with the ethical regulations at Bambino Gesù Children’s Hospital and approved by the EURenOmics consortium (FP7, 2007–2013, grant agreement no. 305608) and by the institutional review board at Bambino Gesù Children’s Hospital.

### Immunofluorescence on human kidney samples

Paraffin blocks of human kidney samples were sectioned into consecutive slices with a thickness of 6 μm using a Leica RM2255 rotary microtome (Thermo-Fisher Scientific) on Superfrost Plus glass slides (12-550-15, Thermo-Fisher Scientific). Before staining, slides were deparaffinized in changes of CitriSolv (22-143-975, Thermo-Fisher Scientific) and 70% isopropanol. Antigen retrieval was accomplished by incubating in sodium citrate buffer (1.8% 0.1 M citric acid, 8.2% 0.1 M sodium citrate, in distilled water, pH 6.0) in a rice cooker for 30 min. The slides were blocked with phosphate-buffered saline (PBS) blocking buffer (1% BSA, 0.2% non-fat dry milk in PBS) for 30 min and stained with primary antibody specific for LC3 and SQSTM1/p62 in blocking buffer overnight at 4 °C. After two washes in 0.1% Tween 20 (v/v in PBS), the slides were incubated with the corresponding fluorophore-conjugated secondary antibodies (Life Technologies) diluted in blocking buffer at room temperature for 1 h and counterstained with 1 μg Biotinylated Lotus Tetragonolobus Lectin (LTL; B-1325 Vector Laboratories) and 1 µM 4′,6-diamino-2-phenylindole dihydrochloride (DAPI; #D1306, Thermo Fischer Scientific). The slides were subsequently mounted in Prolong Gold Anti-fade reagent (Life Technologies) and images were acquired using the Leica SP8 confocal laser scanning microscope (Center for Microscopy and Image Analysis, University of Zurich) as described below.

### Electron microscopy on human kidney samples

Biopsy specimens were immersed in ice-cold 2.5% glutaraldehyde in 0.1 M cacodylate buffer (pH 7.4) immediately after their extraction and sectioning, and fixed for 4 h at 4 °C, carefully handling samples to avoid ex vivo artefacts. After washing in cacodylate buffer, kidney fragments were then post-fixed in 1% osmium tetroxide for 1 h, dehydrated through ascending grades of alcohol, and embedded in Epon resin (Electron Microscopy Science, Hatfield, PA). Ultrathin sections (70–75 nm) were cut on an ultramicrotome (Leica reichert ultracut S), stained with uranyl acetate and lead citrate, and examined with TEM (Jeol 1400 PLUS).

### Measurement of the MMUT enzyme activity

Crude cell homogenates were sonicated and 1 mM dl-2-[methyl-^14^C] methylmalonyl-CoA (ARC; specific activity 7.03 MBq/mmol in assay) was added in the presence (total MMUT activity) and in the absence (holo-MMUT activity) of the cofactor Adenosylcobalamin (AdoCbl; 50 µM) in darkroom safelight red conditions. The reaction was terminated by the addition of 5 N KOH (Merck, Darmstadt, Germany). The samples were enriched with succinic acid (Merck, Darmstadt, Germany) to visualize the succinate peak during HPLC separation. Succinate and methyl malonate peaks were detected at 210 nm by an UV detector. Quantification of the [^14^C] succinate fraction was performed with Optiphase HiSafe2 counting cocktail (PerkinElmer) in a Tri-Carc C1 900TR scintillator spectrometer (Packard). The protein concentration of the cell lysates was determined using the Lowry method. The activity of the enzyme MMUT is expressed as pmol succinate which is formed per minute per mg protein [pmol/min/mg].

### Measurement of the methylmalonic acid

Mouse tissues or cells were sonicated and lysed in 100 µL of acetonitrile/10 mM ammonium formate 1:1 (v/v). The concentration of methylmalonic acid was measured in an accredited laboratory using the standard protocol from Recipe (Recipe, Munich, Germany) with some minor adaptations. Briefly, 50 µL of lysate or urine were mixed with 200 µL precipitation reagent containing the internal standard d_3_-methylmalonic acid (Recipe, Munich, Germany). After vortexing the sample for 30 s, the protein precipitate was removed by centrifugation for 5 min at 16,000 *g* and the clean supernatant is transferred to a LC-MS vial, which is kept at 10 °C in a thermostatic autosampler until LC-MS analysis. From each vial, 2 µL of sample were loaded and separated on an ClinMass column (2.1 × 100 mm, Recipe, Munich, Germany), kept at 25 °C using an Ultimate 3000XRS (Thermo Scientific, Olten, Switzerland) instrument interfaced to a SCIEX TripleQuad 5500 (AB Sciex, Zug, Switzerland) mass spectrometer. The chromatography was performed at a flow rate ~700 µL/min by using mobile phase A (MPA) and B (MPB) from Recipe (Recipe, Munich, Germany). The elution was achieved using a step gradient (100% MPA for 0.3 min, 70% MPA for 0.3 min, 40% MPA for 0.7 min and 0% MPA for 0.1 min followed by 1.2 min at 100% MPA). The source parameters were the following: CAD = 7 psi, CUR = 30 psi, GS1 = 60 psi, GS2 = 70 psi, IS = 4.0 kV, TEM = 550 °C. Multiple reaction monitoring (MRM) in negative ion mode is used to identify and quantify methylmalonic acid (quan: 117/73; qual: 117/55) and d_3_-methylmalonic acid (quan: 120/76; qual: 120/58). A collision energy of −12V is used for the 117/73 and 120/76 transitions, while a collision energy of −31V is used for the 117/55 and 120/58 transitions. The declustering potential was −40 V for all four transitions.

### Kidney tubular cells from urines of patients with MMA

Tubular epithelial cells were derived from urines of either three healthy donors or three *mut*^*o*^ MMA patients (Supplementary Table 1, ref. ^25^ and cultured in a selective medium containing Dulbecco’s modified Eagle's medium and Ham’s F12 medium, supplemented with dialyzed fetal calf serum (FCS), insulin, hydrocortisone, selenite, transferrin, hEGF, NAD, and 3,3,5 triiodo-l-thyronine. The identity of *MMUT* mutations were confirmed by Sanger sequencing. Confluent cells were sub-cultivated until third passage and, subsequently, immortalized using pRSVneo vector containing SV40 DNA (pRNS1). Afterwards, immortalized kidney tubule epithelial cells were characterized for morphology and expression of kidney markers, and MMUT protein and its enzymatic activity as described previously^25^. Where indicated, lysosomal proteolysis was inhibited by adding BfnA1 (250 nM for 2 h and 4 h, unless otherwise stated). Where indicated, the mitochondrial damage and the activation of mitophagy were triggered by treating the cells with Rotenone (5 μM for 24 h) or with Oligomycin A and Antimycin (O/A; 4 and 0.8 μM, respectively), in fresh culture medium for the indicated times. Where indicated, the cells were starved by washing them with Hank’s balanced salt solution (55021C, Sigma Aldrich) and placing them in nutrient-deprived medium. Where indicated, the autophagy was inhibited by culturing the cells in presence or in absence of a highly selective PIK3C3/Vps34 inhibitor SAR405 (5 μM in fresh culture medium for 4 h). Where indicated, the cells were treated with the proteasome inhibitor MG132 (5 μM in fresh culture medium). Where indicated, the cells were treated with the mitochondrially targeted ROS scavenger Mito-TEMPO (MT; 10 μM in fresh culture medium for 24 h; Enzo Life Sciences). The cells were processed and analysed as described below.

### Generation of *PINK1* and *PRKN2* knockout cell lines

*PRKN2* (HZGHC003208c002) and *PINK1* (HZGHC000798c008) KO cells were created through CRISPR-Cas9 gene editing technology and purchased from Horizon Discovery (www.horizondiscovery.com). The cells were tested negative for mycoplasma contamination and subjected to PCR analysis, followed by Sanger sequencing to identify CRISPR-Cas9-induced deletion. The cells were maintained in Iscove’s modified Dulbecco’s medium (IMDM) supplemented with 10% dialyzed FCS, 100 U/mL Penicillin, and 100 μg/mL Streptomycin. The cells were afterwards processed and analysed as described below.

### Generation and maintenance of *mmut* zebrafish

pT7-gRNA and pT3TS-nCas9n plasmids were obtained from Addgene (# 46759 and # 46757, respectively). CRISPR-Cas9-targeted site (5′-GGGCCAGCAGGGTCTGTCTGTGG-3′) contains a restriction site for AlwNI (CAGNNNCTG), which has been used for monitoring CRISPR-Cas9-mediated mutation and genotyping. Oligonucleotides containing sgRNA-targeting sequence were annealed and cloned into the Esp3I (*Bsm*BI)-digested pT7-gRNA vector (pT7-gRNA-*mmut*). The sequence of oligonucleotides is CRISPR-*mmut-*S: 5′-TAGGGCCAGCAGGGTCTGTCTG-3′ and CRISPR-*mmut*-AS: 5′-AAACCAG ACAGACCCTGCTGGC-3′. XbaI-linearized pT3TS-nCas9n vector was used to produce Cas9 mRNA using mMESSAGE mMACHINE T3 kit (Invitrogen). Both sgRNA-*mmut* and Cas9 messenger RNAs were co-injected into two or four-cell stage TU zebrafish (*Danio rerio*) embryos. For detection of CRISPR-Cas9-induced deletion, genomic DNA was extracted from 2dpf embryos which developed normally. The CRISPR-Cas9-injected mosaic TU embryos were raised to adulthood (F0) and outcrossed with wild-type TL zebrafish. The embryos were then raised to adulthood (F1) for screening of heterozygous carriers. We identified a heterozygous carrier harbouring *mmut*^+/del11^ mutation and (F1) generations were crossed again with wild-type TL zebrafish to generate (F2) heterozygous zebrafish. Homozygous zebrafish larvae carrying *mmut*^del11/del11^ mutation were obtained from in-cross of (F2) heterozygous zebrafish. Zebrafish were kept at day/night cycle of 14/10 h at 28 °C. Zebrafish larvae were anaesthetised by immersion in E3 medium (for 5 dpf) or system water (for 10 dpf) containing 168 µg mL^−1^ tricaine methane sulfonate (MS222, Sigma Aldrich) and analysed as described below. Animal care and experimental procedures were approved by the institutional animal care and use committee at Canton Zurich in accordance with the ethical guidelines at University of Zurich, Switzerland.

### Rescue experiments in *mmut* zebrafish

A transgenic line expressing *mmut*-mCherry was generated under the control of the liver fatty acid-binding protein (*lfabp or fabp10a*) promoter. Primers used to clone *lfabp* promoter are *lfabp*-Fwd: 5′-AAATGCAAATTCTGAGCAAATGAC-3′; and *lfabp*-Rev: 5′-GCTTTCTGGAGAAGCTCAACA-3′. Primers used for amplification of mut cDNA are *mut*-Fwd: 5′-CCCGATGCCTACATAACAACA-3′; and *mut*-Rev: 5′-GAACCACCTGATGGTGAGTGA-3′. Stable zebrafish line expressing *mmut*-mCherry in the liver was established and outcrossed with *mmut*^*+/del11*^zebrafish to generate transgenic mutant line, which was crossed with *mmut*^*+/del11*^zebrafish to produce homozygous larvae. Ten zebrafish embryos at 5 or 10 dpf were pooled and homogenized by sonication in solution containing 50% acetonitrile and 10 mM ammoniaformate, and prepared for methylmalonic acid measurements. Where indicated, zebrafish larvae were treated at 9 dpf with system water containing DMSO or MitoQ (200 nM; Focus Biomolecules) for 24 h. The fluorescent signal of reporter protein mito-Grx1-roGFP2 (as described below) and the larval swimming were analysed at 10 dpf. For larval movement tracking, 10 dpf zebrafish larvae were kept individually in fish system water in a 24-well plate. After 10 min of adaptation, the larvae swimming tracking is recorded and analysed in live with zebrabox (ViewPoint) with light stimulation during 5 min. Where indicated, 5-dpf zebrafish were fed with a low-protein diet (kindly provided by Dr. Carvalho, University of Porto) until to the sampled day (14 dpf), and the distribution of *mmut* zebrafish larvae was assessed. A range of 100‒120 embryos obtained from in-cross strategies between heterozygous zebrafish were raised to the sampled day, and the percentage of wild type, heterozygous, and homozygous were determined after genotyping procedure.

### Detection of mitochondrial ROS in *mmut* zebrafish

To measure mitochondrial ROS in zebrafish larvae, we generated a transgenic line expressing the reporter protein mito-Grx1-roGFP2 under the control of the liver fatty acid-binding protein (*lfabp*) promoter. The plasmid pLPCX-mito-Grx1-roGFP2 was kindly provided from Dr. Dick (Addgene plasmid # 64977). Plasmid DNA *pDestTol2CG2-lfabp::mito-Grx1-roGFP2* was co-injected with Tol2 transposase mRNA into zebrafish embryo at one-cell stage. Mosaic larvae expressing *lfabp::mito‒Grx1‒roGFP2* were raised to adulthood and then outcrossed with *mmut*^+/del11^ zebrafish to produce *mmut*^+/del11^ zebrafish expressing mito-Grx1-roGFP2 in the liver. The fluorescent signal of Grx1-roGFP2 is analysed using  light-sheet microscope (Zeiss, Z.1) after excitation at 405 and 488 nm and detected through the same emission filter (505‒545 nm). Imaging settings were maintained with the same parameters for comparison between different experimental conditions. The acquired data were processed by Huygens software for deconvolution, and the mean fluorescence intensities of randomly selected ROIs were measured and expressed as the blue/green fluorescence intensity ratio.

### Mouse models

The mice were maintained under temperature- and humidity-controlled conditions with 12 h light/12 h dark cycles with free access to appropriate standard diet in accordance with the institutional guidelines of National Institutes of Health Guide for the Care and Use of Laboratory Animals. The mice bearing floxed *Cox10* (*Cox10* ^fl/fl^) alleles, in which the exon 6 is flanked by two *loxP* sequences, were kindly provided by Dr. Moraes (Department of Neurology, University of Miami). The mice bearing floxed *Atg7* (*Atg7* ^fl/fl^) alleles were kindly provided by the RIKEN BRC through the National-Bio-Resource Project of the MEXT, Japan. The mice bearing germline *Prkn2* and *Pink1* knockout were purchased from Jackson Laboratory. The mice bearing floxed *Mmut* (*Mmut* ^fl/fl^) alleles, in which the exon 3 is flanked by two *loxP* sequences, and the mice carrying M698K point mutation in the *Mmut* gene was performed by Polygene (Rümlang, Switzerland) using the C57Bl/6-derived embryonic stem cell targeting. To obtain *Mmut* ^KO/KI^ mice, females *Mmut* ^WT/KO^ were crossed to *Mmut* ^KI/KI^ males as previously described^29^. Mouse genotyping was performed on genomic DNA extracted from ear punch biopsies using the primers 5′-GTGGGTGTCAGCACACTTG-3′ (forward) and 5′-CGTATGACTGGGATGCCT-3′ (reverse) for the KI allele and 5′-ACAACTCCTTGTGTAGGTC-3′ (forward) and 5′-CCTTTAGGATGTCATTCTG-3′ (reverse) for the KO allele. All the mice were maintained on a C57BL/6 background. Animal care and experimental procedures were approved by the institutional animal care and use committee at Canton Zurich in accordance with the ethical guidelines at University of Zurich, Switzerland.

### Kidney function

The mice were placed overnight in metabolic cages with ad libitum access to food and drinking water; urine was collected on ice, body weight, water intake, and diuresis were measured^46^. Blood (from sublingual vein) was obtained after anaesthesia with ketamine/xylazine or isoflurane. Urea and creatinine were measured using UniCel DxC 800 pro Synchron (Beckman Coulter, Fullerton, CA, USA). The creatinine clearance was calculated using the equation Urine_(creatinine)_ × Diuresis/Plasma_(creatinine)_. Plasma and urinary levels of Lcn2 were measured by using an enzyme-linked immunosorbent assay in according to the manufacturer’s instructions (EMLCN2, Thermo Fischer Scientific, Waltham, MA).

### Picro Sirius Red staining

Picro Sirius Red (ab150681, Abcam) staining was performed on 5-μm-thick paraffin sections from kidneys of *Mmut*^*WT/KI*^ and *Mmut*^*KO/KI*^. Briefly, the sections were deparaffinized, rehydrated and subsequently incubated with Picro Sirius Red for 1 h at room temperature. Sections were then washed twice in acidified water (0.05% acetic acid in distilled water), dehydrated and mounted. Images were acquired with a ZEISS AxioScan.Z1 slide scanner (ZEISS).

### Primary cultures of mouse proximal tubular cells

The kidneys were harvested from *Mmut* ^WT/KI^ and *Mmut* ^KI/KO^ or from *Mmut* ^fl/fl^ or *Atg7* ^fl/fl^ or *Cox10* ^fl/fl^, and from *Pink1* or *Prkn2* KO and from their corresponding control littermates: one kidney was split transversally, and one half was fixed and processed for immunostaining while the other half was flash-frozen, homogenized by Dounce homogenizer in 1 mL of RIPA buffer that contains protease and phosphatase inhibitors and processed for western blot analysis^46^. The contralateral kidney was taken to generate primary cultures of mPTCs^46^. Freshly micro-dissected PT segments were seeded onto collagen-coated chamber slides (81156, ibidi) and/or collagen-coated 6- or 24-well plates (145380 or 142475, Thermo-Fisher Scientific), and cultured at 37 °C and 5% CO_2_ in DMEM/F12 (21041-025, Thermo-Fisher Scientific) with 0.5% dialyzed fetal bovine serum (FBS), 15 mM HEPES (H0887, Sigma Aldrich), 0.55 mM sodium pyruvate (P2256, Sigma Aldrich), 0.1 mL L^−^^1^ non-essential amino acids (M7145, Sigma Aldrich), hydrocortisone, human EGF, epinephrine, insulin, triiodothyronine, TF, and gentamicin/amphotericin (Single Quots® kit, CC-4127, Lonza), pH 7.40, 325 mOsm kg^-1^. The medium was replaced every 48 h. Confluent monolayers of mPTCs were expanded from the tubular fragments after 6–7 days, characterized by a high endocytic uptake capacity. These cells were negative tested for mycoplasma contamination. All experiments were performed on confluent monolayers grown on chamber slides or 24-well or 6-well tissue culture plates. Where indicated, the mitochondrial damage and mitophagy were triggered by treating primary cultures of PT cells with Rotenone (5 μM in the fresh culture medium for the indicated time) Oligomycin A and Antimycin (4 and 0.8 μM, respectively, in the fresh culture medium for the indicated time). Afterwards, the cells were processed and analysed as described below.

### Adenovirus transduction

For RNA interference studies, the adenovirus constructs include scrambled short hairpin (Scmb-shRNA) or shRNAs encoding individually mouse *Atg7*. For expression studies, adenovirus constructs used include CMV (control vector, Ad-CMV-GFP, Vector Biolabs) or an individually carrying Cre-recombinase (Ad-Cre-GFP, Vector Biolabs) or carrying human hemagglutinin (HA)-tagged *PINK1*, or carrying mouse green fluorescence protein (GFP)-tagged *Map1lc3b* or expressing green fluorescent protein (GFP) or the coral-derived protein Keima with the mitochondrially targeting sequence of the human cytochrome *C* oxidase subunit VIII (COXVIII). All adenovirus constructs were purchased from Vector Biolabs (University City Science Center, Philadelphia, USA). The cells were plated onto collagen-coated chamber slides or 24- or 6-well tissue culture plates. Adenovirus transduction was performed 24 h after plating when the cells reached approximately 70–80% confluence. The cells were subsequently incubated for 16 h at 37 °C with culture containing the virus at the concentration (0.2125 × 10^9^ PFU mL^−^^1^). The cells were afterwards challenged with fresh culture medium every 2 days, cultured for 5 days (unless otherwise specified) and collected for analyses.

### Microarray and drug–disease network modelling

The Affymetrix Gene-Chip (HG-U113A) hybridization experiments were performed in triplicate at the Coriell Genotyping and Microarray Center, Coriell Institute for Medical Research, Camden, New Jersey, USA, on total RNA extracted from tubular cells derived from three healthy donors and three *mut*^*o*^ MMA patients (Supplementary Table 1; ref. ^25^). To identify downstream transcriptional effects of loss-of-MMUT function, microarray data were pre–processed using the Bioconductor package Affy52 and normalized with the RMA method^65^. Differentially expressed (DE) genes between conditions (MMA patient-derived versus their control cells) were identified using a Bayesian *t*-test^66^. For each *P* value, the Benjamin–Hochberg procedure was used to calculate the false discovery rate (FDR) to avoid the problem of multiple testing. Mode of Action by Network Analysis (Mantra 2.0, http://mantra.tigem.it) and Drug Set Enrichment Analysis (DSEA, http://dsea.tigem.it) were employed to predict either effective therapeutics or druggable biological processes in MMA patient-derived kidney cells. Taking advantage of the connectivity map data set —which includes transcriptional profiles following treatment of 1309 small molecules across five different cell lines—Mantra aims to identify compounds transcriptionally similar or different to a disease profile just providing as input the ranked list of genes sorted according to their differential expression. This analysis enabled us to capture the top-ranked small bioactive molecules that transcriptionally reverse the MMA gene signature molecular pathways consistently up- or downregulated by these set of drugs were then detected using DSEA.

### Reverse transcription-quantitative PCR

Total RNA was extracted from mouse tissues using Aurum ^**TM**^ Total RNA Fatty and Fibrous Tissue Kit (Bio-Rad, Hercules, CA). DNAse I treatment was performed to eliminate genomic DNA contamination. Total RNA was extracted from cell cultures with RNAqueous^R^ kit (Applied Biosystems, Life Technologies). One microgram of RNA was used to perform the reverse transcriptase reaction with iScript ^**TM**^ cDNA Synthesis Kit (Bio-Rad). Changes in mRNA levels of the target genes were determined by relative RT–qPCR with a CFX96^TM^ Real-Time PCR Detection System (Bio-Rad) using iQ ^**TM**^ SYBR Green Supermix (Bio-Rad). The analyses were performed in duplicate with 100 nM of both sense and anti-sense primers in a final volume of 20 µL using iQ^TM^ SYBR Green Supermix (Bio-Rad). Specific primers were designed using Primer3 (Supplementary Tables 2–4). PCR conditions were 95 °C for 3 min followed by 40 cycles of 15 sec at 95 °C, 30 s at 60 °C. The PCR products were sequenced with the BigDye terminator kit (PerkinElmer Applied Biosystems) using ABI3100 capillary sequencer (PerkinElmer Applied Biosystems). The efficiency of each set of primers was determined by dilution curves (Supplementary Tables 2–4). The program geNorm version 3.4 was applied to characterize the expression stability of the candidate reference genes in kidneys and six reference genes were selected to calculate the normalization factor. The relative changes in targeted genes over *Gapdh* mRNAs were calculated using the 2^−ΔΔCt^ formula. For the absolute quantification of mitochondrial:nuclear DNA ratio, relative values for *ND1* and *ACTB* (in human cells) or for *Nd1* and *Hbb* (in murine cells) were compared within each sample to generate a ratio representing the relative level of mitochondrial DNA per nuclear genome. Primers used for mitochondrial DNA: nuclear DNA ratio in human cells are *ND1*-Fwd: 5′-ACACTAGCAGAGACCAACCG-3′ and *ND1*-Rev: 5′-GAAGAATAGGGCGAAGGGGC-3′; *ACTB*-Fwd: 5′-TCACCCACACTGTGCCCATCTACGA-3′ and *ACTB*-Rev: 5′-CAGCGGAACCGCTCATTGCCAATGG-3′. Primers used for mitochondrial DNA:nuclear DNA ratio in murine cells are *Nd1*-Fwd: 5′-TAGAACGCAAAATCTTAGGG-3′ and *Nd1*-Rev: 5′-TGCTAGTGTGAGTGATAGGG-3′; *Hbb*-Fwd: 5′-AGGCAGAGGCAGGCAGAT-3′ and *Hbb-*Rev: 5′-GGCGGGAGGTTTGAGACA-3′.

### Lysosome-based cellular degradation

The detection of lysosomal activity was performed in live in kidney cells by using Bodipy-FL-Pepstatin A (P12271, Thermo Fischer Scientific) according to the manufacturer’s specifications. The cells were pulsed with 1 μM Bodipy-FL-Pepstatin A in Live Cell Imaging medium (A14291DJ, Thermo Fischer Scientific) for 1 h at 37 °C, fixed, and subsequently analysed by confocal microscopy^46^. The numbers of PepA-positive structures per cell were quantified by using the open-source cell image analysis software CellProfiler^TM^ as described below.

### Mitochondrial membrane potential

The mitochondrial membrane potential (Δ*ψ*) was measured in accordance with the manufacturer’s specifications. The cells were pulsed with 50 nM tetramethylrhodamine methyl ester perchlorate (TMRM, T668 Thermo-Fisher Scientific) and 1 μM MitoTracker Red FM (Invitrogen, M22426) for 30 min in live cell imaging at 37 °C. After washing, the cells were subsequently analysed by confocal microscopy^46^ in a chamber heated to 37 °C at 5% CO_2_. Images were acquired using Leica SP8 confocal laser scanning microscope (Center for Microscopy and Image Analysis, University of Zurich) and the fluorescence intensity was quantified by the open source image processing Fiji (which is just ImageJ, NIH) as described below.

### Detection of mitochondrial ROS

The cells were pulsed with 2.5 μM MitoSOX Red Mitochondrial Superoxide Indicator (M36008, Thermo-Fisher Scientific) and 1 μM MitoTracker Green FM (Invitrogen, M7514) for 30 min in live cell imaging at 37 °C. After washing, the cells were subsequently analysed by confocal microscopy in a chamber heated to 37 °C at 5% CO_2_. Images were acquired using a Leica SP8 confocal laser scanning microscope (Center for Microscopy and Image Analysis, University of Zurich) and the fluorescence intensity was quantified by the open source image processing Fiji (which is just ImageJ, NIH) as described below.

### mt-Keima-based mitophagy assay

Cells expressing mt-Keima were treated in the presence and in the absence of mitochondrial complex I inhibitor Rotenone or electron transport chain inhibitors Oligomycin A and Antimycin as previously described and analysed by confocal microscopy in a chamber heated to 37 °C at 5% CO_2_. mt-Keima protein was excited both at 458 nm (neutral, pseudo-coloured in green) and 561 nm (acidic, pseudo-coloured in red) and detected through the same emission filter (570‒695 nm). Laser power was set up at the lowest output which would enable the clear visualization of the mt-Keima signal, and were individualized for each experimental condition. Imaging settings were maintained with the same parameters for comparison between different experimental conditions. Images were acquired using a Leica SP8 confocal laser scanning microscope (Center for Microscopy and Image Analysis, University of Zurich) and the fluorescence intensity was quantified by the open source image processing software Fiji (ImageJ, NIH) as described below.

### Measurement of oxygen consumption rate and metabolic profiling

OCR in mouse or human kidney tubule cells and in zebrafish larvae was measured with XFp Extracellular Flux Analyzers (Agilent Seahorse Biosciences). The cells were incubated with XF-Base Medium (non-buffered RPMI 1640 containing either 2 mM l-glutamine, 1 mM sodium pyruvate, and 10 mM glucose, pH 7.4). Three measurements were assessed under basal conditions and upon addition of 2 μM Oligomycin (Oligo), 0.5 μM FCCP, and 1 μM Rotenone (ROT)/Antimycin A (ANT). All the reagents were provided by XFp Cell Mito Stress Test Kit (Agilent Seahorse Biosciences). OCR measurements were normalized to the numbers of cells (TC10^TM^ automated cell counter, Bio-Rad). The zebrafish larvae were anaesthetised with 125 mg/L of MS222 and placed individually into the wells of a 24-wells microplate, filled with 500 mL of E3 media (pH 7.4) containing anaesthesia solution MS222 and covered by capture screens. After incubation in a non-CO_2_ incubator at 28.5 °C for 20 min, the metabolic measurements were analysed in live. One measurement cycle consisted of a brief wait period to acclimate the plate, 2 min mix, 1 min wait, and 2 min data acquisition. Eight measurement cycles were performed to establish the average value.

### Detection of Ptd-Ins3P

Ptd-Ins3P staining was performed according to previously established protocols^67^. Briefly, the cells were fixed for 15 min in 2% PFA and permeabilized for 5 min with 20 μM digitonin in buffer A (150 mM NaCl, 20 mM HEPES, pH 7.4, and 2 mM EDTA). Cells were subsequently incubated for 45 min with buffer A supplemented with 5% goat serum and mCherry-2× FYVE Ptd-Ins3P-binding domain and immunostained with the anti-RFP antibody to amplify the detection of mCherry probe. The cells were washed and post-fixed with 2% PFA for 5 min, and analysed by confocal microscopy and quantified by using the open-source cell image analysis software CellProfiler^TM^ as described below.

### Immunofluorescence and confocal microscopy

Fresh mouse kidneys were fixed by perfusion with 50–60 mL of 4% paraformaldehyde in PBS (158127, Sigma Aldrich), dehydrated and embedded in paraffin at 58 C. Paraffin blocks were sectioned into consecutive 5-μm-thick slices with a Leica RM2255 rotary microtome (Thermo-Fisher Scientific) on Superfrost Plus glass slides (Thermo-Fisher Scientific). Before staining, slides were deparaffinized in changes of CitriSolv (22-143-975, Thermo-Fisher Scientific) and 70% isopropanol. Antigen retrieval was accomplished by heating the slides at 95 °C for 10 min in 10 mM sodium citrate buffer (pH 6.0). The slides were quenched with 50 mM NH_4_Cl, blocked with 3% BSA in PBS Ca/Mg (D1283, Sigma Aldrich) for 30 min and stained with primary antibodies specific for Mutase, UMOD, AQP2, CD3, Ly6G diluted in blocking buffer overnight at 4 °C. After two washes in 0.1% Tween 20 (v/v in PBS), the slides were incubated with the corresponding fluorophore-conjugated Alexa secondary antibodies (Invitrogen) diluted in blocking buffer at room temperature for 1 h and counterstained with 1 μg Biotinylated Lotus Tetragonolobus Lectin (LTL; B-1325 Vector Laboratories) and 1 µM DAPI (D1306, Thermo Fischer Scientific). The slides were mounted in Prolong Gold Anti-fade reagent (P36930, Thermo Fisher Scientific) and analysed by confocal microscopy. The images were acquired using a Leica SP8 confocal laser scanning microscope (Center for Microscopy and Image Analysis, University of Zurich) equipped with a Leica APO ×63 NA 1.4 oil immersion objective at a definition of 1024 × 1024 pixels (average of 8 or 16 scans), adjusting the pinhole diameter to 1 Airy unit for each emission channel to have all of the intensity values between 1 and 254 (linear range). The micrographs were processed with Adobe Photoshop (version CS5, Adobe System Inc., San Jose, USA) software. Quantitative image analysis was performed by selecting randomly ∼5‒10 visual fields per each slide that included at least 3‒5 PTs (LTL positive), using the same setting parameters (i.e., pinhole, laser power, and offset gain and detector amplification below pixel saturation). The numbers of CD3 or Ly6G-positive structures per field were manually counted.

The cells were fixed for 10 min with 4% PFA in PBS, quenched with 50 mM NH_4_Cl and permeabilized for 20 min in blocking buffer solution containing 0.1% Triton X-100 and 0.5% BSA dissolved in PBS. Subsequently, cells were incubated overnight with the appropriate primary antibodies at 4 °C. After repeated washing with PBS, the slides were incubated for 45 min with the suitable fluorophore-conjugated Alexa secondary antibodies (Invitrogen), counterstained with 1 µM DAPI for 5 min, mounted with the Prolong Gold Anti-fade reagent and analysed by a Leica SP8 confocal laser scanning microscope (Center for Microscopy and Image Analysis, University of Zurich) using the settings described above. Quantitative image analysis was performed by selecting randomly five visual fields pooled from biological triplicates, with each field including at least 10–15 cells, using the same setting parameters (i.e. pinhole, laser power, and offset gain and detector amplification below pixel saturation). The quantitative cell image analyses of mitochondrial morphology^68^ were performed using mitochondrial morphology plug in of the open-source cell image software Fiji. The quantitative cell image of mitochondrial membrane potential, production of mitochondrial ROS and mt-Keima red/green signal, the analyses of fluorescence were performed following background subtraction over cellular regions of interests (ROIs) and quantified using the multi-measure plugin of Fiji. Mean fluorescence intensity ratios of selected ROIs matching cells were determined and expressed as TMRM/MitoTracker, MitoSOX and mt-Keima red/green ratios, respectively. The quantitative cell image analyses of subcellular structures were determined by using the open-source cell image analysis software CellProfiler^TM^^69^. In particular, the specific module “Measure-Object-Intensity-Distribution” was used to score the number of ATP5B, PtdIns-3P, ATG13, WIPI2, Parkin, LC3, and PMP70 or Pep-A^+^ structures. The pipeline “Cell/particle counting and scoring the percentage of stained objects” was used to score either the fractions of Lamp1-positive structures (or mito-GFP positive) that were also positive for LC3 (or Parkin). The number of cells and/or fields of views for analysis for each condition and the number of the independent experiments are indicated in the figure legends accordingly.

### Electron microscopy

Animal tissue samples and cultured cells were fixed in 2.5% glutaraldehyde in 100 mM sodium cacodylate, at pH = 7.43 for 1 h at room temperature, post-fixed in 1% osmium tetroxide, 1.5% potassium ferrocyanide in 0.1 M cacodylate for 1 h on ice. After washing in distilled water, the samples were stained with 0.5% uranyl acetate in water overnight at 4 °C. The samples were finally dehydrated in a graded ethanol series, embedded in Epon 812 and finally cured at 60 °C for 48 h. Ultrathin (70‒90 nm) sections were cut using a Leica Ultracut UCT ultramicrotome and collected on copper grids, stained with uranyl acetate and Sato’s lead citrate before been imaged with a FEI Talos 120 kV transmission electron microscope (FEI Company, Netherlands) and images were acquired by a 4k × 4K Ceta CMOS camera. AVs were identified and categorized as autophagosomes or autolysosomes according to conventional criteria^31^. Autophagosome should have a double membrane (completely or partially visible), absence of ribosomes in the outer membrane, luminal density comparable to the surrounding cytosol and identifiable organelles or regions of organelles in their lumen; autophagolysosomes should have single membrane, luminal density lower than surrounding cytosol with luminal material partially recognizable as specific organelles or heterogeneous amorphous material. Primary and secondary lysosomes were excluded from the quantification. The term autophagic vacuole was used for identifying both autophagosomes and autophagolysosomes structures. At least 20 random selected cellular profiles were acquired and analysed using ImageJ software. For the quantification of content of AVs, the percentage of cytosolic area occupied by AVs was calculated. For the quantification of the morphology of mitochondrial network, the cross-sectional area (*A*) and perimeter (*P*) derived from each mitochondrion was measured and circularity (*c*) was calculated according to the formula circularity = 4*π* (area/perimeter^2^).

### Correlative light electron microscopy

The cells were grown on finder grids and prepared for confocal microscopy analyses. Z-stacks of cells of interest were taken with the PerkinElmer Ultra View ERS confocal microscope. The coordinates of the cells on the finder grid were determined by bright-field microscopy. Cells were fixed in 1% glutaraldehyde in 0.1 M cacodylate buffer (Sigma) and post-fixed with 1.5% potassium ferricyanide, 1% OsO4 in 0.1 M cacodylate buffer. Cells were stained overnight with 0.5% uranyl acetate, dehydrated in ethanol, and embedded in epon. After baking for 48 h at 60 °C, the resin was released from the glass coverslip by temperature shock in liquid nitrogen. Serial sections (70–90 nm) were collected on carbon-coated formvar slot grids and imaged with a FEI Talos 120 kV transmission electron microscope (FEI Company, Netherlands) and images were acquired by a 4k × 4K Ceta CMOS camera. LM and EM images were aligned using Ec-CLEM Icy plugin and overlaid using Photoshop software.

### Soluble and insoluble fractionation

The cells were lysed in buffer containing 50 mM Tris-HCl, pH 7.5, 150 mM NaCl, 0.1% SDS, 1% Triton X-100, 1% sodium deoxycholate supplemented with protease (1836153001, Roche) and phosphatase inhibitors (04906845001, PhosSTOP Sigma), and centrifuged at 16,000*g* at 4 °C for 20 min to collect the soluble fraction (supernatant). The pellet was suspended in a buffer containing 4% SDS and 20 mM HEPES, pH 7.5, protease and phosphatase inhibitors, and further centrifuged at 18,000 *g* at room temperature for 10 min to collect the insoluble fraction (supernatant). The samples were boiled at 95 °C for 5 min and analysed by western blotting.

### Immunoblotting

Proteins were extracted from mouse tissues or cultured cells, lysed using a buffer which contains protease (1836153001, Roche) and phosphatase inhibitors (04906845001, PhosSTOP Roche), followed by sonication and centrifugation at 16,000*g* for 10 min at 4 °C. The samples were thawed on ice, normalized for protein (20 μg per lane), dissolved in Laemmli sample buffer and separated by SDS-PAGE under reducing conditions. After blotting onto PVDF and blocking with 5% non-fat milk (1706404, Bio-Rad Laboratories), the membranes were incubated overnight at 4 °C with primary antibody, washed, incubated with peroxidase-labelled secondary antibody and visualized with enhanced chemiluminescence (WBKLS0050, Millipore, Sigma). Signal intensity was assessed by measuring the relative density of each band normalized to β-actin, GAPDH or α-tubulin with ImageJ software.

### Cell viability assay

Cell viability was determined by the Cell Counting Kit-8 (CCK-8) assay (Dojindo, Rockville, USA). Cells were seeded at a density of 5000 cells/well of 96-well plates and grown until they reached 90% confluence. The cell medium was replaced by water-soluble tetrazolium salt solution and incubated for 30 min at 37 °C according to the manufacturer’s protocol. The amount of formazan dye generated by dehydrogenase activity was measured at 450 nm in a TECAN infinite 200 reader (Männedorf, Switzerland) according to the manufacturer’s specifications.

### Cell proliferation

To measure cell proliferation, the cells were seeded in 24-well plates at a density of 2.0 × 104 cells per well. The cells were cultured for 2 days and cell medium was renewed daily. Where indicated, the cells were treated with Rotenone (24 h at the indicated concentrations), then trypsinized every 24 h and quantified using the Countess automated cell counter TC10 automated cell counter (Bio-Rad). The time-course experiments were repeated three times.

### Statistics and reproducibility

The plotted data were presented as mean ± standard error of the mean (SEM). Statistical comparisons between experimental groups were determined by using one-way analysis of variance (ANOVA) followed by a Bonferroni or Dunnet post hoc test, when appropriate. When only two groups were compared, two-tailed unpaired or paired Student’s *t-*tests were used as appropriate. The normality criteria (calculated by D’Agostino and Pearson *omnibus* normally test) were met. Non-parametric data were analysed using a Kruskal–Wallis test with Dunn’s multiple comparison correction. Statistical comparisons between untreated and MitoQ-treated *mmut* zebrafish in Fig. 9c was determined by chi-square goodness of fit test. The levels of statistical significance are indicated by symbols, and the *P* values are indicated in the figure legends along with the statistical tests. All experiments reported here were performed at least two to three times independently, unless otherwise indicated in the figure legends. Experiments in Fig. 9c were performed once. The investigators were not blinded to allocation during the experiments and outcome assessment. GraphPad Prism software v. 7.0a (GraphPad software) was used for generating all statistical analyses.

### Reporting summary

Further information on research design is available in the Nature Research Reporting Summary linked to this article.

## Supplementary information

Supplementary Information

Description of Additional Supplementary Files

Supplementary Data 1

Reporting Summary

## Data Availability

The microarray data that support the findings of this study are provided in the Supplementary Data 1 and deposited in NCBI Gene Expression Omnibus (GEO), under the accession number GSE120683. The Source data underlying Fig. 1a–b, 1d, 1e, 1g, 2a–g, 3a–g, 4a–f, 5a–f, 6b–h, 7b–h, 8b–g, 9a–d, and Supplementary Figs 1a–c, 1f, 1i, 2a–e, 3a, 3c–j, 3l–m, 4c, 4e, 4g, 5b–f, 6a–b,7b, 7d–e, 7g–h, 8c–j, 9d–e, 10b, 11a–d are provided in the Source Data file. Additional data and/or reagents that support the findings of this study are available from corresponding authors, A.L. and O.D, upon reasonable request.

## Source Data

Source Data
