# Author Correction: Impaired mitophagy links mitochondrial disease to epithelial stress in methylmalonyl-CoA mutase deficiency

**DOI:** 10.1038/s41467-020-15565-6

**Published:** 2020-04-01

**Authors:** Alessandro Luciani, Anke Schumann, Marine Berquez, Zhiyong Chen, Daniela Nieri, Mario Failli, Huguette Debaix, Beatrice Paola Festa, Natsuko Tokonami, Andrea Raimondi, Alessio Cremonesi, Diego Carrella, Patrick Forny, Stefan Kölker, Francesca Diomedi Camassei, Francisca Diaz, Carlos T. Moraes, Diego Di Bernardo, Matthias R. Baumgartner, Olivier Devuyst

**Affiliations:** 1 Institute of Physiology and NCCR Kidney.CH, University of Zurich, 8057 Zurich, Switzerland; 2 Division of Metabolism and Children’s Research Center, University Children’s Hospital, 8032 Zurich, Switzerland; 3 Department of Biomedicine, University of Eastern Finland, 70211 Kuopio, Finland; 4 San Raffaele Scientific Institute, Experimental Imaging Center, 20132 Milan, Italy; 5 Division of Clinical Chemistry and Biochemistry, University Children’s Hospital Zurich, 8032 Zurich, Switzerland; 6 Telethon Institute of Genetics and Medicine, Pozzuoli, 80078 Naples, Italy; 7 Division of Inherited Metabolic Diseases, University Children’s Hospital Heidelberg, 69120 Heidelberg, Germany; 8 Department of Laboratories–Pathology Unit, Bambino Gesù Children’s Hospital, 00165 Rome, Italy; 9 Department of Neurology, University of Miami Miller School of Medicine, Miami, FL 33136 USA; 10 Division of Nephrology, Cliniques Universitaires Saint-Luc, 1040 Brussels, Belgium

**Keywords:** Mechanisms of disease, Autophagosomes, Mitochondria, Chronic kidney disease

Correction to: *Nature Communications* 10.1038/s41467-020-14729-8, published online 20 February 2020.

The original version of this Article contained an error in Fig. 3g, in which the text under the right bracket read "14 dpf-HP fed zebrafish", when it should have read "14-dpf". This has been corrected in both the PDF and HTML versions of the article.

