# Supplementary material for: Impaired mitophagy links mitochondrial disease to epithelial stress in methylmalonyl-CoA mutase deficiency
Source: Nat Commun. 2020 Feb 20;11:970. doi: 10.1038/s41467-020-14729-8 (PMC7033137; doi:10.1038/s41467-020-14729-8)
Supplement: Supplementary file 1 — Supplementary Information [file 41467_2020_14729_MOESM1_ESM.pdf]

Impaired Mitophagy Links Mitochondrial Disease to Epithelial Stress in Methylmalonyl–CoA Mutase Deficiency

Luciani A. et al.

Supplementary information

Supplementary Figure 1–13

Supplementary Table 1–4

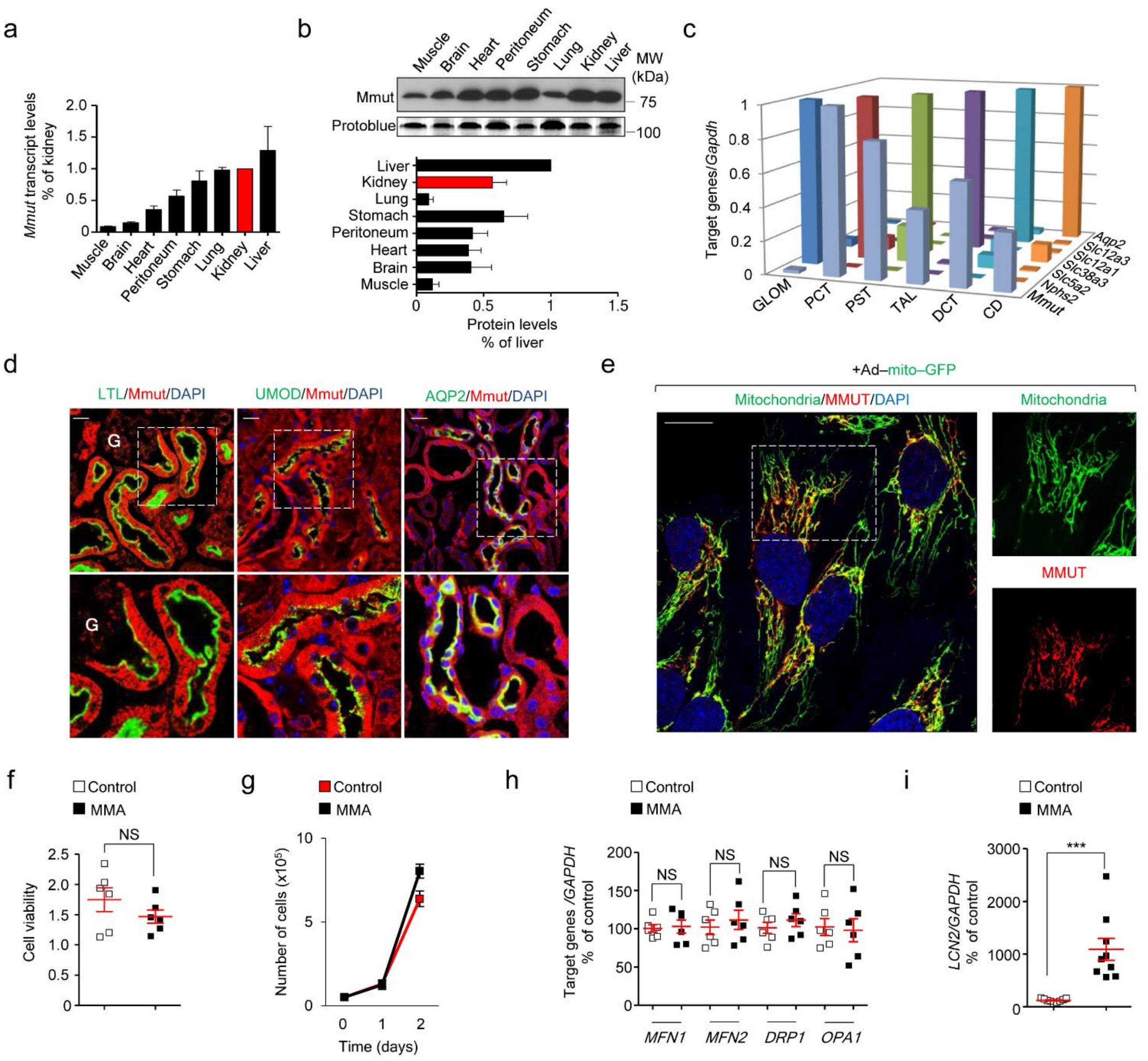

Supplementary Figure 1. Tissue, nephron–segment and intracellular compartmentalization of the enzyme MMUT, and characterization of MMA patient–derived cells. (**a**) Transcript and (**b**) protein expression of the enzyme Mmut in mouse tissues were determined by reverse transcription– quantitative PCR (RT–qPCR; *n*=3 mice) and by

immunoblotting (*n*=4 mice), respectively. Protoblue was used as loading control. (**c**) Transcript levels of *Mmut* in nephron specific segments were measured by RT–qPCR. The abundance of each segment–related gene (GLOM=*Nphs2*; PCT=*Scl5a2*; PST=*Slc38a3*; TAL=*Slc12a1*; DCT=*Slc12a3* and CD=*Aqp2*) defines the purity of each segment fraction. Quantification of all segment specific marker genes was performed in comparison with *Gapdh* as an endogenous control (*n*=3 mice). (**d**) Representative images showing the segment–specific compartmentalization of Mmut (red) in LTL^+ (green, left) proximal tubules or in $UMOD^+$ (green, middle) thick ascending limb tubules or in $AQP2^+$ (green, right) collecting duct. G denotes glomerulus. (**e**) Cells were transduced with adenoviral particles expressing the mitochondrially–targeted green fluorescent protein (Ad–mito–GFP). After 24h post–transduction, the cells were immunostained for MMUT (red) and imaged by confocal microscopy. Yellow indicates the colocalization. Dotted white squares represent regions of the respective panels magnified. **(f)** Cell viability was assessed by Cell Counting Kit–8 (CCK–8) assay; *n*=6 replicates pooled from three biologically independent experiments. (**g**) Growth curves in control and MMA kidney cells, *n*=3 biologically independent experiments. (**h–i**) Transcript levels of (**h**) *MFN1, MFN2, DRP1* and *OPA1* or (**i**) *LCN2* were measured by RT–qPCR; *n*=6 replicates in **h** and *n*=9 replicates in **i**. Values in **h** and **i** are pooled of three biologically independent experiments. Plots represent mean ± SEM. Two tailed Student's *t* test, ***$P<0.001$ relative to control cells. Nuclei counterstained with DAPI (blue) in **d** and **e**. Dotted white squares represent regions of the respective panels magnified in **d** and **e**. Scale bars are 30μm in **d** and 10μm in **e**. GLOM, glomerulus; PCT, proximal convoluted tubule; PST, proximal straight tubule; TAL, thick ascending limb tubule; DCT, distal convoluted tubule and CD, collecting duct; NS: non–significant.

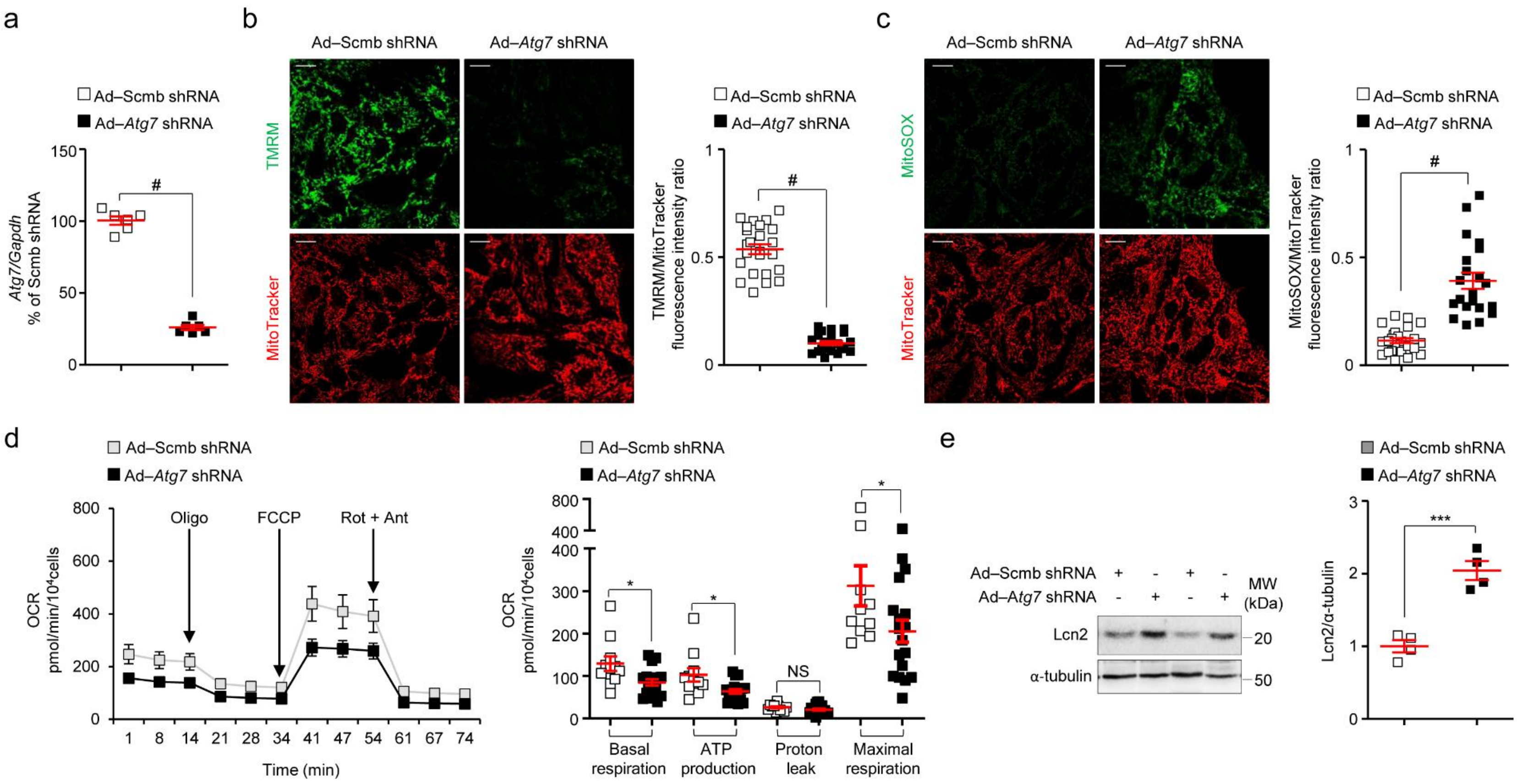

Supplementary Figure 2. Autophagy deficiency leads to mitochondrial dysfunctions in kidney tubule cells. (**a–e**) Primary cultures of proximal tubule cells derived from mouse kidneys were transduced with adenovirus particles carrying scrambled or *Atg7* short hairpin RNA (shRNA) for 5 days. (**a**) Transcript levels of *Atg7* and *Gapdh* were measured by RT–qPCR; *n*=6 replicates pooled from three biologically independent experiments. (**b–c**) Cultured cells were loaded with (**b**) tetramethylrhodamine methyl ester (TMRM, green; mitochondrial membrane potential fluorescent probe, 50 nM for 30 min at 37°C) and MitoTracker (red; fluorescent probe that localizes to mitochondria, 1 μM for 30 min at 37°C) or with (**c**) MitoSOX (green; mitochondrial ROS indicator, 2.5 μM for 30 min at 37°C) and MitoTracker (red), and analyzed by confocal microscopy. Representative images and quantification of (**b**) membrane potential and (**c**) mitochondrial ROS production (both calculated as ratio between TMRM and MitoTracker or MitoSOX and MitoTracker fluorescence intensities, with each point representing the average fluorescence intensity ratio in a cell). TMRM/MitoTracker: *n*=24 control cells and *n*=23 *Atg7*-deleted cells. MitoSOX/MitoTracker: *n*=24 control cells and *n*=21 *Atg7*-deleted cells. Values are pooled from three biologically independent experiments. (**d**) Oxygen consumption rate (OCR) and individual parameters for basal respiration, ATP production, proton leak and maximal respiration. OCRs were measured at baseline and after the sequential addition of Oligomycin (Oligo, 1μM), FCCP (0.5μM) and Rotenone (Rot; 1μM) + Antimycin A (Ant; 1μM); *n*=11 replicates pooled from four biologically independent experiments (for cells transduced with Ad–Scmb shRNA) and *n*=18 replicates pooled from six biologically independent experiments (for cells transduced with

Ad–*Atg7* shRNA). (**e**) Immunoblotting and quantification of Lcn2, *n*=4 biologically independent experiments. α–tubulin was used as loading control. Plots represent mean ± SEM. Two tailed Student's *t* test, $^{*}P<0.05$, $^{***}P<0.001$ and $^{\#}P<0.0001$ relative to cells transduced with Scmb shRNA. Scale bars, 10µm. NS: non–significant.

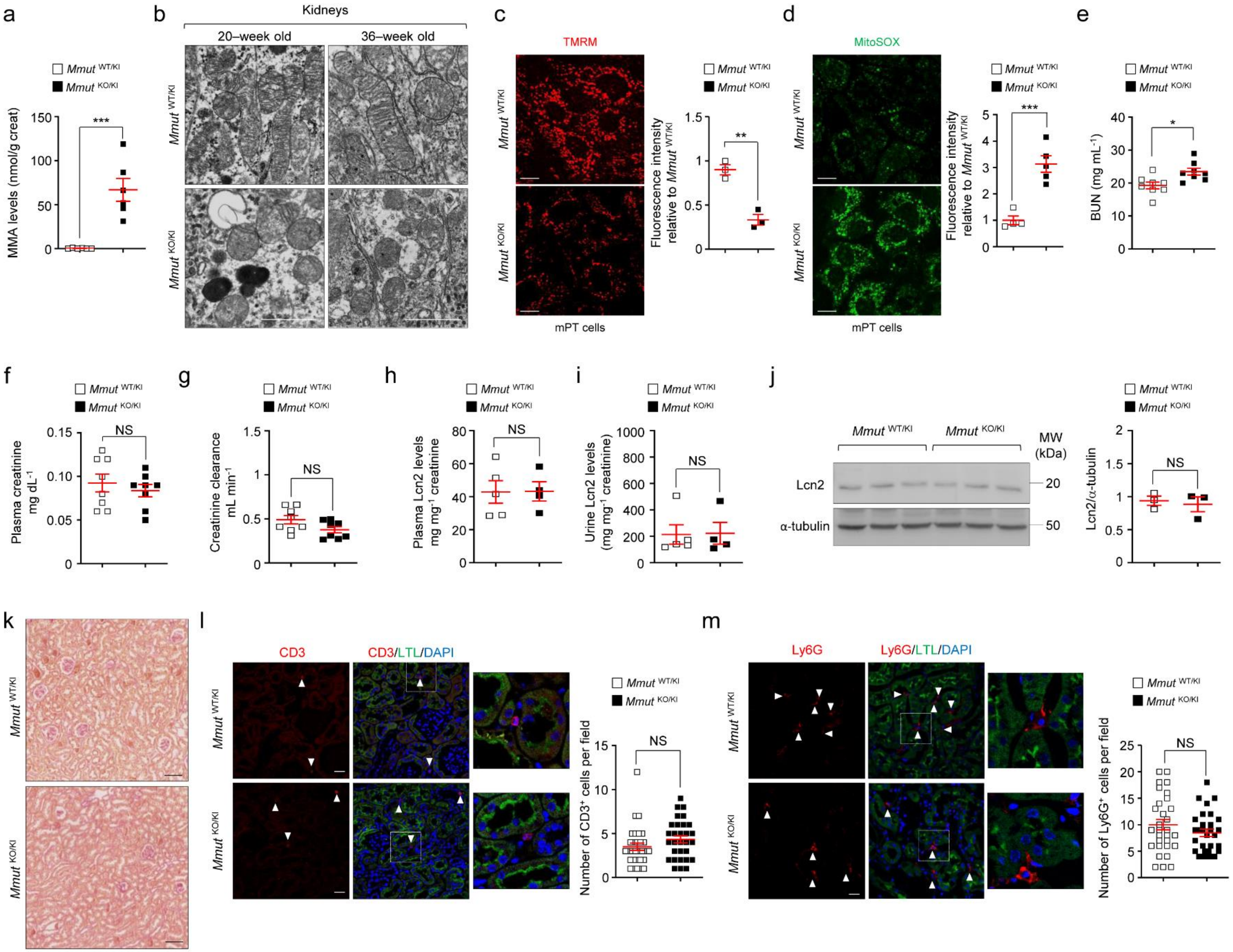

Supplementary Figure 3. Mitochondrial alterations and kidney phenotypes in *Mmut* $^{KO/KI}$ mice. (**a**) Quantification of MMA levels in the urines of 32–36– week–old *Mmut*$^{WT/KI}$ and *Mmut*$^{KO/KI}$ by LC–MS/MS; *n*=6 mice per each group. (**b**) Representative electron micrographs showing the mitochondrial network in 20 or 36–week–old kidney

tubules of *Mmut*$^{WT/KI}$ and *Mmut*$^{KO/KI}$ mice. (**c–d**) Primary cultures of proximal tubule cells derived from 20 week–old kidneys of *Mmut* $^{WT/KI}$ and *Mmut* $^{KO/KI}$ mice were loaded (**c**) with tetramethylrhodamine methyl ester (TMRM; green; mitochondrial membrane potential fluorescent probe, 50 nM for 30 min at 37°C) and MitoTracker (red; fluorescent probe that localizes to mitochondria; 1 μM for 30 min at 37°C) or with (**d**) MitoSOX (green; mitochondrial ROS indicator, 2.5 μM for 30 min at 37°C) and MitoTracker (red), and analyzed by confocal microscopy. Representative images and quantification of TMRM or MitoSOX fluorescence intensities; n=3 randomly selected and non–overlapping fields of views (for TMRM) and $n \geq 4$ randomly selected and non–overlapping fields of views (for MitoSOX). Each whole–field image contains at least 10 cells. The whole–field images used are pooled from three biologically independent experiments. (**e–f**) Quantification of (**e**) urea or (**f**) creatinine, and (**g**) measurement of creatinine clearance in 32–36 –week–old *Mmut* mice (n=8 mice per each group). (**h–i**) Protein abundance of Lcn2 (**h**) in plasma or (**i**) in the urines collected from 32–36– week–old *Mmut* mice was measured by ELISA, n= 5 *Mmut*$^{WT/KI}$ and n= 4 *Mmut*$^{KO/KI}$ mice, respectively. (**j**) Immunoblotting and quantification of Lcn2 in whole–tissue lysates harvested from 32–36 –week–old *Mmut* mouse kidneys (n=3 mouse kidney samples per each group). α–tubulin was used as loading control. (**k**) Representative micrographs and detection of collagen deposition by Picro Sirius Red staining in the kidneys of 32–36 –week–old *Mmut* mice. (**l–m**) Representative images and quantification of numbers of (**l**) CD3^{+} cells (T cell marker; red) or (**m**) Ly6G^{+} (marker of granulocytes, monocytes and neutrophils; red) in the kidneys of 32–36– week–old *Mmut* mice. Proximal tubules were labelled by Lotus Tetragonolobus Lectin (LTL; green); n=30 randomly selected and non–overlapping fields of views. The whole–field images are pooled from three independent mouse kidney samples. Dotted white squares represent regions of the respective panels magnified. Arrowheads indicate either CD3 (in **l**) or Ly6G (in **m**) positive cells. Plots represent mean ± SEM. Two tailed Student's t test, *P<0.05, **P<0.01 and ***P<0.001 relative to *Mmut*$^{WT/KI}$. Nuclei counterstained with DAPI (blue). Scale bars are 1μm in **b**, 10 μm in **c** and **d**, 1mm in **k** and 20μm in **l** and **m**. NS: non–significant.

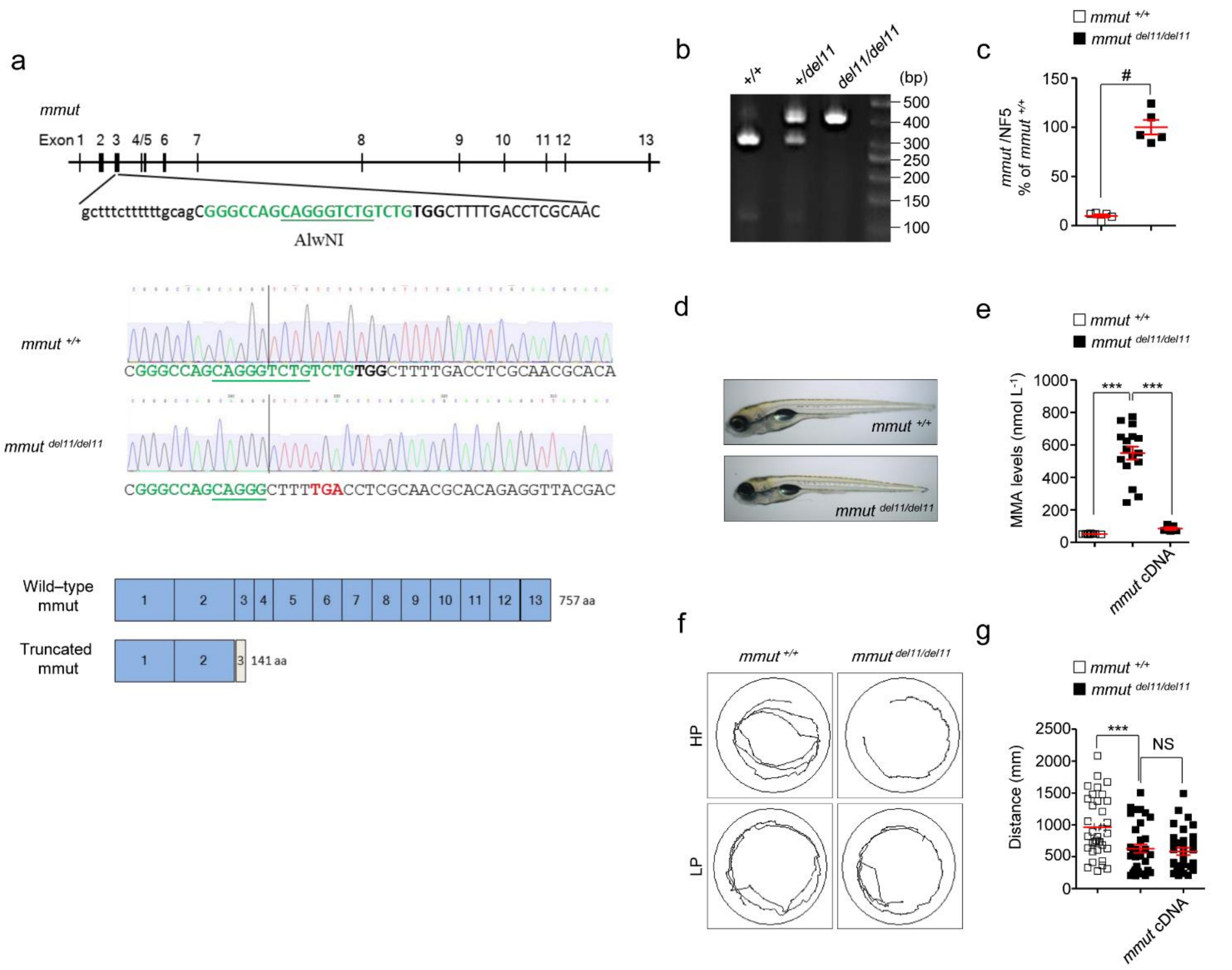

Supplementary Figure 4. Generation of *mmut* knockout zebrafish model. (**a**) CRISPR–Cas9–induced deletion (green) generates a frameshift of the open reading frame resulting in a premature stop codon (TGA) within the exon 3 of *mmut* gene (middle panel) that produces a truncated protein of 141 amino acids. The underlined sequence represents the AlwNI site used for the detection of the deletion. (**b**) AlwNI digestion of PCR products after the amplification of the CRISPR–Cas9 targeting region using genomic DNA extracted from caudal of wild–type (+/+), heterozygous (+/del11) and homozygous (del11/del11) zebrafish. Wild type allele: two lower bands (that are, 333bp + 120bp, respectively) correspond to PCR products cut by AlwNI. Mutant allele: upper band 453bp, which is resistant to AlwNI digestion. (**c**) Transcript levels of *mmut* in zebrafish kidneys were determined by RT–qPCR; *n*=5 zebrafish larvae per each group. (**d**) Representative images of *mmut*$^{+/+}$and *mmut*$^{\mathrm{del11/del11}}$zebrafish embryos at 5–dpf. (**e**) Quantification of MMA levels by LC–MS/MS; *n*=8 *mmut*$^{+/+}$, *n*=16 *mmut*$^{\mathrm{del11/del11}}$ and *n*=5 *mmut*$^{\mathrm{del11/del11}}$ zebrafish expressing *mmut* in the liver. (**f**) Tracking analyses of motor behavior in 10–dpf–*mmut* zebrafish larvae fed with high or low protein diet. (**g**) Quantification of mean distance, with each point representing the mean distance covered by an

individual zebrafish; n=36 *mmut*$^{+/+}$, n=34 *mmut*$^{del11/del11}$ and n=33 *mmut*$^{del11/del11}$ zebrafish larvae expressing *mmut* cDNA in the liver. Plots represent mean ± SEM. Two tailed Student's *t* test, $^{\#}P$<0.0001 relative to *mmut* $^{+/+}$ in **c**. One–way ANOVA followed by Bonferroni's *post hoc* test, ***P<0.001 relative to *mmut* $^{+/+}$ or to *mmut*$^{del11/del11}$ zebrafish larvae in **e** and **g**. NS: non–significant.

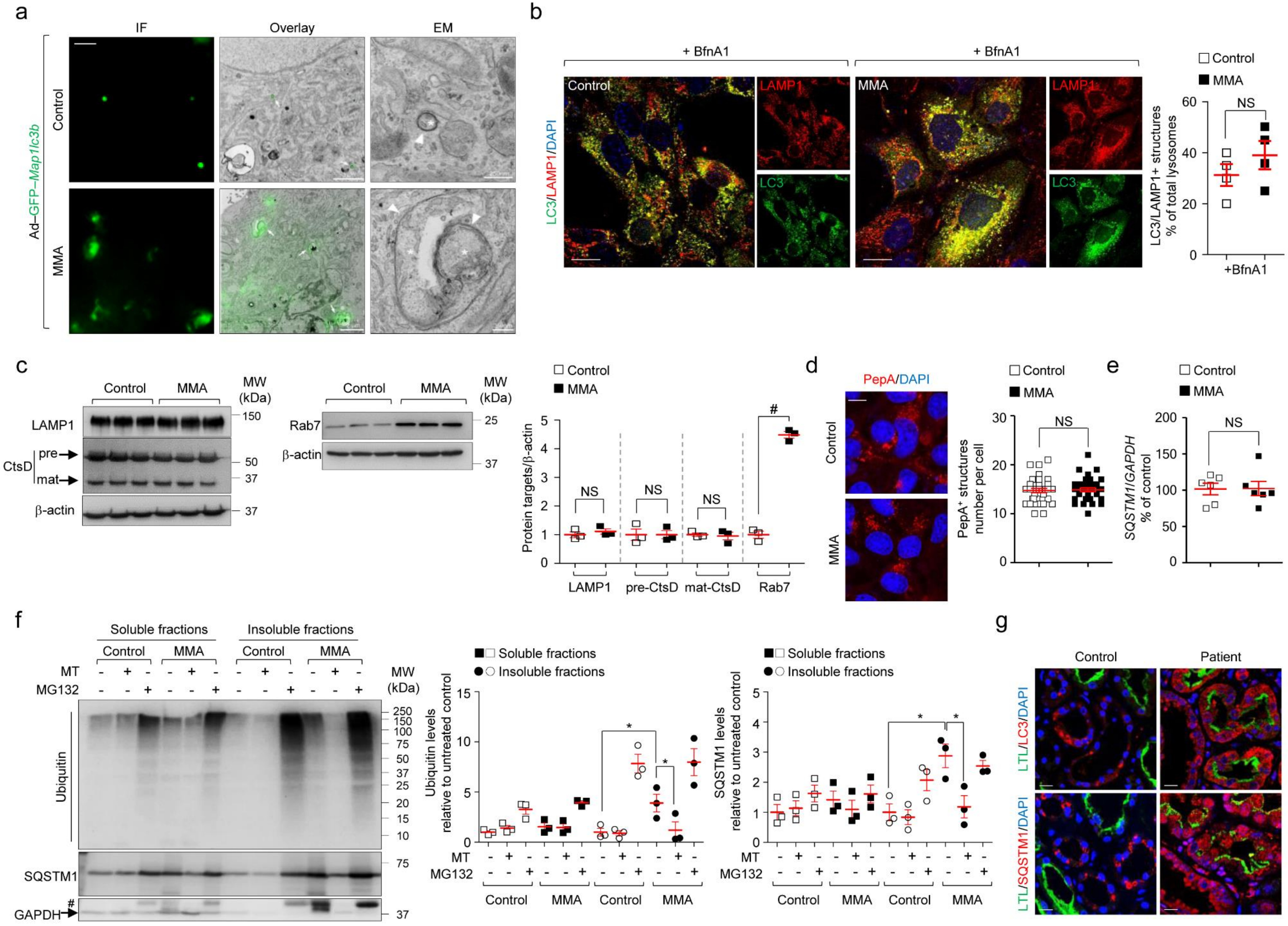
a
IF
Overlay
EM
Ad–GFP–Map1lc3b
Control
MMA
b
+ BfnA1
+ BfnA1
LC3/LAMP1/DAPI
Control
LAMP1
LC3
MMA
LAMP1
LC3
Control
MMA
NS
LC3/LAMP1+ structures
% of total lysosomes
+BfnA1
c
Control
MMA
MW
(kDa)
LAMP1
CtsD
pre
mat
β-actin
150
50
37
37
Control
MMA
MW
(kDa)
Rab7
β-actin
25
37
Control
MMA
NS
NS
NS
#
Protein targets/β-actin
LAMP1
pre-CtsD
mat-CtsD
Rab7
d
PepA/DAPI
Control
MMA
Control
MMA
NS
PepA+ structures
number per cell
e
Control
MMA
NS
SQSTM1/GAPDH
% of control
f
Soluble fractions
Insoluble fractions
Control
MMA
Control
MMA
MT
MG132
MW
(kDa)
Ubiquitin
SQSTM1
GAPDH
#
Soluble fractions
Insoluble fractions
Ubiquitin levels
relative to untreated control
MT
MG132
Control
MMA
Control
MMA
Soluble fractions
Insoluble fractions
SQSTM1 levels
relative to untreated control
MT
MG132
Control
MMA
Control
MMA
g
Control
Patient
LTL/LC3/DAPI
LTL/SQSTM1/DAPI

Supplementary Figure 5. Dysregulation of autophagy in MMA cells. (**a**) Cells were transduced with adenoviral particles carrying GFP–tagged *Map1lc3b* (Ad–GFP–*Map1lc3b*) for 24h. The punctate GFP–LC3$^+$ structures were imaged by confocal microscopy. Randomly selected cells were further processed, and serial sections were analyzed by transmission electron microscopy. (**b**) Cells were cultured in presence or in absence of Bafilomycin A1 (BfnA1, 250 nM for 4h). Representative images and quantification of numbers of LC3/LAMP1$^+$ structures (expressed as percentage of total lysosomes; *n*= 4 randomly selected and non–overlapping fields of views per each condition. Each whole–field image contains at least 10 cells. The whole–field images are pooled from two biologically independent experiments. Yellow indicates colocalization. (**c**) Immunoblotting and quantification of LAMP1, Cathepsin D (CtsD) and Rab7; β–actin was used as loading control. Two tailed Student's *t*–test, $^{\#}P<0.0001$ relative to control cells, *n*=3 biologically independent experiments. (**d**) Cells were loaded Bodipy–FL–Pep A (1 µM, for 1h at 37°C), fixed and imaged by confocal microscopy. Quantification of numbers of Pep A$^+$ structures in a cell; *n*= 30 cells pooled from three biologically independent experiments. (**e**) Transcript levels of *SQSTM1* and *GAPDH* in control and MMA cells were measured by RT–qPCR; *n*=6 replicates pooled from three biologically independent experiments. (**f**) Control and MMA cells were treated with the mitochondria–targeted ROS scavenger mito–TEMPO (MT, 10 µm for 24h) or with the proteasome inhibitor MG132 (50 µm for 16h). Representative immunoblotting and quantification of ubiquitin, SQSTM1 and GAPDH in the soluble and insoluble fractions obtained from control and MMA cells (*n*=3 biologically independent experiments). Two tailed Student's *t*–test, $^{*}P<0.05$ relative to untreated control or MMA cells. Asterisk refers to non–specific bands. (**g**) Representative images of LC3 (top, red) and SQSTM1 (bottom, red) in kidney biopsy samples from an individual healthy control subject and patient with MMA. Kidney proximal tubules labelled by LTL (green). Nuclei counterstained with DAPI. Scale bars are 10µm (left) and 2µm (middle) and 250nm (right) in **a,** 10µm in **b** and **d,** and 30µm in **g**. NS: non–significant.

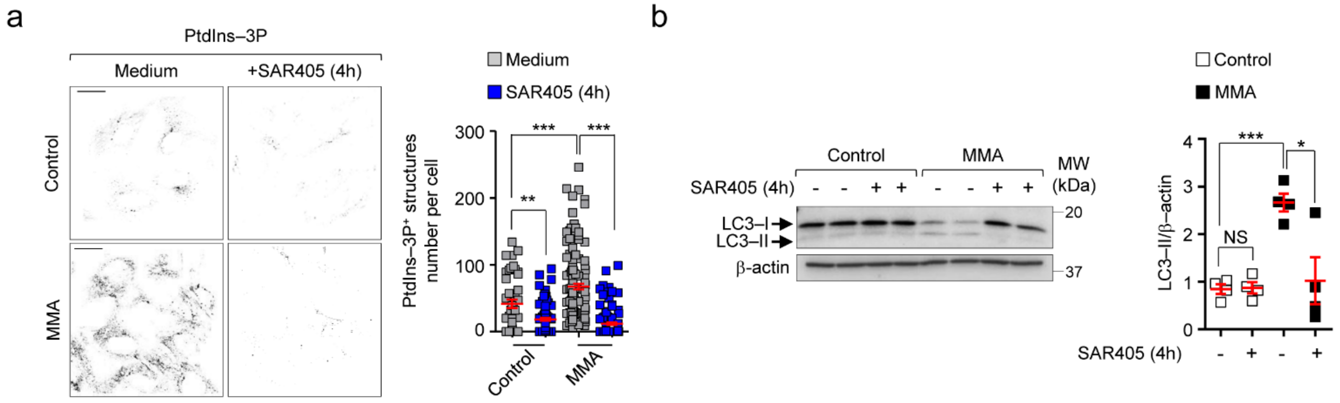

Supplementary Figure 6. Inhibition of Vps34–induced PtdIns–3P production blocks autophagosome biogenesis in control and MMA kidney cells. (a–b) Cells were exposed to the highly selective Vps34 inhibitor SAR405 (5 µM for 4h). (**a**) Representative inverted images and quantification of numbers of PdtIns–3P$^+$ structures in a cell; *n*=45 untreated control cells, *n*= 93 SAR405–treated control cells, *n*=141 untreated MMA cells and *n*=114 SAR405–treated MMA cells. Values are pooled from three biologically independent experiments. One–way ANOVA followed by Bonferroni's *post hoc* test, **P<0.01 and ***P<0.001 relative to untreated control or MMA cells. (**b**) Representative immunoblotting and quantification of LC3, *n*=4 biologically independent experiments. β–actin was used as a loading control. Two tailed Student's *t* test, *P<0.05 and ***P<0.001 relative to untreated control or MMA cells. NS: non–significant.

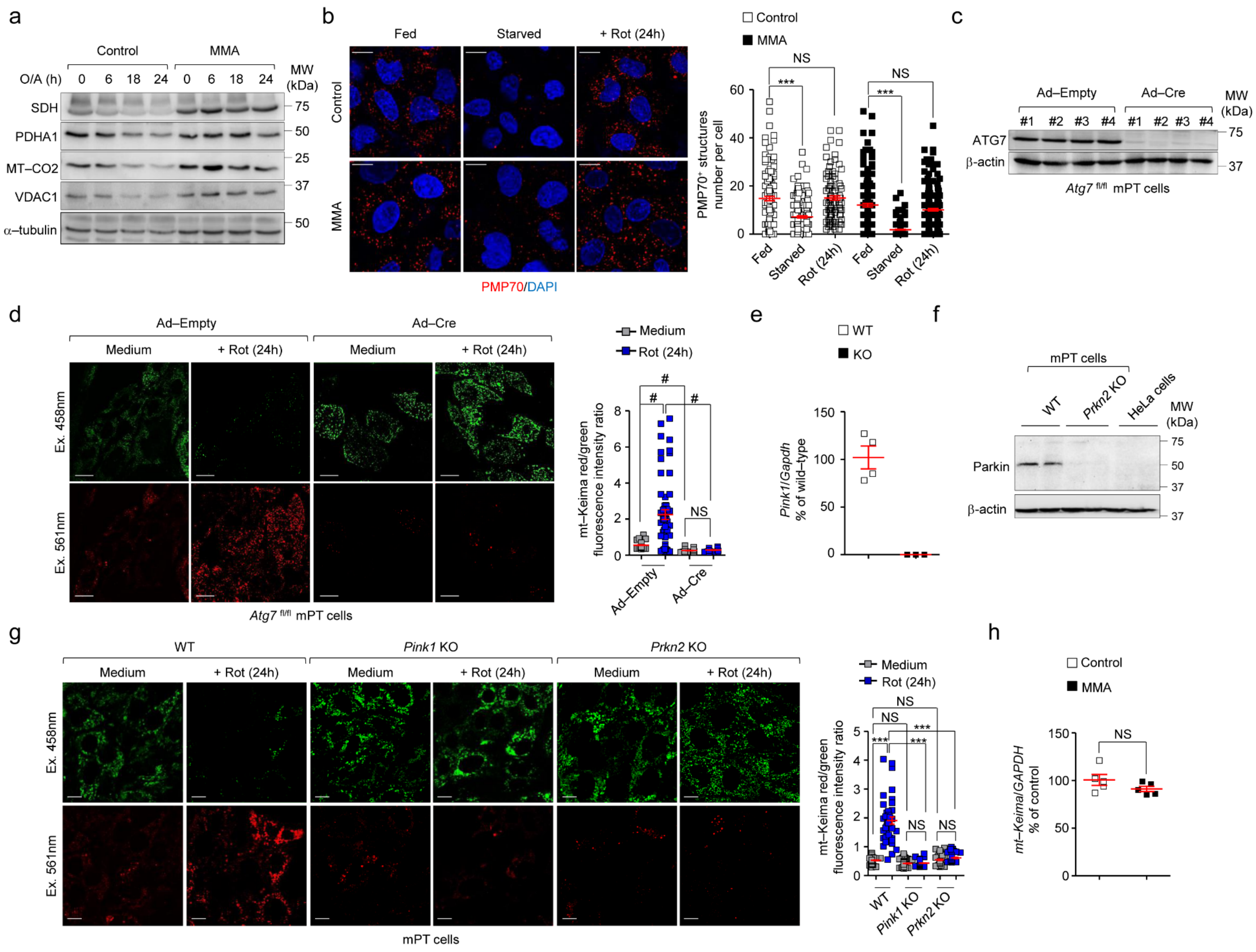
a
Control
MMA
O/A (h) 0 6 18 24 0 6 18 24
MW (kDa)
SDH
PDHA1
MT–CO2
VDAC1
α–tubulin
75
50
25
37
50
b
Fed
Starved
+ Rot (24h)
Control
MMA
PMP70/DAPI
Control
MMA
NS
***
PMP70⁺ structures number per cell
Fed
Starved
Rot (24h)
c
Ad–Empty
Ad–Cre
#1 #2 #3 #4 #1 #2 #3 #4
ATG7
β-actin
Atg7 fl/fl mPT cells
d
Ad–Empty
Ad–Cre
Medium
+ Rot (24h)
Ex. 458nm
Ex. 561nm
Atg7 fl/fl mPT cells
Medium
Rot (24h)
mt–Keima red/green fluorescence intensity ratio
#
NS
Ad–Empty
Ad–Cre
e
WT
KO
Pink1/Gapdh % of wild–type
f
mPT cells
WT
Prkn2 KO
HeLa cells
Parkin
β-actin
g
WT
Pink1 KO
Prkn2 KO
Medium
+ Rot (24h)
Ex. 458nm
Ex. 561nm
mPT cells
Medium
Rot (24h)
NS
***
mt–Keima red/green fluorescence intensity ratio
h
Control
MMA
NS
mt–Keima/GAPDH % of control

Supplementary Figure 7. Mitophagy in MMA cells or in proximal tubule cells derived from floxed *Atg7* or from *Pink1* or *Prkn2* knockout mice. (**a**) Cells were exposed to Oligomycin (4 μM) and Antimycin (0.8 μM) for the indicated times. Immunoblotting of the indicated mitochondrial proteins. The experiments were repeated twice with similar results. (**b**) Cells were cultured under normal growth (fed) or nutrient–deprived (starved) conditions or exposed to Rotenone (Rot, 5 μM for 24h) and immunostained for PMP70 (red), and analysed by confocal microscopy. Representative images and quantification of $PMP70^+$ structures in a cell; *n*= 142 fed control cells, *n*=181 starved control cells and *n*=108 Rot–treated control cells, *n*=189 fed MMA cells, *n*=196 starved MMA cells and *n*=212 Rot–treated MMA cells. Values are pooled from three biologically independent experiments. One–way ANOVA followed by Bonferroni's *post hoc* test, $^{***}P<0.001$ relative to fed control or fed MMA cells. Nuclei counterstained with DAPI (blue). (**c–d**) Primary cultures of proximal tubule cells derived from kidneys of floxed *Atg7* mice were transduced adenovirus particles bearing Empty (Ad–Empty) or Cre recombinase (Ad–Cre) for 5 days. (**c**) Validation of the deletion of *Atg7* by immunoblotting, *n*=4 biologically independent experiments. (**d**) Control and *Atg7*–deleted cells were transduced with adenoviral particles carrying the mitochondrially–targeted form of Keima (mt–Keima) for 24h, and subsequently exposed to Rotenone (5 μM) for 24h. Representative images and quantification of the ratio between red and green fluorescence intensities, with each point representing the average red/green fluorescence intensity ratio in a cell; *n*=54 untreated control cells, *n*=55 Rot–treated control cells, *n*=46 untreated *Atg7* and *n*=44 Rot–treated *Atg7* deleted cells. Values are pooled from three biologically independent experiments. Two tailed Student's *t* test, $^{\#}P<0.0001$ relative to untreated or Rot–treated control cells**.** (**e**) Transcript levels of *Pink1* and *Gapdh* were determined by RT–qPCR in 12–week–old brain of *Pink1* WT and KO mice; *n*=4 wild type and *n*=3 knockout brains. (**f**) Immunoblotting of Parkin in *Prkn2* wild type and knockout kidneys or in HeLa cells. (**g**) Wild type, *Pink1* and *Prkn2* knockout cells were transduced with adenoviral particles bearing the mitochondrially–targeted form of Keima (mt–Keima). After 24h post–transduction, mt–Keima expressing cells were exposed to Rotenone for 24h and analysed by confocal microscopy. Representative images and quantification of the ratio between red and green fluorescence intensities, with each point representing the average red/green fluorescence intensity ratio in a cell; *n*=50 untreated wild type cells and *n*=41 Rot–treated wild type cells, *n*=82 untreated *Pink1* knockout cells and *n*=79 Rot–treated *Pink1* knockout cells, and *n*=55 untreated *Prkn2* knockout cells and *n*=44 Rot–treated *Prkn2* knockout cells. Values are pooled from three biologically independent experiments. One–way ANOVA followed by Bonferroni's *post hoc* test, $^{***}P<0.001$ relative to untreated or Rotenone–treated wild type cells. (**h**) Transcript levels of *mt–Keima* and *GAPDH* in control and MMA cells were measured by RT–qPCR; *n*=5 biologically independent experiments. α–tubulin and β–actin was used as loading control in **a** and in **b**, respectively. Plots represent mean ± SEM. Scale bars are 10μm. NS: non–significant.

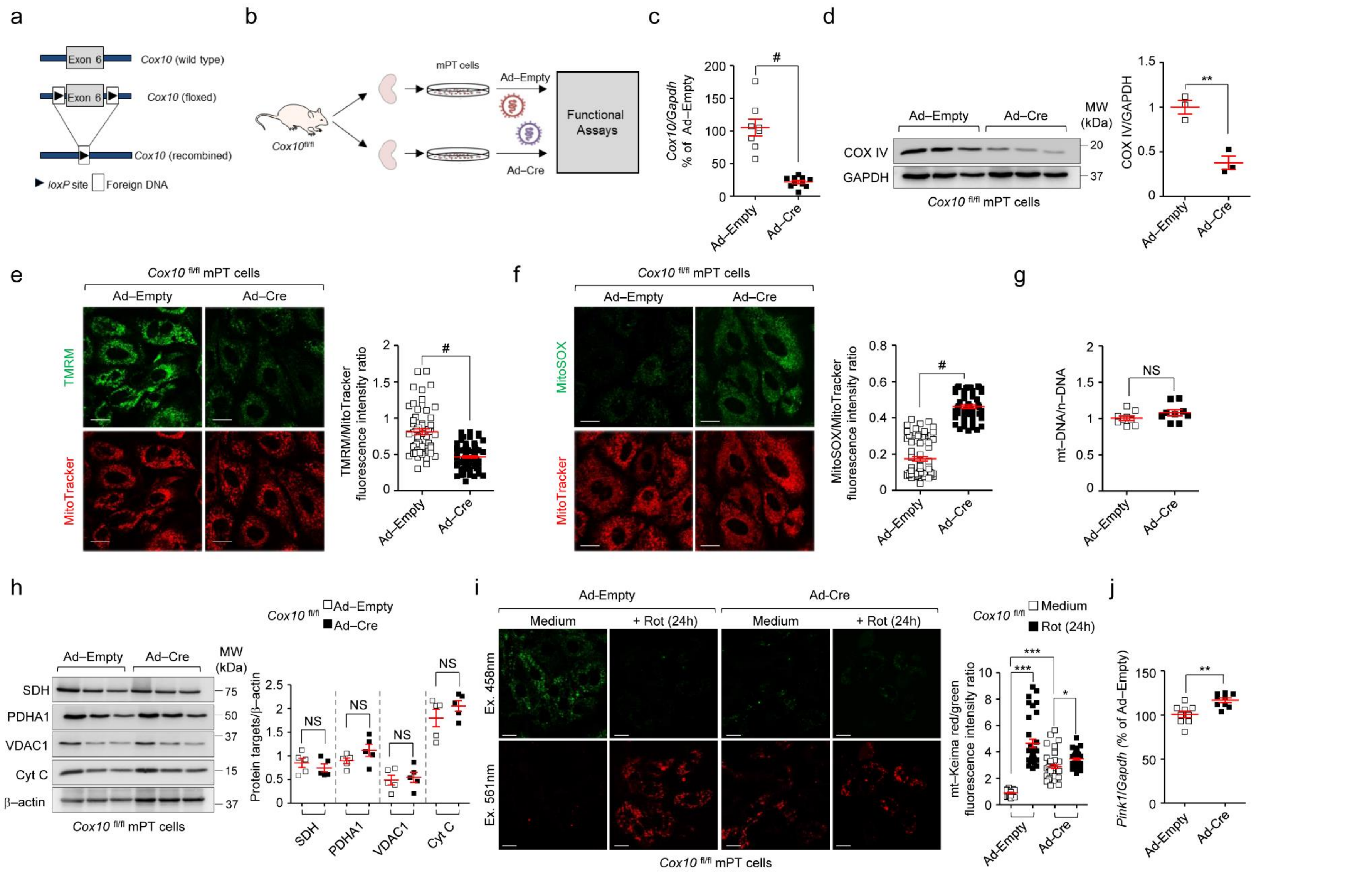

Supplementary Figure 8. Mitochondrial abnormalities in *Cox10*–deficient kidney tubular cells are not linked to deficient mitophagy. (**a–j**) Primary cultures of proximal tubule cells derived from kidneys of floxed *Cox10* mice were transduced with adenoviral particles carrying Empty (Ad–Empty) or Cre recombinase (Ad–Cre) for 5 days. (**a–b**) Workflow of the strategy used (**a**) to generate floxed *Cox10* alleles and (**b**) to delete the *Cox10* gene *in vitro*. (**c–d**) Validation of *Cox10* deletion (**c**) by RT–qPCR ($n \geq 8$ replicates pooled from four biologically independent experiments) and (**d**) by immunoblotting, n=3 biologically independent experiments. (**e–f**) Cultured cells were loaded (**e**) with

tetramethylrhodamine methyl ester (TMRM; green; mitochondrial membrane potential fluorescent probe, 50 nM for 30 min at 37°C) and MitoTracker (red; fluorescent probe that localizes to mitochondria; 1μM for 30 min at 37°C) or (**f**) with MitoSOX (green; mitochondrial ROS indicator, 2.5 μM for 30 min at 37°C) and MitoTracker (red), and analysed by confocal microscopy. Representative images and quantification of (**e**) membrane potential and (**f**) mitochondrial ROS production (both calculated as ratio between TMRM and MitoTracker or MitoSOX and MitoTracker fluorescence intensities, with each point representing the average fluorescence intensity ratio in a cell). TMRM/MitoTracker: *n*=60 cells transduced with Ad–Empty and *n*=95 cells transduced with Ad–Cre. MitoSOX/MitoTracker: *n*=74 cells transduced with Ad–Empty and *n*=56 cells transduced with Ad–Cre. Values are pooled from three biologically independent experiments. (**g**) Ratio between mitochondrial DNA (*Nd1*) and nuclear DNA (*Hbb*) was determined by quantitative PCR; *n*=9 replicates pooled from three biologically independent experiments. (**h**) Immunoblotting and quantification of the indicated mitochondrial proteins; *n*=5 biologically independent experiments. (**i**) Control and *Cox10* deleted cells were transduced with adenoviral particles bearing the mitochondrially–targeted form of Keima (mt–Keima) for 24h and subsequently exposed to Rotenone (5μM) for 24h. Representative images and quantification of ratio between red and green fluorescence intensities, with each point representing the average red/green fluorescence intensity ratio in a cell; *n*=39 untreated control cells, *n*=35 Rot–treated control cells, *n*=40 untreated *Cox10* deleted cells and *n*=45 Rot–treated *Cox10* deleted cells. Values are pooled from three biologically independent experiments. One–way ANOVA followed by Bonferroni's *post hoc* test, $^*P<0.05$ and $^{***}P<0.001$ relative to untreated control cells or untreated *Cox10*–deleted cells. (**j**) Transcript levels of *Pink1* and *Gapdh* in *Cox10* cells were determined by RT–qPCR, *n*= 9 replicates pooled from three biologically independent experiments. Two tailed Student's *t*–test, $^{**}P<0.01$ and $^{\#}P<0.0001$ relative to cells transduced with Ad–Empty in **c**, **d**, **e**, **f** and **j**. Plots represent mean ± SEM. Scale bars are 10μm. NS: non–significant.

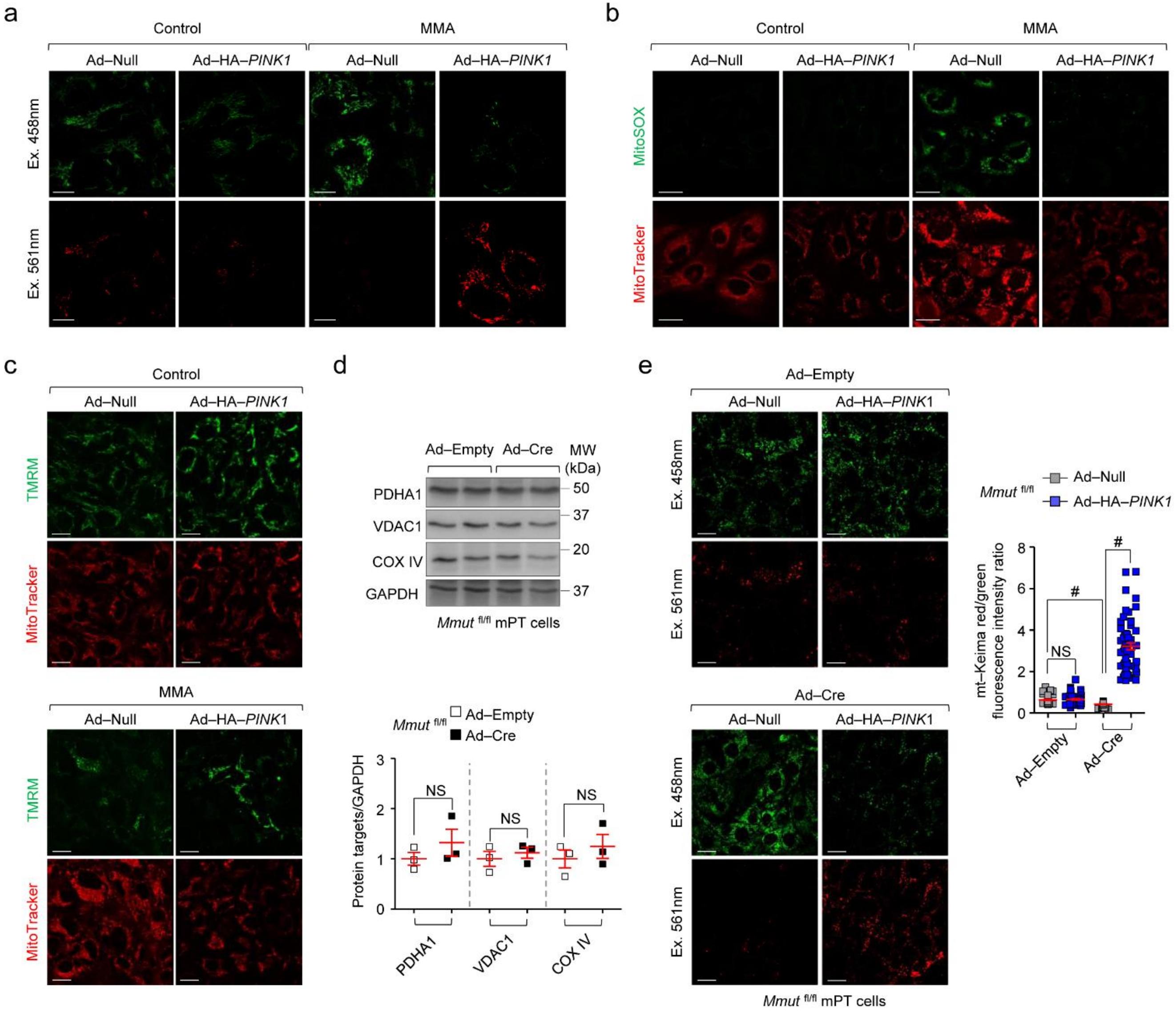

Supplementary Figure 9. Re–expression of PINK1 restores mitophagy in patient–derived and *Mmut* deleted kidney tubular cells. (**a–c**) Representative images of experiments shown in figure 6**d**, 6**f** and 6**g**. (**d**) Immunoblotting and quantification of the indicated mitochondrial proteins; *n*=3 biologically independent experiments.(**e**) Control and *Mmut* deleted cells expressing Ad–Null or Ad–HA–*PINK1* were transduced with adenoviral particles bearing the mitochondrially–targeted form of Keima (mt–Keima) for 24h, and analyzed by confocal microscopy. Representative images and quantification of ratio between red and green fluorescence intensities, with each point representing the

average red/green fluorescence intensity ratio in a cell; *n*= 57 control cells transduced with Ad–Null and *n*= 65 control cells transduced with Ad–HA–*PINK1*, and *n*= 55 *Mmut* deleted cells transduced with Ad–Null; *n*= 59 *Mmut* deleted cells transduced with Ad–HA–*PINK1*. Values are pooled from three biologically independent experiments. Two tailed Student's *t*–test, $^{\#}P$<0.0001 relative to control cells transduced with Ad–Null or to *Mmut* deleted cells transduced with Ad–Empty. Plots represent mean ± SEM. Scale bars are 10 μm.

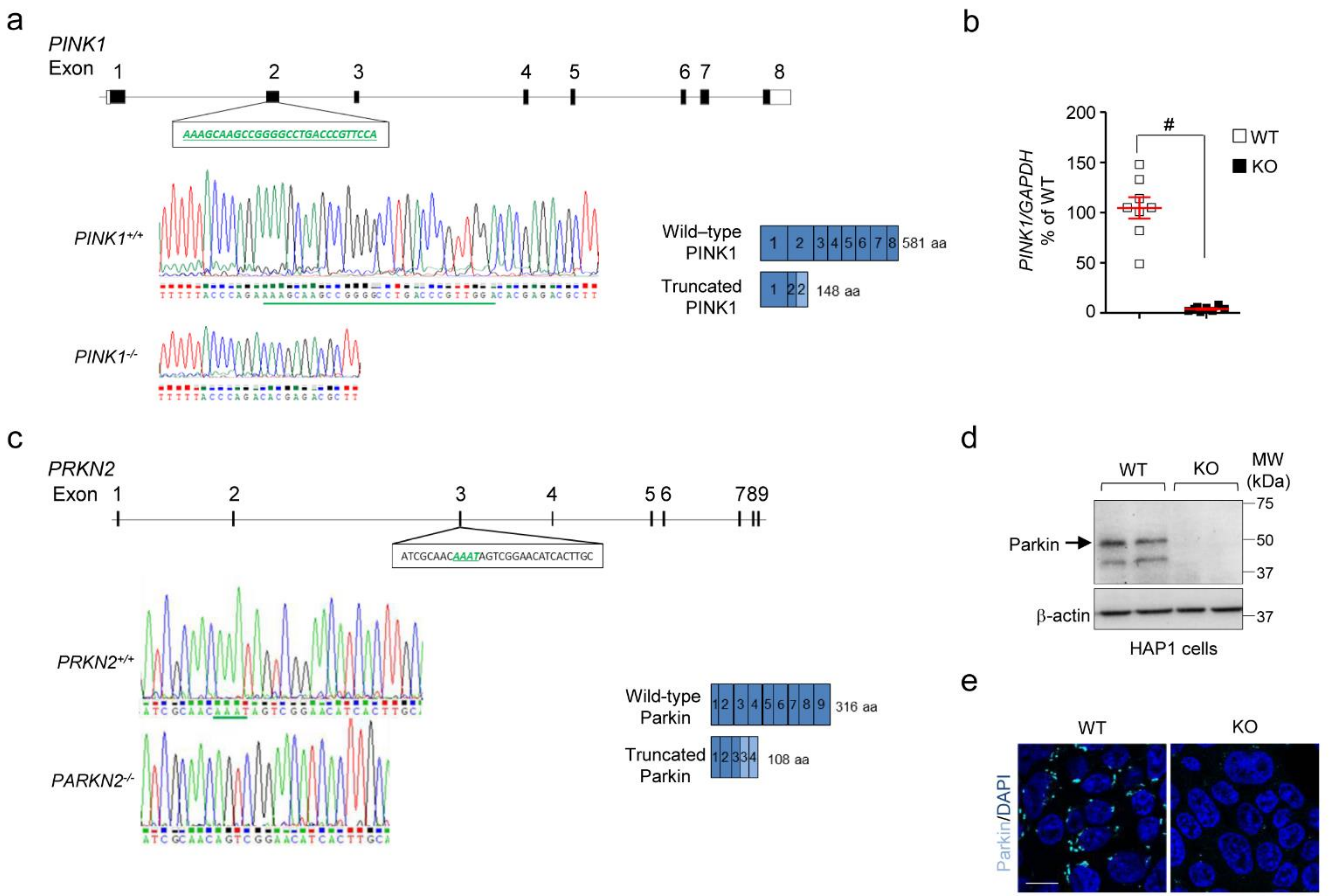

Supplementary Figure 10. Generation of *PINK1* and *PRKN2* knockout in HAP–1 cells. (**a**) CRISPR–Cas9–induced deletion (green) generates a frameshift of the open reading frame, resulting in premature stop codon (TGA) within the exon 2 of *PINK1* gene, which ultimately produces a truncated protein of 148 amino acids. (**b**) Validation of the *PINK1* deficiency by RT–qPCR, n=8 replicates pooled from four biologically independent experiments. Plots represent mean ± SEM. Two tailed Student's t–test, $^{\#}P$<0.0001 relative to wild type cells. (**c**) CRISPR–Cas9–induced deletion (green) generates a frameshift of the open reading frame, resulting in premature stop codon (TGA) within the exon 3 of *PRKN2* gene, which ultimately produces a truncated protein of 108 amino acids. (**d–e**) Validation of the *PRKN2* deficiency by (**d**) immunoblotting and (**e**) confocal microscopy assays. Arrowhead indicates the predicted molecular weight for Parkin. Nuclei counterstained with DAPI (blue). Scale bars, 10µm.

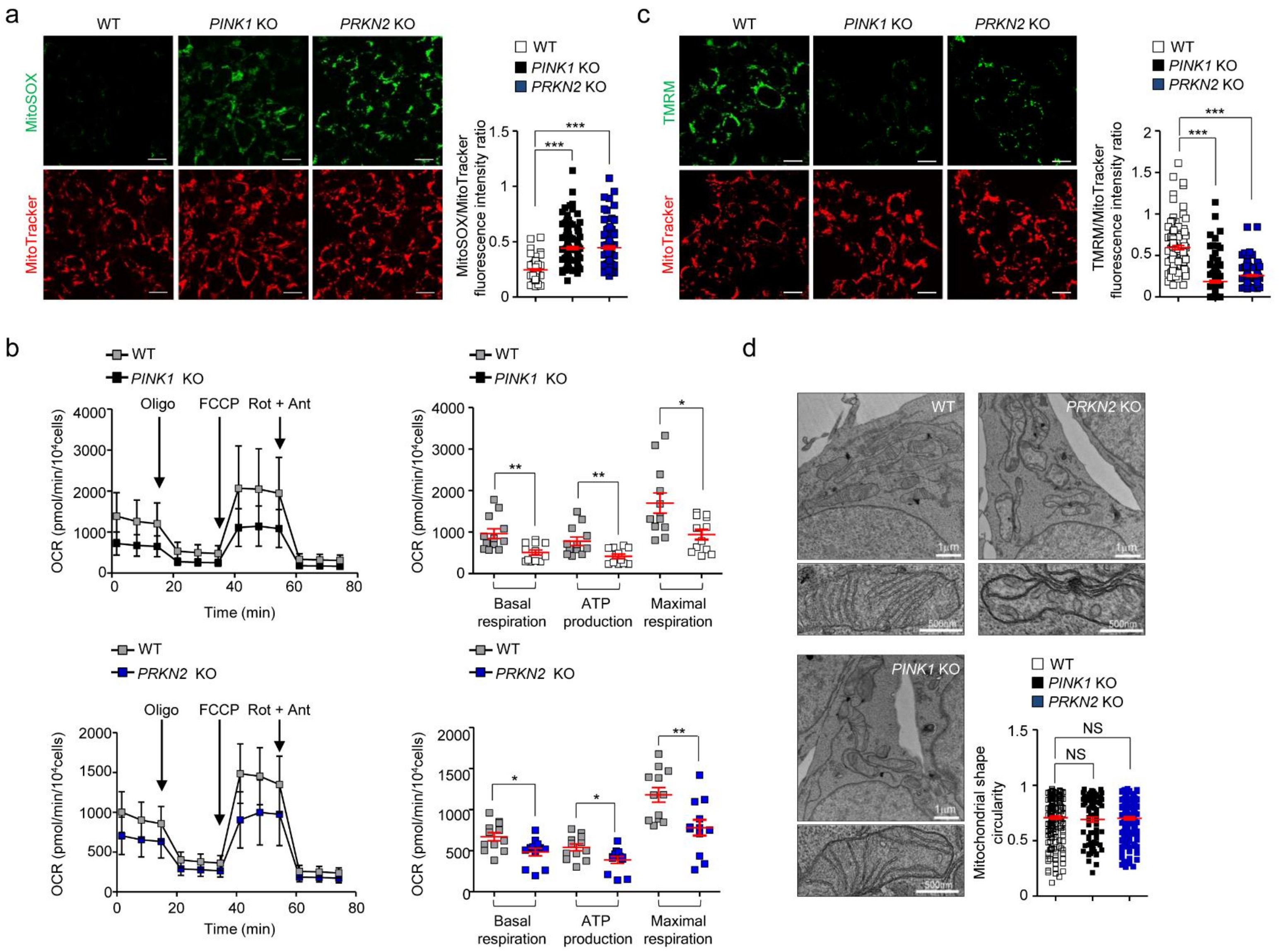

Supplementary Figure 11. ***PINK1* and *PRKN2* deficiencies lead to mitochondrial alterations in HAP–1 cells.** (**a** and **c**) Cells were loaded (**a**) with MitoSOX (green; mitochondrial ROS indicator, 2.5 µM for 30 min at 37°C) and MitoTracker (red; fluorescent probe that localizes to mitochondria; 1µM for 30 min at 37°C) or with tetramethylrhodamine methyl ester (TMRM; green; mitochondrial membrane potential fluorescent probe, 50 nM for 30 min at 37°C) and MitoTracker (red), and analysed by confocal microscopy. Representative images and quantification of (**a**) mitochondrial ROS production and (**c**) membrane potential (both calculated as ratio between TMRM and

MitoTracker or MitoSOX and MitoTracker fluorescence intensities, with each point representing the average fluorescence intensity ratio in a cell). MitoSOX/MitoTracker: *n*=93 wild type cells, *n*=143 *PINK1* knockout cells and *n*=113 *PRKN2* knockout cells in **a**; TMRM/MitoTracker: *n*=114 wild type cells, *n*=141 *PINK1* knockout cells and *n*=147 *PRKN2* knockout cells in **c.** Values are pooled from four biologically independent experiments. One–way ANOVA followed by Dunnett's *post hoc* test, ***P<0.001 relative to wild type cells in **a** and **c**. (**b**) Oxygen consumption rates (OCR) and individual parameters for basal respiration, ATP production and maximal respiration. OCRs were measured at baseline and after the sequential addition of Oligomycin (Oligo, 1μM), FCCP (0.5μM) and Rotenone (Rot; 1μM) + Antimycin A (Ant; 1μM). Two tailed Student's *t*–test *P<0.05 and **P<0.001 relative to wild type cells, *n*=12 replicates pooled from four biologically independent experiments. (**d**) Representative images and quantification of the shape (expressed as circularity) of the mitochondrial network; *n*=173 mitochondria pooled from 21 wild type cells and *n*=77 mitochondria pooled from 10 *PINK1* KO cells and *n*=158 mitochondria pooled from 16 *PRKN2* KO cells. Values are pooled from two biologically independent experiments. Plots represent mean ± SEM. Scale bars are 10μm in **a** and in **b**, and 1μm (top) and 500nm (bottom) in **d**. NS: non–significant.

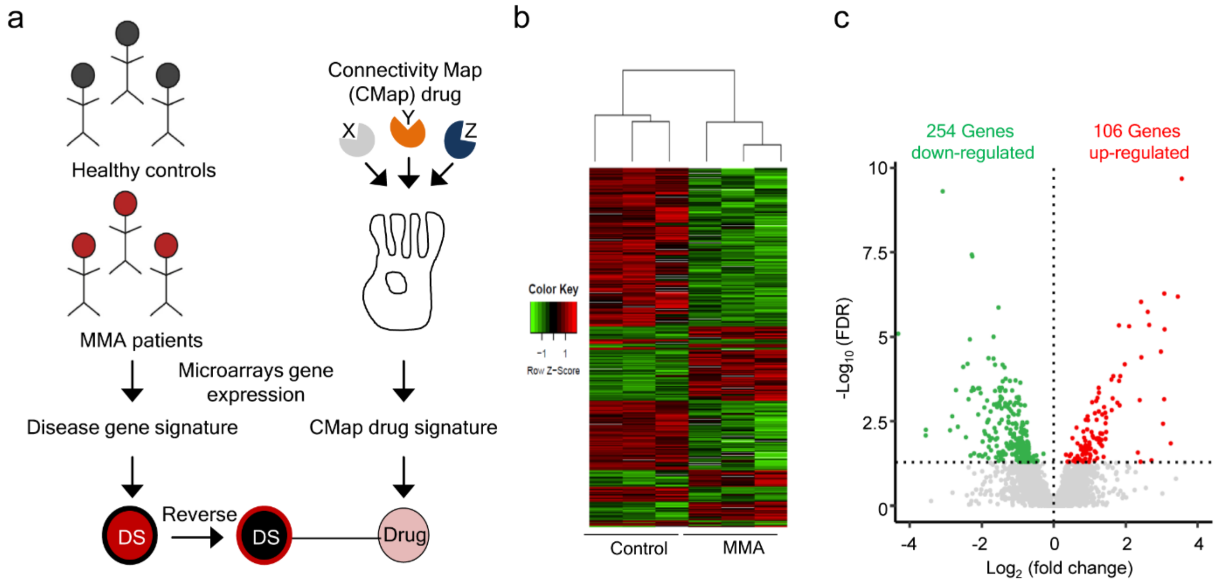

Supplementary Figure 12. Transcriptome profiling of control and MMA kidney cells. (**a**) The Mantra 2.0 tool concept. The connectivity map, which includes transcriptome–wide profiles following treatment of 1309 small molecules across five different cell lines, is compared against the disease gene signature derived from comparing the whole–transcriptome profiles between control and MMA diseased cells. This computational modelling captures compounds which are transcriptionally closer to the inverse diseased gene signature, identifying potential drug compound–targeted biological pathway candidates. (**b**) Hierarchical–clustering and heat map: representation of the 360 genes identified as significantly expressed between control and MMA kidney cells, *n*=3 biologically distinct samples per each group. Expression levels are color–coded and indicate the fold change in pairwise comparisons; upregulated and downregulated genes are presented in red and green dots, respectively. (**c**) Volcano plot of transcriptome–wide changes between control and MMA cells. Transcripts significantly changing [$P \leq 0.05$, log2 |fold change| $\geq \pm 1.5$] are represented by green (downregulated genes) and red (upregulated genes) dots, respectively.

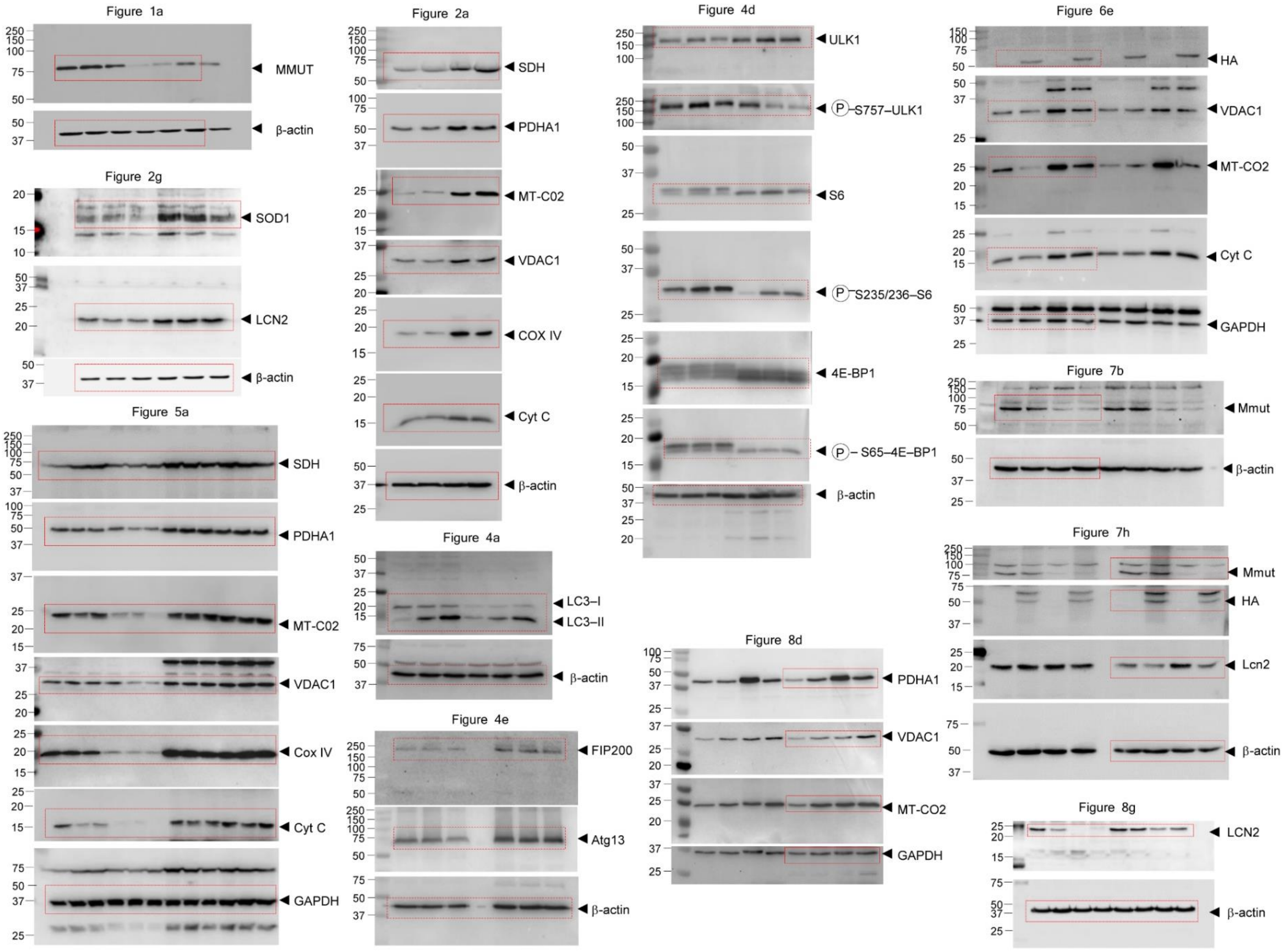

Supplementary Figure 13. Uncropped and unprocessed versions of western blots presented in the main and supplementary figures

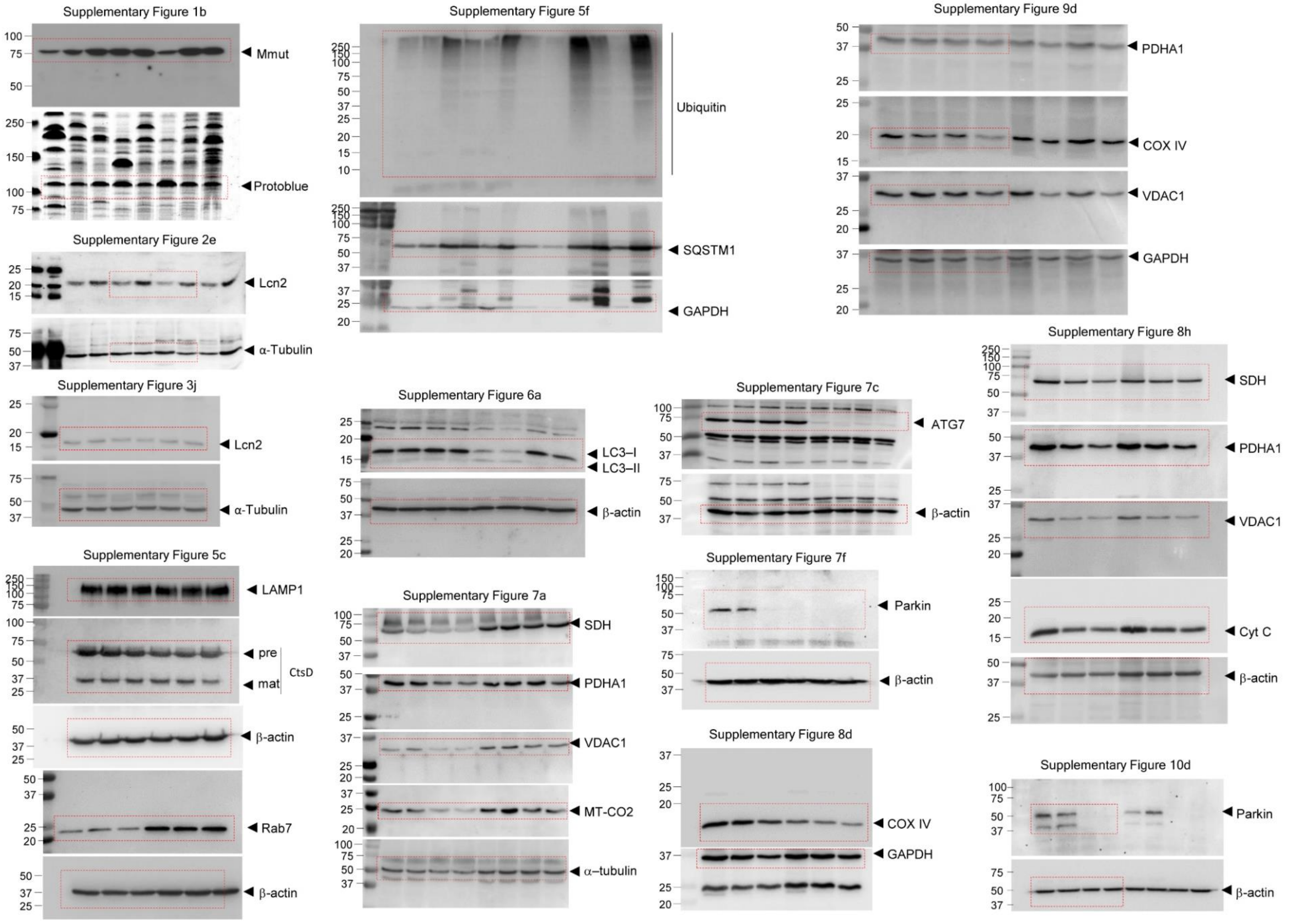

Supplementary Figure 13 (continued). Uncropped and unprocessed versions of western blots presented in the main and supplementary figures

Supplementary Table 1. Disease–causing mutations and MMUT enzyme activity in kidney cells derived from patients with MMA

Cell line	Class	Nucleotide change	Amino acid change	Enzymatic activity
MMA #1	mut^{o}	c.607C>A; c.1105C>T (exon 3)/(exon 4)	p.S288P/p.H386R	66±18
MMA #2	mut^{0}	c.862T>C (exon 4)	p.S288P	17±10
MMA #3	mut^{0}	c.982C>T (exon 4)	p.L328F	38±25
Control #1	-	-	-	3403±136
Control #2	-	-	-	7406±336
Control #3	-	-	-	3738±218

mut^{0}, complete deficiency;

Enzymatic activity expressed as pmol× min^{-1}×mg^{-1} of MMUT protein; the numbers represent the mean ± SEM of three independent experiments

Supplementary Table 2. Mouse primer pairs used for the gene expression analysis

Gene name	Forward primer (5'-3')	Reverse primer (5'-3')	PCR products (bps)	Efficiency
Gapdh	TGCACCACCAACTGCTTAGC	GGATGCAGGGATGATGTTCT	176	1.04 ± 0.03
Npsh2	GTCTAGCCCATGTGTCCAAA	CCACTTTGATGCCCCAAATA	162	1.03 ± 0.03
Slc12a3	CATGGTCTCCTTTGCCAACT	TGCCAAAGAAGCTACCATCA	148	1.01 ± 0.03
Slc12a1	CCGTGGCCTACATAGGTGTT	GGCTCGTGTTGACATCTTGA	154	0.99 ± 0.04
Aqp2	TCACTGGGTCTTCTGGATCG	CGTTCCTCCCAGTCAGTGT	147	1.03 ± 0.04
Slc5a2	TTGGGCATCACCATGATTTA	GCTCCCAGGTATTTGTCGAA	164	1.01 ± 0.03
Slc38a3	GTTATCTTCGCCCCCAACAT	TGGGCATGATTCGGAAGTAG	109	0.99 ± 0.02
Mmut	CAATGGCAGCAGTATTTGGA	GATCAGCCACTTTGGGAATC	150	0.99 ± 0.03
Pink	ATATGCTGCCCCCACACTAC	CAACTGCAAGGTCATCATGG	149	1.02 ± 0.02
Atg7	AGCTTGGCTGCTACTTCTGC	CTGCAGGACAGAGACCATCA	149	0.98 ± 0.03
Lcn2	ATGTCACCTCCATCCTGGT	GTGGCCACTTGCACATTGT	148	0.97 ± 0.03
Cox10	GGCAGATAAGCCCATTGCTA	TGACCCTCTTCAGTGGTGTG	156	1.02 ± 0.03

Supplementary Table 3. Human primer pairs used for the gene expression analysis

Gene name	Forward primer (5'-3')	Reverse primer (5'-3')	PCR products (bps)	Efficiency
GAPDH	GGGGCTCTCCAGAACATCAT	TCTAGACGGCAGGTCAGGT	149	1.01 ± 0.02
MFN1	ATATGGAAGACGTACGCAGAC	CCCCTGTGCTTTTTGCTTTC	146	1.03 ± 0.03
MFN2	TTGTCATCAGCTACACTGGC	AACCGGCTTTATTCCTGAGC	147	0.98 ± 0.03
DRP1	GGCGCTAATTCCTGTTCATAA	CAGGCTTTCTAGCACTGAGC	151	0.99 ± 0.03
LCN2	TCACCTCCGTCCTGTTTAGG	TGCTGGTTGTAGTTGGTGCT	157	1.02 ± 0.03
OPA1	GGCTCCTGACACAAAGGAAA	TCCTTCCATGAGGGTCCATT	150	1.01 ± 0.03
PINK1	CGCCAGTACCTTTGTGTGAA	GTCCAGCTCCACAAGGATGT	144	1.01± 0.03
mt–Keima	CAGGGCAACTGCTTCATCTA	CTTCAGGGCCATGTAGTCGT	159	0.99 ± 0.03

Supplementary Table 4. Zebrafish primer pairs used for the gene expression analysis

Gene name	Forward primer (5'-3')	Reverse primer (5'-3')	PCR products (bps)	Efficiency
mmut	GCAGATATCTTCGCCTACAC	CAATCGTGTAAGCCAACTCC	117	1.01 ± 0.04
actb1	TGAATCCCAAAGCCAACAGAG	TCACACCATCACCAGAGTCC	149	1.03 ± 0.04
b2m	GGGAAAGTCTCCACTCCGA	CGTTCTTCAGCAGTTCAATGG	129	0.99 ± 0.02
eef1a1a	TTCTCCGAGTATCCTCCTCTG	CTTCTCCACTCCTTTAATCACTCC	86	1.00 ± 0.02
g6pd	ATCTTCACTCCTCTCCTTCATCAG	ACCAGCTCATCAGCCTCAG	97	0.98 ± 0.04
hprt1	TGAAGAGGACACCGAGGAG	GTAGTCAAGTGCATATCCAACCA	91	1.01 ± 0.03
