# Supplementary material for: Impaired mitophagy links mitochondrial disease to epithelial stress in methylmalonyl-CoA mutase deficiency
Source: Nat Commun. 2020 Feb 20;11:970. doi: 10.1038/s41467-020-14729-8 (PMC7033137; doi:10.1038/s41467-020-14729-8)
Supplement: Supplementary file 2 — Description of Additional Supplementary Files [file 41467_2020_14729_MOESM2_ESM.pdf]

Description of Additional Supplementary Files

File Name: Supplementary Data 1
Description: Full list of probe set annotations corresponding to genes differently expressed between control and MMA patient−derived kidney cells. The up and downregulated genes are highlighted in red and in green, respectively
