# Supplementary material for: Impaired mitophagy links mitochondrial disease to epithelial stress in methylmalonyl-CoA mutase deficiency
Source: Nat Commun. 2020 Feb 20;11:970. doi: 10.1038/s41467-020-14729-8 (PMC7033137; doi:10.1038/s41467-020-14729-8)
Supplement: Supplementary file 4 — Reporting Summary [file 41467_2020_14729_MOESM4_ESM.pdf]

nature research

Corresponding author(s): DBPR NCOMMS-18-29937D

Last updated by author(s): Jan 21, 2020

Reporting Summary

Nature Research wishes to improve the reproducibility of the work that we publish. This form provides structure for consistency and transparency in reporting. For further information on Nature Research policies, see Authors & Referees and the Editorial Policy Checklist.

Statistics

For all statistical analyses, confirm that the following items are present in the figure legend, table legend, main text, or Methods section.

n/a	Confirmed	
☐	☒	The exact sample size (n) for each experimental group/condition, given as a discrete number and unit of measurement
☐	☒	A statement on whether measurements were taken from distinct samples or whether the same sample was measured repeatedly
☐	☒	The statistical test(s) used AND whether they are one- or two-sided<br>*Only common tests should be described solely by name; describe more complex techniques in the Methods section.*
☒	☐	A description of all covariates tested
☐	☒	A description of any assumptions or corrections, such as tests of normality and adjustment for multiple comparisons
☐	☒	A full description of the statistical parameters including central tendency (e.g. means) or other basic estimates (e.g. regression coefficient) AND variation (e.g. standard deviation) or associated estimates of uncertainty (e.g. confidence intervals)
☐	☒	For null hypothesis testing, the test statistic (e.g. F, t, r) with confidence intervals, effect sizes, degrees of freedom and P value noted<br>*Give P values as exact values whenever suitable.*
☒	☐	For Bayesian analysis, information on the choice of priors and Markov chain Monte Carlo settings
☒	☐	For hierarchical and complex designs, identification of the appropriate level for tests and full reporting of outcomes
☒	☐	Estimates of effect sizes (e.g. Cohen's d, Pearson's r), indicating how they were calculated

Our web collection on statistics for biologists contains articles on many of the points above.

Software and code

Policy information about availability of computer code

Data collection	All Ttools used for collecting data are listed and detailed in Methods section
Data analysis	All tools used for analyzing the data are listed and detailed in Methods section

For manuscripts utilizing custom algorithms or software that are central to the research but not yet described in published literature, software must be made available to editors/reviewers. We strongly encourage code deposition in a community repository (e.g. GitHub). See the Nature Research guidelines for submitting code & software for further information.

Data

Policy information about availability of data

All manuscripts must include a data availability statement. This statement should provide the following information, where applicable:

- Accession codes, unique identifiers, or web links for publicly available datasets
- A list of figures that have associated raw data
- A description of any restrictions on data availability

All uncropped immunoblots associated with Figures. 1a, 2a, 2g, 4a, 4d, 4e, 5a, 6e, 7b, 7h, 8d, 8g and Supplementary Figures. 1b, 2e, 3j, 5c, 5f, 6a, 7a, 7c, 7f, 8d, 9d, 8h, 10d are shown in Supplementary Figure 13. Source data underlying the figures 1-9 and Supplementary Figures 1-11 are provided as a Source Data file. Microarray data that support the findings of this study are provided in Supplementary Table 2 and have been deposited in NCBI Gene Expression Omnibus (GEO) with accession number GSE120683. Additional data supporting the findings of this study are available from corresponding author on reasonable request.

Field-specific reporting

Please select the one below that is the best fit for your research. If you are not sure, read the appropriate sections before making your selection.

☒ Life sciences ☐ Behavioural & social sciences ☐ Ecological, evolutionary & environmental sciences

For a reference copy of the document with all sections, see nature.com/documents/nr-reporting-summary-flat.pdf

Life sciences study design

All studies must disclose on these points even when the disclosure is negative.

Sample size	No statistical methods were used to predetermine the sample size. The sample size was selected based on commonly adopted standards in the field, resulting in statistically meaningful comparisons.
Data exclusions	No data were excluded from the analysis.
Replication	Experiments in Figure 9c were performed once. All of the experiments reported here in this study were independently performed two to three times or more as indicated in the legends.
Randomization	No randomization was applied
Blinding	The investigators were not blinded to allocation during the experiments and outcome assessment.

Reporting for specific materials, systems and methods

We require information from authors about some types of materials, experimental systems and methods used in many studies. Here, indicate whether each material, system or method listed is relevant to your study. If you are not sure if a list item applies to your research, read the appropriate section before selecting a response.

Materials & experimental systems

n/a	Involved in the study	
☐	☐	Antibodies
☐	☒	Eukaryotic cell lines
☒	☐	Palaeontology
☐	☒	Animals and other organisms
☐	☒	Human research participants
☒	☐	Clinical data

Methods

n/a	Involved in the study	
☒	☐	ChIP-seq
☒	☐	Flow cytometry
☒	☐	MRI-based neuroimaging

Antibodies

Antibodies used	All antibodies used in this study are listed in methods sections
Validation	All antibodies used in our study have been previously characterized and validated by biological strategies. Detailed information can be found on the website from manufactures. This information has been listed in the methods section.

Eukaryotic cell lines

Policy information about cell lines

Cell line source(s)	Kidney cells from healthy donors and from patients with MMA were provided by Dr. Stefan Koelker (Division of Inherited Metabolic Diseases, University Children's Hospital Heidelberg).<br>Primary cultures of micro-dissected proximal tubules derived from mouse kidneys were developed in the Devuyst lab at Institute of Physiology, University of Zurich.<br>CRISPR/Cas9-medaited PRKN2 (HZGHC003208c002) and PINK1 (HZGHC000798c008) knockout (KO) cell line were generated and purchased from Horizon Discovery (www.horizondiscovery.com).<br>HeLa cell line was kindly provided by Dr. L. Borsig, University of Zurich.
Authentication	The control and patient cells were authenticated by their genotype and by measuring the levels of methylmalonic acid (MA) and the amount of MMUT protein and its enzyme activity.<br>Primary proximal tubule cells were authenticated by their gene profile and by assessing their specialized functions such as endocytic uptake of albumin and transferrin ligands.<br>PRK2 and PINK1 KO cells were subjected to PCR analysis, followed by Sanger sequencing to identify CRISPR–Cas9 induced–deletion.

Mycoplasma contamination	All cell lines were routinely tested for mycoplasma using a commercial kit (Lonza) and verified as mycoplasma-negative.
Commonly misidentified lines (See ICLAC register)	The cells lines used here do not appear in the database of commonly misidentified cell lines (International Cell Line Authentication Committee), except the HeLa cell line that was analysed as negative control for the detection of Parkin expression in Supplementary Figure 7f.

Animals and other organisms

Policy information about studies involving animals; ARRIVE guidelines recommended for reporting animal research

Laboratory animals	The mice (C57BL/6 background) bearing floxed Cox10 (Cox10 fl/fl) alleles, in which the exon 6 is flanked by two loxP sequences, were kindly provided by Dr. Moraes (Department of Neurology, University of Miami).<br>The mice (C57BL/6 background) bearing floxed Atg7 (Atg7 fl/fl) alleles were kindly provided by the RIKEN BRC through the National–Bio–Resource Project of the MEXT, Japan.<br>The mice (C57BL/6 background) bearing germline Prkn2 and Pink1 knockout were purchased from Jackson Laboratory.<br>The mice (C57BL/6 background) bearing Mut floxed allele, in which exon 3 of the Mut gene is flanked by 2 loxP sequences, or carrying M698K point mutation in the Mut gene were generated and purchased from Polygene (Switzerland).<br>mut-KO zebrafish larvae (TL/TU backgorund) were generated in Devuyst lab at the Institute of Physiology, University of Zurich, Switzerland.
Wild animals	No wild animals were involved in this study.
Field-collected samples	This study did not involve samples collected from the field.
Ethics oversight	Animal care and experimental procedures were approved by the institutional animal care and use committee at Canton Zurich in accordance with the ethical guidelines existing at University of Zurich, Switzerland.

Note that full information on the approval of the study protocol must also be provided in the manuscript.

Human research participants

Policy information about studies involving human research participants

Population characteristics	Human kidney biopsies were obtained from a patient with clinical diagnosis of MMA and from a healthy control (non–transplanted, normal human kidney) who had given consent to tissue utilization for research purpose.
Recruitment	There were no recruitment biases that impact the results of our analyses.
Ethics oversight	Informed consent was obtained, and the use of the human biopsy samples was in accordance with the ethical regulations at Bambino Gesù Children's Hospital and approved by the EURenOmics consortium (FP7, 2007–2013, grant agreement no. 305608) and by the institutional review board at Bambino Gesù Children's Hospital.

Note that full information on the approval of the study protocol must also be provided in the manuscript.
